# Distinct pathophysiological mechanisms of *CEP152* variants in microcephaly and brain abnormalities

**DOI:** 10.1038/s44321-026-00427-3

**Published:** 2026-05-05

**Authors:** Nanako Hamada, Lama AlAbdi, Tomoko Uehara, Looprasertkul Sasikarn, Takuma Nishijo, Reut Suliman-Lavie, Mais O Hashem, Majid Alfadhel, Shatha Alhefdhi, Brahim Tabarki, Malak Alghamdi, Ikuko Iwamoto, Toshiki Takenouchi, Kenjiro Kosaki, Sagiv Shifman, Seiji Mizuno, Nobuhiko Ohno, Fowzan S Alkuraya, Koh-ichi Nagata

**Affiliations:** 1 Department of Molecular Neurobiology, Institute for Developmental Research, Aichi Developmental Disability Center, Kasugai, 480-0392 Japan; 2 Center for Genomic Medicine, King Faisal Specialist Hospital and Research Center, Riyadh, 11211 Saudi Arabia; 3 Central Hospital, Aichi Developmental Disability Center, Kasugai, 480-0392 Japan; 4 Center for Medical Genetics, Keio University Graduate School of Medicine, Tokyo, 160-8582 Japan; 5 Section of Electron Microscopy, Supportive Center for Brain Research, National Institute for Physiological Sciences, National Institutes of Natural Sciences, Okazaki, 444-8787 Japan; 6 Department of Anatomy, Division of Histology and Cell Biology, School of Medicine, Jichi Medical University, Shimotsuke, 329-0498 Japan; 7 Department of Genetics, The Alexander Silberman Institute of Life Sciences The Hebrew University of Jerusalem, Jerusalem, 91904 Israel; 8 Medical Genomics Research Department, King Abdullah International Medical Research Center, King Abdullah Specialized Children’s Hospital, King Abdulaziz Medical City, Ministry of National Guard Health Affairs, Riyadh, Saudi Arabia; 9 King Saud Bin Abdulaziz University for Health Sciences, King Abdullah Specialized Children’s Hospital, King Abdulaziz Medical City, Ministry of National Guard Health Affairs, Riyadh, Saudi Arabia; 10 Genetics and Precision Medicine Department, King Abdullah Specialized Children’s Hospital, King Abdulaziz Medical City, Ministry of National Guard Health Affairs, Riyadh, Saudi Arabia; 11 Medical Genetics Division, Department of Pediatrics, King Khalid University Hospital, King Saud University, Riyadh, 12372 Saudi Arabia; 12 Division of Neurology, Department of Pediatrics, Prince Sultan Military Medical City, Riyadh, Saudi Arabia; 13 Medical Genetics Division, Pediatrics Department, College of Medicine, King Saud University, Riyadh, Saudi Arabia; 14 Department of Pediatrics, Keio University Graduate School of Medicine, Tokyo, 160-8582 Japan; 15 Division of Ultrastructural Research, National Institutes of Natural Sciences, Okazaki, 444-8787 Japan; 16 Lifera Omics, Riyadh, Saudi Arabia; 17 Department of Neurochemistry, Nagoya University Graduate School of Medicine, Nagoya, 466-8550 Japan

**Keywords:** Genetics, Gene Therapy & Genetic Disease, Neuroscience

## Abstract

CEP152 is essential for centriole function and neurodevelopment, and pathogenic recessive variants in *CEP152* cause primary microcephaly. We identified new compound heterozygous *CEP152* variants, c.314 G > A,p.(W105*) and c.2689 A > T,p.(K897*), in a microcephalic patient and analyzed them alongside a homozygous variant c.95 A > C,p.(Q32P) associated with severe microcephaly with marked gyral simplification. In vitro assays revealed distinct effects: p.K897* prevented centrosomal localization, p.W105* led to protein degradation, and p.Q32P retained centrosomal targeting but disrupted binding to Polo-like kinase 4, a key centriole biogenesis kinase and CEP152 partner. In vivo, both Cep152^W105*/K897*^ and Cep152^Q32P/Q32P^ knock-in mice displayed microcephaly; notably, Cep152^Q32P/Q32P^ mice also exhibited severe cortical defects during brain development. Cellular analyses revealed centrosome dysfunction, mitotic errors, and increased apoptosis, which were exacerbated in Cep152^Q32P/Q32P^ brains. Morphological examination, including electron microscopy, further demonstrated structural abnormalities of the centrosomes and centrioles in Cep152^Q32P/Q32P^ brains. Electrophysiological and gene expression analyses confirmed variant-specific neuronal impairments, which correlate with clinical severity. Collectively, these findings demonstrate that distinct *CEP152* variants disrupt neurodevelopment through different mechanisms, thereby explaining the spectrum of microcephaly severity and associated phenotypes.

## Synopsis

Distinct CEP152 variants drive primary microcephaly through differential disruption of centrosome function and PLK4 interaction, leading to variant-specific transcriptomic reprogramming, neurogenesis defects, and cortical malformations.

- Two patient-derived CEP152 knock-in mouse models were generated, revealing variant-specific defects in centrosome integrity, PLK4 interaction, and protein stability.
- Both variants induced mitotic abnormalities and apoptosis in neural progenitors, whereas only Q32P caused severe cortical hypoplasia, callosal thinning, and ventriculomegaly.
- RNA-seq identified variant-specific transcriptional signatures, with Q32P mice showing dysregulation of neurogenesis, neuronal positioning, and synaptic pathway genes.
- Dendritic growth impairment and neuronal mispositioning were observed exclusively in Q32P mice, accompanied by pronounced gene expression changes.
- Opposing electrophysiological alterations were detected between models, establishing a genotype-mechanism-transcriptome-phenotype framework.

## Abstract (web summary)

Distinct CEP152 variants drive primary microcephaly through differential disruption of centrosome function and PLK4 interaction, leading to variant-specific transcriptomic reprogramming, neurogenesis defects, and cortical malformations.

## Graphical abstract

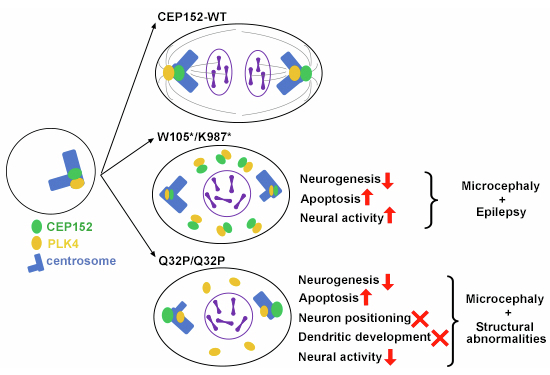

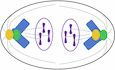

The paper explained **Problem**

Primary microcephaly is a rare congenital disorder characterized by a markedly reduced brain size, typically accompanied by intellectual disability and, in some cases, additional structural brain abnormalities. Pathogenic variants in *CEP152* encoding a centrosomal protein are known to cause microcephaly and microcephalic primordial dwarfism. The underlying pathophysiological mechanisms have remained unclear.

**Results**

In this study, we identified novel compound heterozygous *CEP152* variants, c.314 G > A,p.(W105*) and c.2689 A > T,p.(K897*), in a microcephalic patient and analyzed them alongside a homozygous variant c.95 A > C,p.(Q32P) associated with severe microcephaly with marked gyral simplification, and then generated the first knock-in mouse models carrying these patient-derived mutations. We demonstrate that distinct *CEP152* variants disrupt brain development through different molecular mechanisms. The truncating variants primarily impair centrosome localization and protein stability, whereas the missense p.Q32P variant disrupts CEP152–Polo-like kinase 4 (PLK4) interaction and severely compromises centriole integrity. In vivo, these variant-specific molecular defects result in differential degrees of mitotic abnormalities, apoptosis, cortical disorganization, synaptic dysfunction, and electrophysiological alterations. Notably, the severity of the mouse phenotypes closely mirrors the clinical manifestations observed in patients.

**Impact**

Our findings provide a mechanistic explanation for the clinical heterogeneity associated with *CEP152*-related microcephaly. By establishing genotype–mechanism–phenotype correlations, this study improves diagnostic interpretation of *CEP152* variants and may help refine genetic counseling. Furthermore, the identification of variant-specific disruption of the CEP152–PLK4 pathway highlights potential targets for future therapeutic strategies aimed at modulating centrosome function and neurodevelopmental outcomes.

## Introduction

The centrosome, the primary microtubule-organizing center in animal cells, consists of a pair of centrioles surrounded by a protein-rich pericentriolar material (PCM). It plays a crucial role in cell division, polarity, and motility (Bettencourt-Dias and Glover, 2007; Conduit et al, 2015; Takeda et al, 2020). Recent studies have linked variants in centriolar and centrosomal protein-encoding genes to developmental disorders, including microcephaly and dwarfism (Bettencourt-Dias et al, 2011; Davis and Katsanis, 2012).

Centrosomal protein 152 (CEP152), encoded by the *CEP152* gene, is a ~195 kDa protein that contains five coiled-coil domains. CEP152 was first identified as a mammalian centrosome component through proteomic analysis (Nogales-Cadenas et al, 2009; Andersen et al, 2003) and is the mammalian ortholog of *Drosophila* asterless, a key regulator of cell division and tissue development (Varmark et al, 2007; Blachon et al, 2008). CEP152 plays a critical role in centriole biogenesis and centrosome function, acting as a scaffold for procentriole assembly by interacting with CEP63, CPAP/CENPJ/SAS-4, CEP192, CDK5RAP2/CEP215, WDR62, and PLK4 (polo-like kinase 4) (Dzhindzhev et al, 2010; Fırat-Karalar and Stearns, 2014; Kalay et al, 2011; Kodani et al, 2015; Sir et al, 2011; Firat-Karalar et al, 2014). Biochemical mapping shows that its C-terminal region mediates interactions with CEP63, CPAP, and CEP192 (Dzhindzhev et al, 2010; Sir et al, 2011; Firat-Karalar et al, 2014; Brown et al, 2013), while the N-terminal region binds PLK4 (Park et al, 2014; Sonnen et al, 2013). Additionally, PLK4 phosphorylates CEP152 (Boese et al, 2018), suggesting reciprocal regulation in centrosome formation and function.

CEP152 and its interacting partners are implicated in autosomal recessive primary microcephaly (MCPH: microcephaly primary hereditary), a rare neurogenic disorder associated with defective mitosis. MCPH is characterized by congenital microcephaly, accompanied by varying degrees of intellectual disability (ID), but lacks other syndromic features. In addition to primary microcephaly, variants in *CEP152* have also been identified in patients with microcephalic primordial dwarfism (MPD) (Kalay et al, 2011; Sir et al, 2011; Zhang et al, 2023; Hussain et al, 2012; Darvish et al, 2010; Shaheen et al, 2019; Alkuraya, 2015). MPD represents a distinct group of rare autosomal recessive disorders caused by single-gene variants and characterized by both intrauterine and postnatal growth retardation. Clinical features include short stature, distinctive facial features, microcephaly, and ID (Shanske et al, 1997).

In this study, we identified novel compound heterozygous variants, c.314 G > A,p.(W105*) and c.2689 A > T,p.(K897*) in *CEP152* in an individual with primary microcephaly. Additionally, we investigated their molecular impact alongside a homozygous variant, c.95 A > C,p.(Q32P), which we previously reported in one case (Shaheen et al, 2019) and newly identified in another in this study. These variants exhibited distinct phenotypic effects: the compound heterozygous variants were associated with simplified gyri, a hallmark of MCPH, whereas the homozygous variant led to severe microcephaly with marked gyral simplification and additional associated phenotypes. To explore their pathophysiological significance, we conducted in vitro analyses demonstrating that these variants differentially affect CEP152 protein stability, localization, and interaction with PLK4. To further assess their impact in vivo, we generated two mouse models, Cep152^W105*/K897*^ and Cep152^Q32P/Q32P^, representing the first *CEP152*-related disease models. Morphological, biochemical, cellular, electrophysiological, and gene expression analyses revealed that these mice recapitulated the severity and key clinical features observed in patients. Our findings highlight CEP152 as a critical determinant of not only brain size but also structure, with *CEP152* variants contributing to distinct neurodevelopmental outcomes through variant-specific mechanisms.

## Results

### Study subjects and identification of novel *CEP152* variants

#### Patient #1

**Figure 1:**
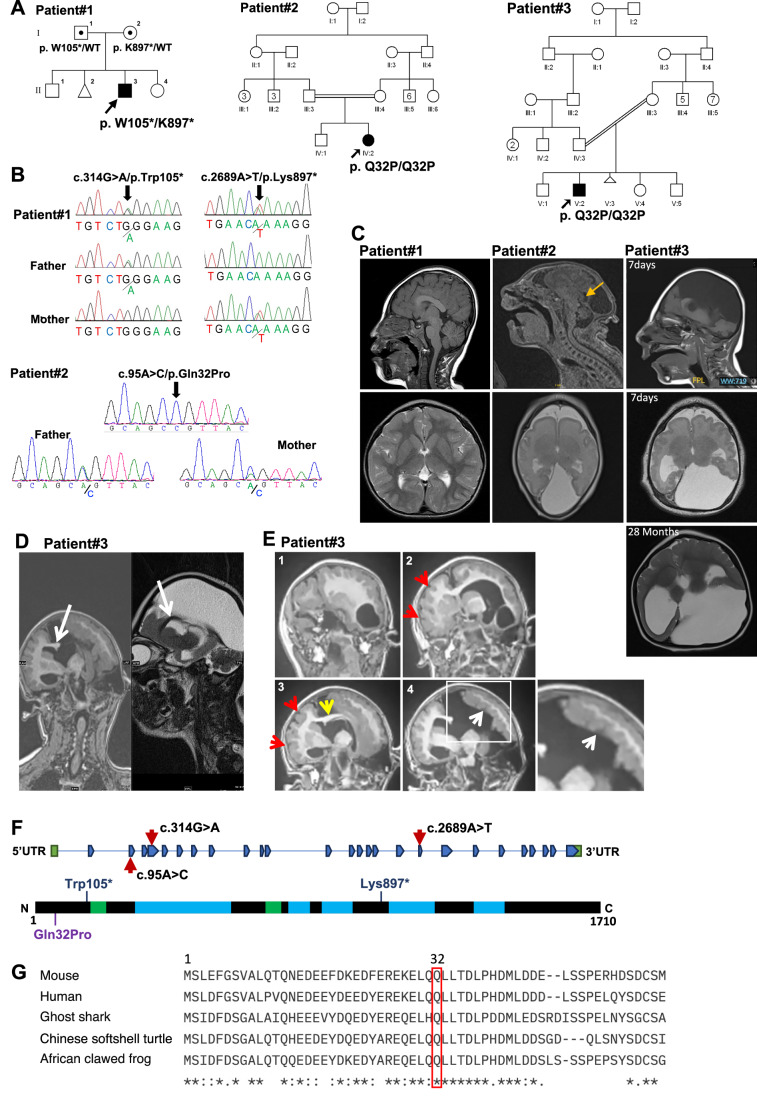
Genetic, clinical, and neuroimaging features associated with *CEP152* variants. (**A**) Family pedigree. Squares, circles, and a triangle represent male, female, and miscarriage, respectively. The proband is indicated by an arrow. The second child (#2) of the Japanese family (Patient #1) was terminated mid-term (18 weeks) due to fetal hydrocephalus. (**B**) Top panels; Electropherograms showing the c.314 G > A,p.(W105*) and c.2689 A > T,p.(K897*) variants detected in Patient #1. Bottom panels: Electropherograms of the affected child (Patient #2, homozygous for Q32P) and her parents (carriers for the same variant). (**C**) Left panels; Brain MRI images of Patient #1 (sagittal T1-weighted top and axial T2-weighted bottom) performed at 3 years of age. Middle panels: MRI images (sagittal top and axial bottom) of Patient #2 performed at 7 days of age. Hypogenesis of the corpus callosum (splenium) is indicated by an arrow. Right panels: MRI images (sagittal top and axial bottom two panels) of Patient #3 performed at 7 days and 28 months of age. (**D**) Axial and sagittal MRI images of Patient #3. Arrows indicate hypoplasia of the splenium (left) and body (right) of the corpus callosum. (**E**) Four serial coronal MRI sections of Patient #3. Red arrowheads indicate a mildly simplified gyral pattern in the right hemisphere. The yellow arrowhead indicates hypoplasia of the corpus callosum. The white arrowhead indicates polymicrogyria in the left parieto-occipital cortex. (**F**) Schematic representation of human *CEP152* gene (top) and polypeptide (bottom) sequence (aa1-1710; NM_001194998) showing the coding variants analyzed in this study. The five coiled-coil domains (blue) and two compositional bias domains (green) are indicated. (**G**) Multiple alignment of CEP152 protein sequences from mouse (Mus musculus), human (Homo sapiens), Callorhinchus milii (Ghost shark), Pelodiscus sinensis (Chinese softshell turtle), and Xenopus laevis (African clawed frog) was performed using Clustal Omega. The alignment shows the region encompassing the mutation site, showing its evolutionary conservation. An asterisk (*) indicates identical amino acid residues across all species. A colon (:) and a period (.) indicate conserved substitutions with strongly and weakly similar properties, respectively. The mutated residue (Q32 in human CEP152) is highlighted by a red box.

**Figure EV1:**
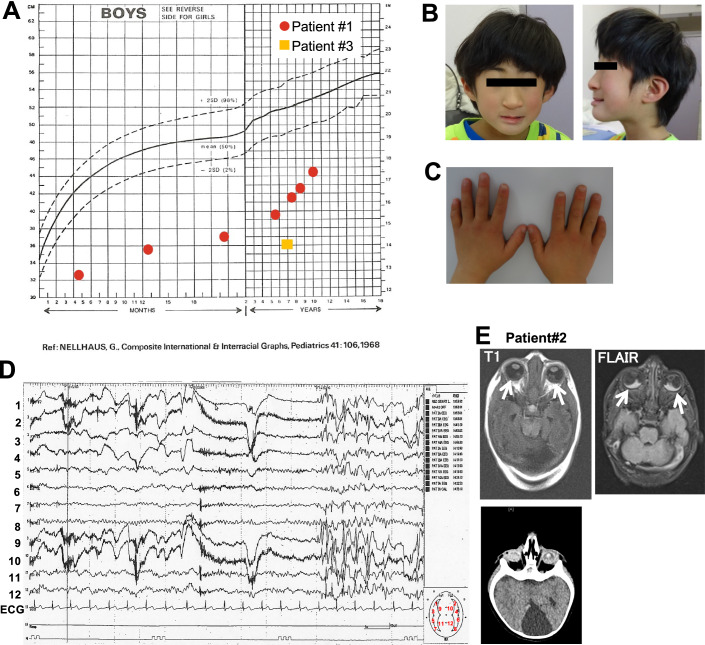
Clinical characteristics of the patients. (**A**) OFC measurements of Patients #1 and #3 plotted against population mean values. (**B**, **C**) Facial features (**B**) and clinodactyly of the fifth finger (**C**) of Patient #1 at 6 years of age. Hypotelorism, short palpebral fissures, micrognathia, a broad nasal bridge, and low-set ears were observed. Informed consent for publication of these photographs was obtained from the patient’s parents. (**D**) Electroencephalogram (EEG) of Patient #1 showing irregular spike-and-wave complexes localized to the frontal and temporal regions. (**E**) MRI (upper) and CT (bottom) images of Patient #2 demonstrating bilateral retinal detachment due to persistent hyperplastic primary vitreous.

We clinically evaluated a Japanese male patient with primary microcephaly, born to non-consanguineous parents. Trio-based whole-exome sequencing of genomic DNA from peripheral blood samples revealed novel compound heterozygous nonsense variants in exons 5 and 19 of the *CEP152* gene (NM_001194998.1): a paternally inherited c.314 G > A,p.(W105*) variant and a maternally inherited c.2689 A > T,p.(K897*) variant (Fig. 1A). These findings were confirmed by Sanger sequencing (Fig. 1B, top panels). A brain MRI at 3 years of age showed markedly reduced brain volume, particularly in the prefrontal cortex, with simplified gyri (Fig. 1C). Clinical characteristics of the patient were demonstrated (Fig. EV1A–C). EEG recordings revealed irregular spike-and-wave complexes in the frontal and temporal regions (Fig. EV1D), suggesting that these *CEP152* variants may lead to electrophysiological abnormalities in cortical neurons.

#### Patient #2

A 2-year-old Saudi Arabian female was diagnosed with congenital microcephaly. At birth, she showed a nearly closed anterior fontanel with severe microcephaly (birth occipitofrontal circumference [OFC] of 23.5 cm, −7.7 SD), which was below the lower limit of the standard head circumference chart and therefore could not be plotted. The antenatal history revealed symmetrical intrauterine growth restriction and an antenatal ultrasound identified microcephaly with a left-sided posterior cerebral cyst. The patient was delivered at 37 + 1 weeks gestation via spontaneous vaginal delivery. At birth, she cried immediately, had good tone and color, and her APGAR scores were 8 at 1 min and 9 at 5 min. Her birth weight was 1.4 kg (−3.9 SD), and she was admitted to the neonatal intensive care unit (NICU) for 15 days due to low birth weight and severe congenital microcephaly. During her hospital course, she showed good limb movement and good suckling. Sagittal MRI revealed severe hypogenesis of the corpus callosum (Fig. 1C, arrow), a simplified gyral pattern in both cerebral hemispheres, bilateral underdevelopment of the frontal horns, and a large cerebrospinal fluid-filled interhemispheric cyst in the posterior aspect of the falx cerebri, adjacent to the midline on the left side (Fig. 1C). MRI and CT scan images confirmed bilateral intraocular hemorrhage, consistent with retinal detachment (Fig. EV1E). Persistent hyperplastic primary vitreous could not be directly visualized on this sequence but might be considered based on the clinical course. The patient’s cardiovascular system was stable, and an echocardiogram showed a small patent foramen ovale and atrial septal defect with a left-to-right shunt. Abdominal ultrasound showed no congenital anomalies. Family history revealed consanguinity at the level of parents and grandparents (Fig. 1A). The patient has one healthy sibling, a 6-year-old brother, and there is no history of abortions in the family. Genetic testing was recommended to identify potential underlying causes of the severe microcephaly with marked gyral simplification. Chromosomal microarray analysis was normal. Whole-exome sequencing revealed a homozygous variant in the *CEP152* gene, c.95 A > C,p.(Q32P); Chr15(GRCh37):g.49090241 T > G (Fig. 1B, bottom panels).

#### Patient #3

A 7-year-old Saudi Arabian male (Fig. 1A) who presents with a history of global developmental delay, microcephaly, and spasticity. He is blind but has intact hearing. He is non-verbal and can sit without support but is unable to stand or walk. On physical examination, the patient’s weight was 12.1 kg (−4.2 SD), and OFC of 36 cm (−12 SD). He exhibited small eyes, a long nose, a ‘bird-headed’ facial appearance, and generalized spasticity. Investigations included MRI at 7 days of age, which revealed severe microcephaly, lateral ventricle dilatation, and an interhemispheric cyst (Fig. 1C). Axial and sagittal MRI showed hypoplasia of the body and splenium of the corpus callosum (Fig. 1D, arrows). Sequential coronal MRI sections revealed a mildly simplified gyral pattern in the right hemisphere, hypoplasia of the corpus callosum, and polymicrogyria (Fig. 1E, red arrows, yellow arrow, and white arrows, respectively). A skeletal survey revealed microcephaly and developmental dislocation of the hip. Electroretinography and flash visual evoked potentials suggested bilateral retinal and visual pathway involvement. While ophthalmological imaging was not available, the written report in the medical chart stated that the patient was blind due to bilateral retinal detachment associated with persistent hyperplastic primary vitreous. Chromosomal microarray analysis was normal. Whole-exome sequencing revealed a homozygous variant in the *CEP152* gene, c.95 A > C,p.(Q32P); Chr15(GRCh37):g.49090241 T > G. MRI performed at 28 months of age showed a significant interval increase in cyst size and ventricular dilatation (Fig. 1C).

A schematic representation of the human *CEP152* gene and polypeptide sequence, summarizing the coding variants analyzed, is presented (Fig. 1F). Multiple sequence alignment of CEP152 across several vertebrate species revealed that the Q32 residue is highly conserved (Fig. 1G), highlighting its potential functional importance. The lack of recognizable relatedness between Patients #2 and #3, despite sharing the same variant and surrounding haplotype, is consistent with the founder nature of the variant. This allowed us to use it as strong evidence supporting the pathogenicity of the variant (AlAbdi et al, 2024), along with other criteria such as a high constraint score (CADD 25) and its extreme rarity in gnomAD (one single heterozygous individual). A comparison of the clinical phenotypes between our patients and previously reported cases is presented in Table EV1.

### Biological and biochemical properties of the *CEP152* variants p.K897*, p.W105*, and p.Q32P

**Figure 2:**
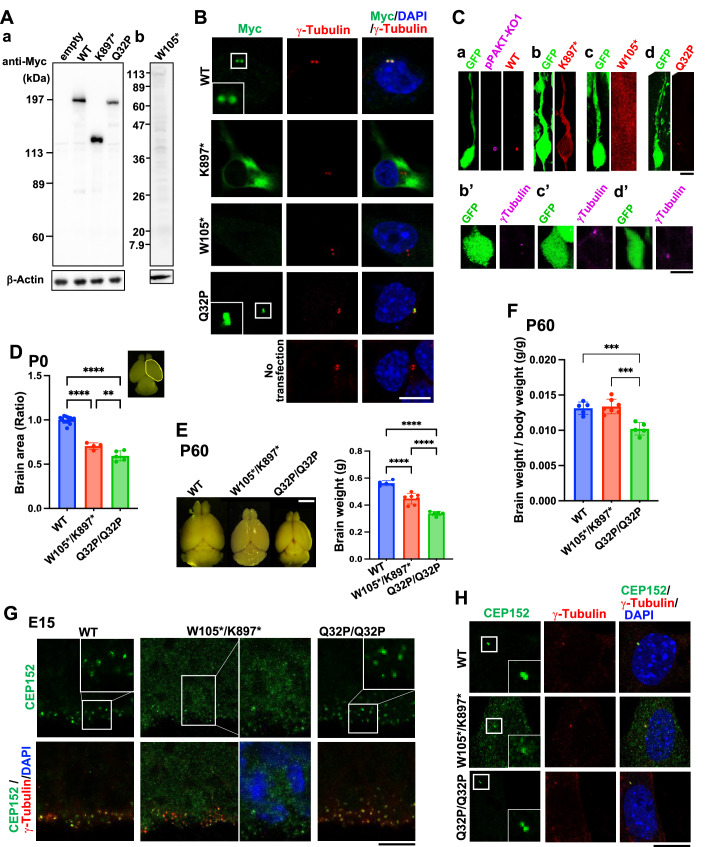
Detection and subcellular localization of *CEP152* variants and generation of model mice. (**A**) Expression of CEP152, CEP152-K897*, -W105*, and -Q32P in N2A cells. Cells were transfected with pCAG-Myc (empty vector), pCAG-Myc-CEP152 (WT), or each variant construct (p.K897*, p.W105*, or p.Q32P) at 0.3 µg per well. After 48 h, cells were harvested and subjected to SDS-PAGE: 7.5% gel for WT, CEP152-K897*, and -Q32P (a) and 15% gel for CEP152-W105* (b). Western blotting was performed using anti-Myc. β-actin was used as a loading control and was visualized on a 10% gel. Full uncropped data are shown in Fig. EV2. (**B**) Subcellular distribution of Myc-CEP152 and the variants in N2A cells. Cells were transfected and cultured as in (**A**). After fixing with 4% PFA, cells were stained with anti-Myc (green) and anti-γ-tubulin (red). Nuclei were visualized with DAPI (blue). Boxed areas are magnified in insets. (**C**) Subcellular localization of Myc-CEP152 and its variants in cortical neurons in layer II-III in vivo. pCAG-EGFP (0.5 µg) was co-electroporated in utero into the VZ progenitors at E14.5 with pCAG-PACKmKO1 (a centrosome marker) and either pCAG-Myc-CEP152 (WT, a), -CEP152-K897* (b and b’), -W105* (c and c’), or -Q32P (d and d’) (0.5 µg each). Coronal sections were prepared at P0 and stained for GFP (green) and Myc-tag (red, upper panels), or GFP (green) and γ-tubulin (magenta, lower panels). (**D**) Quantification of the relative cerebral cortex area in dissected brains from WT, Cep152^W105*/K897*^, and Cep152^Q32P/Q32P^ mice at P0. The analyzed area is marked with a line on a representative brain image. Data were presented as mean ± SD. *n* = 13, 4, and 5 for WT, Cep152^W105*/K897*^, and Cep152^Q32P/Q32P^, respectively. Tukey–Kramer LSD, **p = 0.0029, ****p < 0.0001. (**E**) Quantification of whole brain weight in WT, Cep152^W105*/K897*^, and Cep152^Q32P/Q32P^ mice at P60. Data were presented as mean ± SD. *n* = 5, 7, and 5 for WT, Cep152^W105*/K897*^, and Cep152^Q32P/Q32P^, respectively. Representative images of dissected brains are shown. Tukey–Kramer LSD, ****p < 0.0001. (**F**) Analysis of the brain-to-body weight ratio of WT, Cep152^W105*/K897*^, and Cep152^Q32P/Q32P^ mice at P60. Data were presented as mean ± SD. *n* = 5, 7, and 5 for WT, Cep152^W105*/K897*^, and Cep152^Q32P/Q32P^, respectively. Tukey–Kramer LSD, ***p = 0.0007 (WT vs Cep152^Q32P/Q32P^), ***p = 0.0002 (Cep152^W105*/K897*^ vs Cep152^Q32P/Q32P^). (**G**) Cortical slices (E15.5) from WT, Cep152^W105*/K897*^, and Cep152^Q32P/Q32P^ mice were double-stained with anti-CEP152-N (green) together with anti-γ-tubulin (red) or DAPI (blue). (**H**) MEFs from each genotype were double-stained as in (**G**). Nuclei were visualized with DAPI (blue). Boxed areas are magnified in insets. Scale bars: 5 μm (**B**, **C**), 10 μm (**G**, **H**). Source data are available online for this figure.

To investigate the biochemical and cellular characteristics of *CEP152* variants (CEP152-K897*, CEP152-W105*, and CEP152-Q32P), we conducted a series of in vitro experiments. Since variants in neuronal genes can affect the steady-state levels of their polypeptide products (Hamada et al, 2023, 2021, 2017; Noda et al, 2021), we evaluated the stability of each variant by Western blotting. When Myc-CEP152, CEP152-K897*, or CEP152-Q32P was transiently expressed in N2A cells, their expression levels were comparable to the wildtype, indicating that these variants do not affect protein stability (Fig. 2Aa). In contrast, Myc-CEP152-W105* was virtually undetectable under the same conditions, indicating that this variant is unstable (Fig. 2Ab). Next, we assessed the subcellular localization of Myc-CEP152 and its variants in N2A cells, which possess an intact centrosome structure (Fig. 2B). Under conditions where wild-type CEP152 localized to the centrosome and was co-immunostained with γ-tubulin, CEP152-K897* was diffusely distributed in the cytoplasm, while CEP152-W105* was undetectable (Fig. 2B). In contrast, CEP152-Q32P co-localized with γ-tubulin at the centrosome in a manner similar to the wild-type protein (Fig. 2B). The results for CEP152-K897* are reminiscent of a previous report showing that CEP152-R987* was abnormally distributed in the cytoplasm of osteosarcoma U2OS cells, where CEP152 typically localizes to the centrosome (Guernsey et al, 2010). To further investigate these variants’ subcellular distribution in cortical neurons during corticogenesis in vivo, we performed in utero electroporation on embryonic day 14.5 (E14.5) mice. Expression constructs encoding CEP152 and the variants were co-electroporated with pCAG-GFP and pCAG-PACKmKO1 into neural progenitors of the developing cerebral cortex. Immunostaining of electroporated brains collected at P0 revealed that both CEP152 and CEP152-Q32P accumulated at the centrosome in cortical neurons (Fig. 2Ca,d). In contrast, CEP152-K897* was diffusely distributed throughout the cytoplasm, while CEP152-W105* was virtually undetectable under the same conditions (Fig. 2Cb,c). Based on the γ-tubulin staining pattern, the PCM structure appeared to be preserved following expression of the three CEP152 variants (Fig. 2Cb′–d′). Notably, overexpression of CEP152 or its variants did not significantly affect the migration of E14.5-labeled cortical neurons, although suppression of *CEP152* has been reported to cause defective neuronal migration (Lu et al, 2018).

**Figure EV2:**
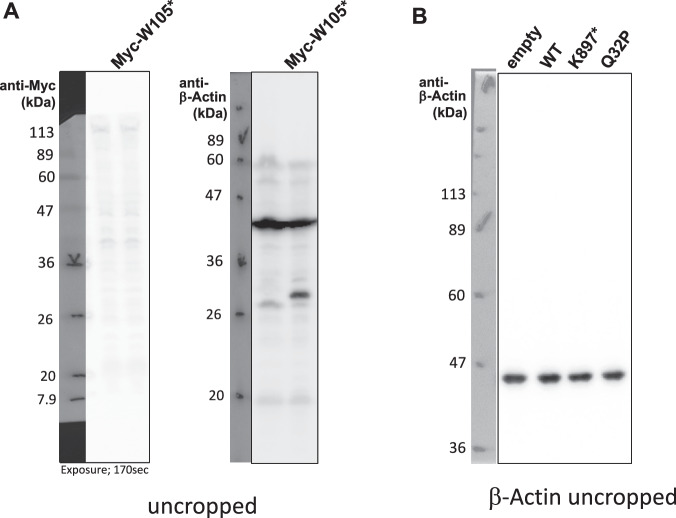
Full, uncropped Western blot images corresponding to Fig. 2A. (**A**) Full, uncropped Western blot images corresponding to Fig. 2A,b. (**B**) Full, uncropped Western blot images with anti-β-actin corresponding to Fig. 2A,a.

### Generation of mouse models recapitulating the compound heterozygous p.W105*/p.K897* and the homozygous p.Q32P variants

To investigate the impact of CEP152 variants on brain development, we generated the first CEP152 disease mouse models, Cep152^W105*/K897*^ and Cep152^Q32P/Q32P^, using CRISPR-Cas9. These models include frameshift mutations leading to premature truncations at E27 and E916, mimicking W105 and K897, respectively, as well as a missense mutation causing the amino acid substitution of Q32 with P. The Cep152^W105*/K897*^ and Cep152^Q32P/Q32P^ mice were born at sub-Mendelian ratios, suggesting compromised fitness. Both mutant groups exhibited primary microcephaly at birth (Fig. 2D), which persisted into adulthood (P60), as indicated by reduced brain weight (Fig. 2E). Additionally, both mutant mice also exhibited weight reduction, consistent with the symptoms frequently associated with *CEP152* variants (Table EV1). Notably, Cep152^Q32P/Q32P^ mice displayed a more severe microcephaly phenotype than the Cep152^W105*/K897*^ mice. Furthermore, Cep152^Q32P/Q32P^ exhibited a significantly reduced brain-to-body weight ratio compared to controls and Cep152^W105*/K897*^ (P60) (Fig. 2F). Collectively, Cep152^W105*/K897*^ and Cep152^Q32P/Q32P^ mice recapitulated the microcephaly phenotype observed in the patients.

**Figure EV3:**
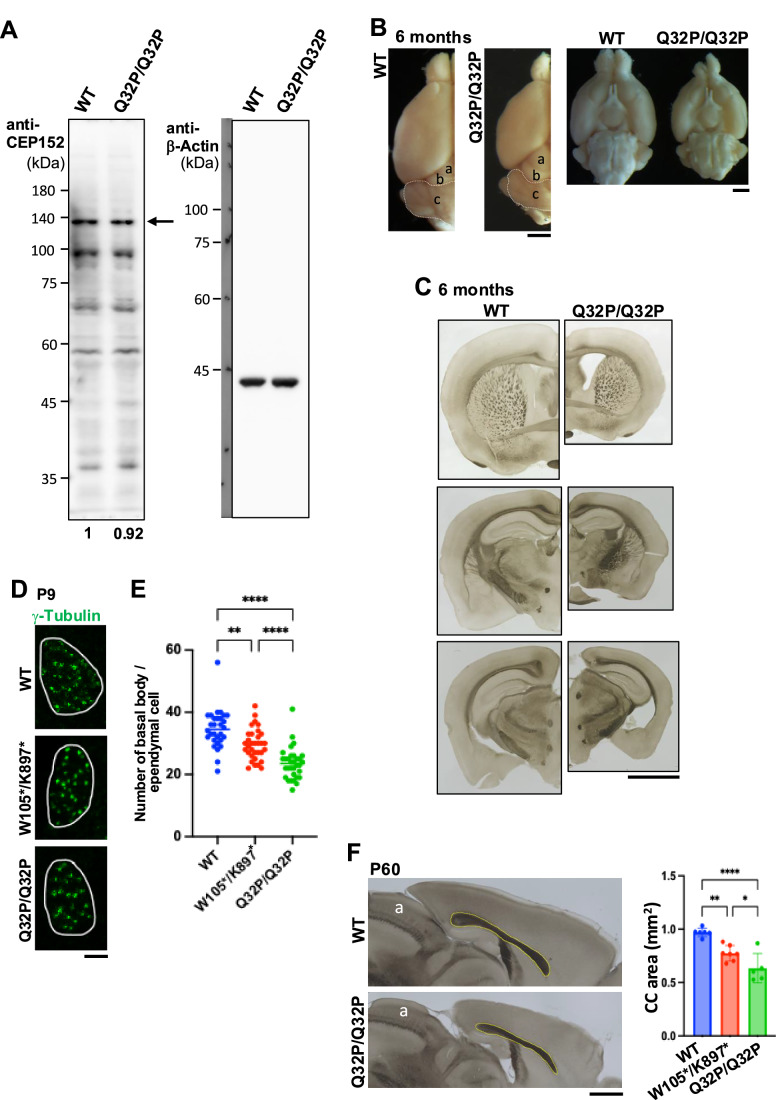
Expression of variant proteins in Cep152^W105*/K897*^ and Cep152^Q32P/Q32P^ mice. (**A**) Expression of endogenous CEP152 and CEP152-Q32P in the brains of WT and Cep152^Q32P/Q32P^ mice. Whole-tissue extracts were subjected to SDS-PAGE (7.5% gel), followed by western blotting with an anti-CEP152-C. β-actin was used as a loading control and visualized on a 10% gel. Band intensities of CEP152 proteins were quantified and normalized to β-actin levels. Full uncropped blots are shown. (**B**) Dorsal and ventral views of whole brains from WT and Cep152^Q32P/Q32P^ mice at 6 months of age. Regions used for macroscopic comparison are indicated (a superior colliculus; b inferior colliculus; c cerebellum outlined by a dotted line). Note that both the cerebral cortex and cerebellum appear markedly reduced in size compared with the midbrain structures, resulting in unusually prominent exposure of the superior and inferior colliculi. This gross phenotype is consistent with that observed in Fig. 3C. (**C**) Vibratome coronal sections from WT and Cep152^Q32P/Q32P^ mice at 6 months, showing global brain morphology. (**D**) Immunostaining of basal bodies using anti–γ-tubulin at the base of motile cilia in ependymal cells lining the ventricular surface in WT, Cep152^W105*/K897*^, and Cep152^Q32P/Q32P^ mice at P9. Basal bodies were visualized as markers of individual motile cilia. Individual cells are indicated by white outlines. (**E**) Quantification of (**D**). Basal bodies were used to quantify motile cilia number. Data were presented as mean ± SD. *n* = 4 animals per genotype. Cell counts: WT, 30; Cep152^W105*/K897*^, 33; Cep152^Q32P/Q32P^, 32. Tukey–Kramer LSD, **p = 0.0011, ****p < 0.0001. (**F**) Corpus callosum morphology from vibratome sagittal sections at P60. The outlined region (yellow) indicates the area used for quantification. The bar graph shows the callosal area in WT and Cep152^Q32P/Q32P^ mice. Data were presented as mean ± SD. *n* = 6, 7, 6, for WT, Cep152^W105*/K897*^, and Cep152^W105*/K897*^, respectively. Tukey–Kramer LSD, *p = 0.0398, **p = 0.0031, ****p < 0.0001. Scale bars: 5 µm (**D**), 500 µm (**F**), 2 mm (**B**, **C**).

We then analyzed the expression profiles of CEP152-Q32P and CEP152-K897* in each mouse model. Western blotting using a C-terminal antibody (anti-CEP152-C) revealed that the expression levels of wild-type CEP152 and CEP152-Q32P were comparable in brains (P30) of control and Cep152^Q32P/Q32P^ mice (Fig. EV3A). The expression level of CEP152-K897* was not assessed, as anti-CEP152-C detects epitopes located in the C-terminus of CEP152, which is absent in the truncated variant. Next, the subcellular localization of endogenous variants was examined using an N-terminal antibody (anti-CEP152-N) in progenitor cells in the ventricular zone (VZ) during corticogenesis (E15.5), as well as in mouse embryonic fibroblasts (MEFs) from each genotype. As a result, CEP152-K897* appeared to be mainly distributed in the cytoplasm, whereas CEP152-Q32P was dominantly enriched at the centrosome (Fig. 2G,H), recapitulating the overexpression results. It should be noted that anti-CEP152-N failed to detect CEP152 by western blotting (available upon request), suggesting that this antibody recognizes conformational rather than linear epitopes.

### Cep152^W105*/K897*^ and Cep152^Q32P/Q32P^ mice exhibit microcephaly and defective brain development

**Figure 3:**
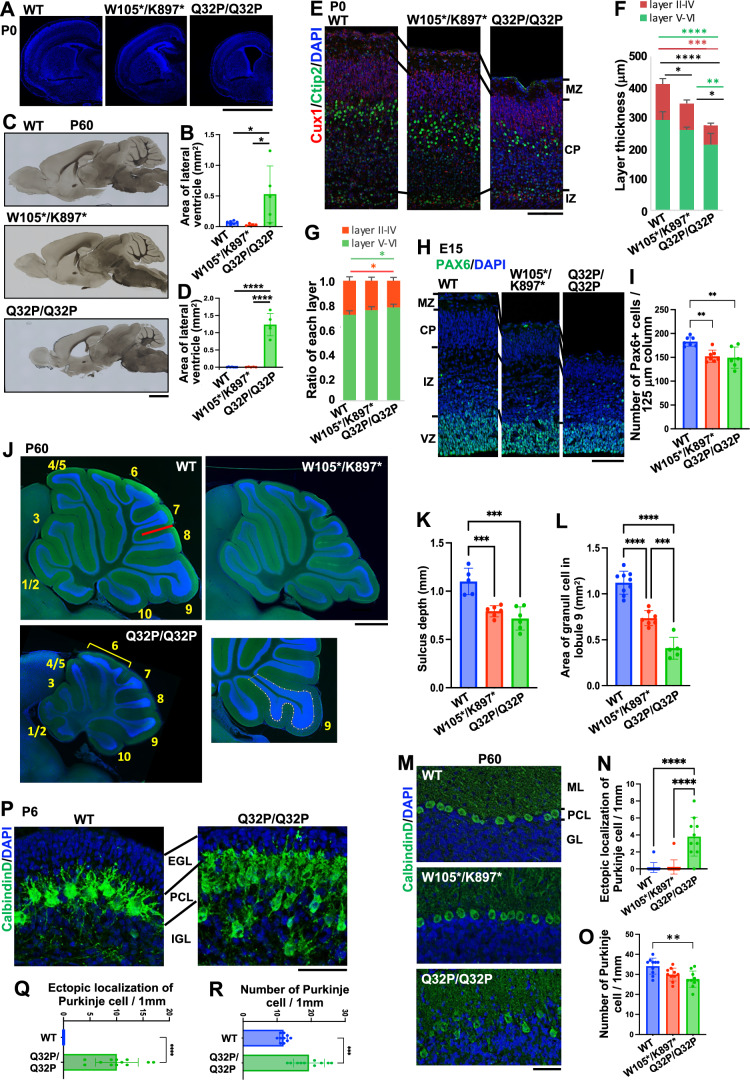
Impaired development of the cerebral cortex and cerebellum in Cep152^W105*/K897*^ and Cep152^Q32P/Q32P^ mice. (**A**) Coronal sections at P0 of the indicated genotypes were stained with DAPI. (**B**) Quantification of the lateral ventricle area shown in (**A**). Data were presented as mean ± SD. Sample sizes: *n* = 6, 4, and 5 for WT, Cep152^W105*/K897*^, and Cep152^Q32P/Q32P^, respectively. Tukey–Kramer LSD, *p = 0.0370 (WT vs Cep152^Q32P/Q32P^), *p = 0.0423 (Cep152^W105*/K897*^ vs Cep152^Q32P/Q32P^). (**C**) Whole sagittal sections spanning from the olfactory bulb to the pons from formalin-fixed brains at P60. (**D**) Quantification of lateral ventricle area from (**C**). Data were presented as mean ± SD. *n* = 7, 5, and 5 for WT, Cep152^W105*/K897*^, and Cep152^Q32P/Q32P^, respectively. Tukey–Kramer LSD, ****p < 0.0001. (**E**) Representative images of coronal sections of cerebral cortices (P0) stained for Ctip2 (green) and Cux1 (red). Nuclei were visualized with DAPI (blue). Cortical layers are labeled as follows: MZ marginal zone, CP cortical plate, IZ intermediate zone. (**F**, **G**) Quantification of layer thickness in (**E**). Data were presented as mean ± SD. (**F**) *n* = 7, 4, and 7 for WT, Cep152^W105*/K897*^, and Cep152^Q32P/Q32P^ mice, respectively. Black asterisks indicate comparisons of total cortical thickness among the three genotypes. Green and brown asterisks indicate comparisons of the thickness of layers V–VI and II–IV, respectively. Tukey–Kramer LSD, *p = 0.0437 (WT vs Cep152^W105*/K897*^), *p = 0.0197 (Cep152^W105*/K897*^ vs Cep152^Q32P/Q32P^), **p = 0.0091, ***p = 0.0006. (**G**) Green and red asterisks indicate comparisons of the ratios of layers V–VI and II–IV in (**E**), respectively. Tukey–Kramer LSD, *p = 0.0121 (layer II–IV), *p = 0.0121 (layer V–VI). (**H**) Representative images of cortical sections from WT, Cep152^W105*/K897*^, and Cep152^Q32P/Q32P^ mice (E15.5) stained with anti-Pax6 (green) and DAPI (blue). VZ ventricular zone. (**I**) Quantification of Pax6-positive cells in the sections shown in (**H**). Data are presented as mean ± SD. *n* = 7, 7, and 6 for WT, Cep152^W105*/K897*^, and Cep152^Q32P/Q32P^, respectively. Tukey–Kramer LSD, **p = 0.0044 (WT vs Cep152^W105*/K897*^), **p = 0.0029 WT vs Cep152^Q32P/Q32P^. (**J**) Gross morphological analyses of the cerebellum at P60. Midline sagittal sections from WT, Cep152^W105*/K897*^, and Cep152^Q32P/Q32P^ mice were stained for parvalbumin (green). Nuclei were counterstained with DAPI (blue). Each lobule is labeled in WT and Cep152^Q32P/Q32P^ panels. The sulcus depth between lobule 7 and lobule 8 is indicated by a red line, while the granule cell layer area of lobule 9 is marked by a dotted line. (**K**, **L**) Quantification of (**J**): sulcus depth between lobules 7 and 8 (**K**), Tukey–Kramer LSD, ***p = 0.0009 (WT vs Cep152^W105*/K897*^), ***p = 0.0001 (WT vs Cep152^Q32P/Q32P^), and granule cell layer area of lobule 9 (**L**), Tukey–Kramer LSD, ***p = 0.0003. Data were presented as mean ± SD. *n* = 5, 6, 6 in (**K**) and 9, 7, 5 in (**L**) for WT, Cep152^W105*/K897*^, and Cep152^Q32P/Q32P^, respectively. (**M**) Sagittal sections of cerebella from WT, Cep152^W105*/K897*^, and Cep152^Q32P/Q32P^ mice at P60 stained with anti-CalbindinD (green) and DAPI (blue). (**N**, **O**) Quantification of (**M**). Data were presented as mean ± SD. (**N**) Ectopically located Purkinje cells were counted in sagittal sections. *n* = 3 animals per genotype; 12, 13, and 10 fields of view for WT, Cep152^W105*/K897*^, and Cep152^Q32P/Q32P^, respectively. Tukey–Kramer LSD, ****p < 0.0001. (**O**) Quantification of Purkinje cell number. Tukey–Kramer LSD, **p = 0.0019. (**P**) Sagittal sections of cerebella from WT and Cep152^Q32P/Q32P^ mice at P6 stained with anti-CalbindinD (green) and DAPI (blue). (**Q**, **R**) Quantification of (**P**). Data were presented as mean ± SD. (**Q**) Ectopically located Purkinje cells. *n* = 3 animals per genotype; 14 and 12 fields of view for WT and Cep152^Q32P/Q32P^, respectively. Welch’s *t*-test, ****p < 0.0001. (**R**) Quantification of Purkinje cell number. *n* = 3 animals per genotype; 10 fields of view for each genotype. Welch’s *t*-test, ***p = 0.0004. Scale bars: 2 mm (**A**, **C**), 1 mm (**J**), 100 μm (**E**, **H**, **M**, **P**). Source data are available online for this figure.

**Figure EV4:**
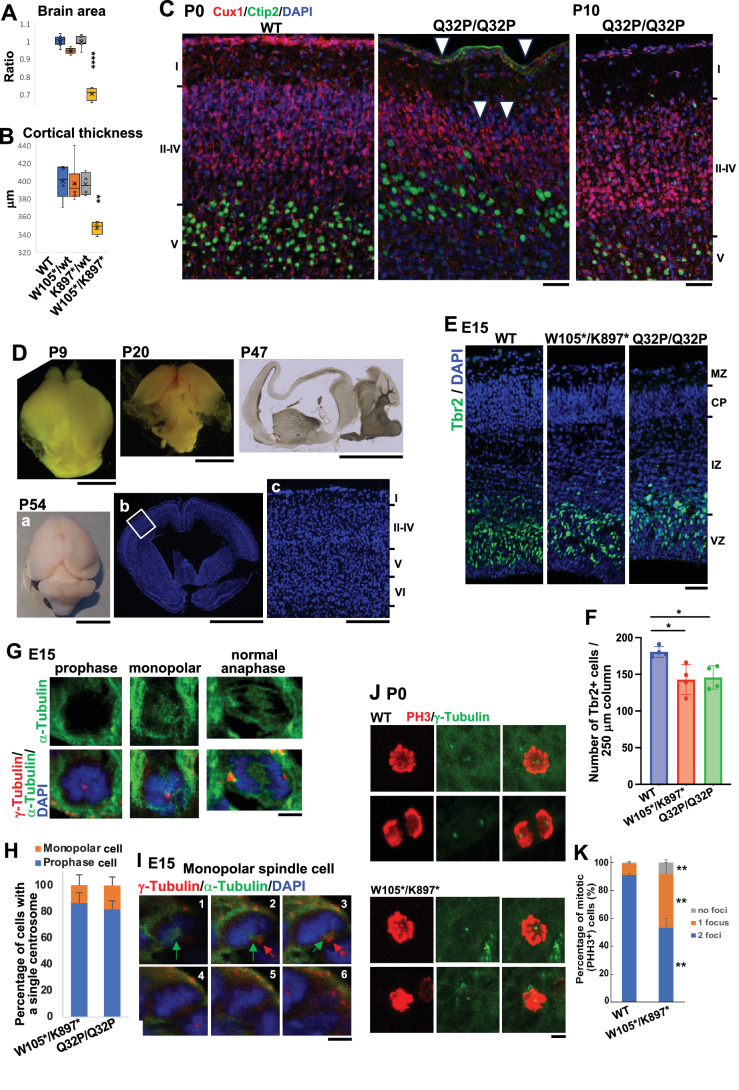
Gross and histological analyses of brain morphology in Cep152^W105*/K897*^ and Cep152^Q32P/Q32P^ mice. (**A**) Quantification of brain area in WT, Cep152^W105*/wt^, Cep152^K897*/wt^, and Cep152^W105*/K897*^ mice at P0. Values are shown as ratios relative to WT. Tukey–Kramer LSD, ****p < 0.0001. (**B**) Cortical thickness measured in the same cohort at P0. Sample sizes: (**A**) *n* = 12, 5, 7, 4; (**B**) *n* = 6, 4, 6, 4 for WT, Cep152^W105*/wt^, Cep152^K897*/wt^, and Cep152^W105*/K897*^, respectively. Tukey–Kramer LSD, **p = 0.0011. Box and whisker plots show the median (horizontal line), the 25th and 75th percentiles (box boundaries), and the whiskers extend to the largest and smallest values that are not outliers. A cross inside the boxes indicates the mean. (**C**) Representative images of cortical layers in WT (P0) and Cep152^Q32P/Q32P^ (P0 and P10), immunostained for Cux1 (upper-layer neurons), Ctip2 (deep-layer neurons), and nuclei (DAPI). Defects observed in the Cep152^Q32P/Q32P^ section were indicated by arrowheads. Note that the anti-Cux1 antibody also detects a Golgi-associated Cux1 isoform that is predominantly present during early postnatal stages. (**D**) Gross appearance of brains from Cep152^Q32P/Q32P^ mice at P9, P20, and P54, imaged using a fluorescent stereomicroscope, and a vibratome sagittal section at P47. A coronal section of the P54 brain (a) was stained with DAPI (b), and the boxed area in (b) is magnified to illustrate cortical layer organization (c). Note that the yellow coloration observed in the P9 and P20 images results from the characteristics of the fluorescent stereomicroscope filter and does not represent intrinsic tissue color. (**E**) Representative images of cortical sections from WT, Cep152^W105*/K897*^, and Cep152^Q32P/Q32P^ mice at E15.5 stained with anti-Tbr2 (a basal progenitor marker) and DAPI. (**F**) Quantification of (**E**). The number of Tbr2-positive cells per section was counted. *n* = 4 animals per genotype. Tukey–Kramer LSD, *p = 0.0188 (WT vs Cep152^W105*/K897*^), *p = 0.0286 (WT vs Cep152^Q32P/Q32P^). (**G**) Immunostaining of cerebral cortices from Cep152^Q32P/Q32P^ mice at E15.5 using anti-α-tubulin (spindle; green) and anti-γ-tubulin (centrosome; red). (**H**) Quantification of (**G**). Percentages of mitotic cells with a single centrosome in prophase or meta/anaphase were quantified. *n* = 5 and 4 for Cep152^W105*/K897*^ and Cep152^Q32P/Q32P^, respectively. Cell counts: Cep152^W105*/K897*^, 35; Cep152^Q32P/Q32P^, 35. (**I**) Sequential images of a monopolar mitotic cell in the VZ of a Cep152^Q32P/Q32P^ mouse at E15. Green and red arrows indicate spindle fibers and the centrosome, respectively. (**J**) Representative images of mitotic glial cells in the cortical plate at P0. Slices from WT and Cep152^W105*/K897*^ mice were stained for PH3 (red) and γ-tubulin (green) as shown in Fig. 4A. (**K**) Quantification of γ-tubulin foci in mitotic cells shown in (**J**). The foci were scored in mitotic glial cells. *n* = 4 animals per genotype. Cell counts: WT, 151; Cep152^W105*/K897*^, 200. Data were presented as mean ± SD for each animal. Scale bars: 5 mm (**D**; P9–P47 and P54, panels a and b), 500 µm (**D**; P54, panel c), 50 µm (**C**, **E**), 5 µm (**G**, **I**, **J**). Welch’s *t*-test, 2 foci (**p = 0.0013), 1 focus (**p = 0.0064), no foci (**p = 0.0058).

Gross morphological analysis at P0 and P60 confirmed a significant reduction in brain volume and enlargement of the lateral ventricles in Cep152^Q32P/Q32P^ mice (Fig. 3A–D). Phenotypes of Cep152^Q32P/Q32P^ brains at 6 months were also shown (Fig. EV3B,C). Since the enlargement observed may result from impaired cerebrospinal fluid circulation due to defects in motile cilia on the surface of ependymal cells, we immunostained ependymal cells with anti–γ-tubulin and quantified the number of motile cilia. As a result, the number was reduced in both mouse models, with a more prominent phenotype in Cep152^Q32P/Q32P^ mice (Fig. EV3D,E). This more pronounced loss of motile cilia in Cep152^Q32P/Q32P^ mice may lead to severely impaired cerebrospinal fluid circulation and, consequently, ventricular enlargement, as observed in both Cep152^Q32P/Q32P^ mice and the patients (Figs. 1C and 3A–D). Hypoplasia of the corpus callosum was detected in both models, with a more severity in Cep152^Q32P/Q32P^ mice (Fig. EV3F), recapitulating the phenotypes of Patient #2 and #3 (Figs. 1C, arrow and EV1F). When cortical thickness and layer structure were examined at P0 using immunostaining for Ctip2 (a deep cortical layer marker) and Cux1 (a superficial layer marker), both models exhibited reduced cortical thickness, with a more severe reduction observed in Cep152^Q32P/Q32P^ mice (Fig. 3E,F). This reduction was more pronounced in the superficial layer compared to the deep layer (Fig. 3G). This may be attributed to the fact that later-born neurons are more affected by the variants. Heterozygous Cep152^W105*/wt^ and Cep152^K897*/wt^ mice showed no significant differences in brain size compared with wild-type mice at P0, consistent with the recessive mode of inheritance (Fig. EV4A,B). Notably, the overall cortical layer structure was preserved in Cep152^W105*/K897*^ mice at P0 (Fig. 3E). In contrast, Cep152^Q32P/Q32P^ mice frequently exhibited disrupted cortical layer structure at this timing, accompanied by abnormal depressions (localized indentations) on the cortical surface (Figs. 3E and EV4C), resembling shallow grooves or dimples, which are no longer detectable by P10 (Fig. EV4C). This phenotype was observed in 56% of cases (*n* = 5/9 brains). It is noteworthy that prominent cerebral and cerebellar agenesis, as well as hydrocephalus, were frequently observed in Cep152^Q32P/Q32P^ mice (12.2%, *n* = 5/41 brains; Fig. EV4D). We then focused on the proliferation of radial glia and basal progenitor cells. Immunostaining for Pax6 (a radial glia marker) or Tbr2 (a basal progenitor marker) revealed reductions in both the thickness of the VZ (Fig. 3H,I) and the number of basal progenitor cells in both mutant models at E15.5 (Fig. EV4E,F), with no difference observed between Cep152^W105*/K897*^ and Cep152^Q32P/Q32P^ mice. These results indicate a similar level of neural progenitor reduction in these mice.

Similar to the cerebral cortex, the cerebella of Cep152^W105*/K897*^ and Cep152^Q32P/Q32P^ mice were found to be smaller in size at P60 (Fig. 3J). Significant morphological differences were observed, with greater severity in Cep152^Q32P/Q32P^ mice compared to wild-type littermates (Fig. 3J). Statistical analyses revealed a significant reduction in sulcus depth between lobules 7/8 (Fig. 3K) and in the area of the granule cell layer (GCL) in lobule 9 (Fig. 3L). Notably, the cerebellar layer structure was disrupted in Cep152^Q32P/Q32P^ mice, with an impaired localization and reduced number of Purkinje cells at P60 (Fig. 3M–O). To examine whether the abnormal organization of Purkinje cells in Cep152^Q32P/Q32P^ mice resulted from defective migration during development, we analyzed cerebellar architecture at P6, a developmental stage when Purkinje cells normally settle into their final positions. Notably, abnormal Purkinje cell arrangement was already evident at this stage (Fig. 3P,Q), suggesting that the observed defects are attributable not only to dysplasia but also to impaired migration. Furthermore, dendritic development of Purkinje cells appeared to be impaired in Cep152^Q32P/Q32P^ mice at this stage (Fig. 3P) while the number of immature Purkinje cells was paradoxically increased (Fig. 3R). However, the number of Purkinje cells was subsequently reduced at later stages (P60) (Fig. 3O), possibly as a result of apoptosis.

### Underlying mechanisms for defective brain development in Cep152^W105*/K897*^ and Cep152^Q32P/Q32P^ mice

**Figure 4:**
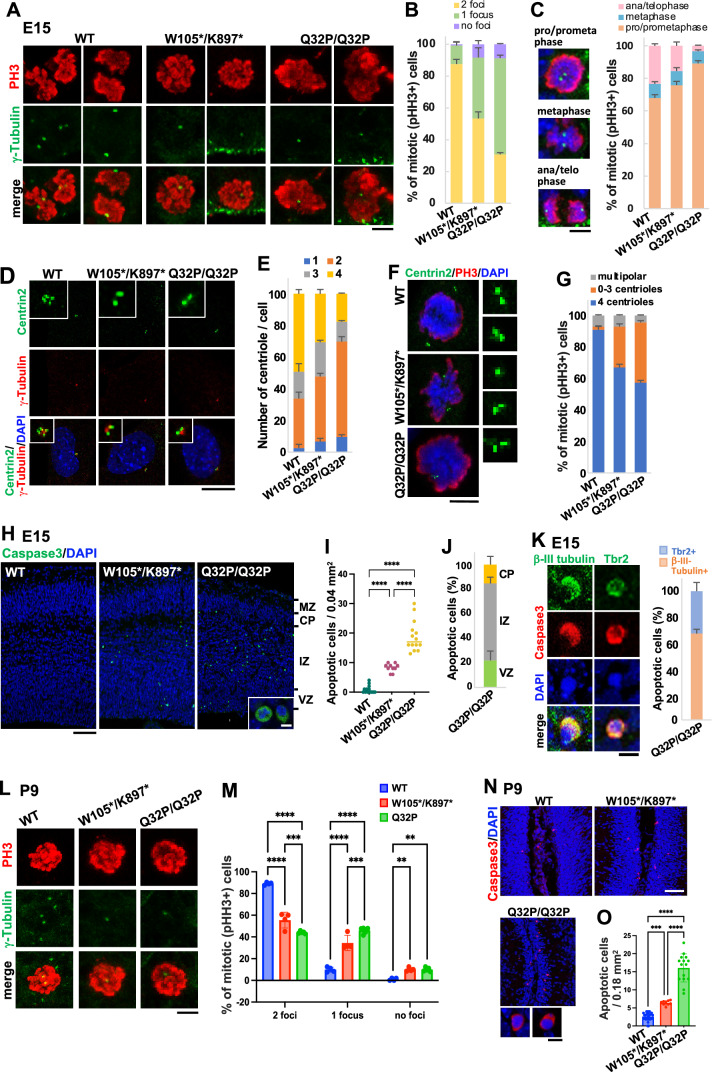
Analyses of mitotic spindle organization and apoptosis in cortical and cerebellar progenitors of Cep152^W105*/K897*^ and Cep152^Q32P/Q32P^ mice. (**A**) Representative images of mitotic cells in the VZ at E15.5. Cortical slices were double-immunostained for phospho-histone H3 (PH3, red) and γ-tubulin (green). Merged images are shown. (**B**) Quantification of (**A**). Number of γ-tubulin foci (centrosome) in mitotic cells. Data were presented as mean ± SD. *n* = 5 animals per genotype. Cell counts: WT, 129; Cep152^W105*/K897*^, 120; Cep152^Q32P/Q32P^, 104. (**C**) Scoring of mitotic configurations (pro/prometaphase, metaphase, and ana/telophase) for each genotype at E15.5. Representative images show PH3 (red), γ-tubulin (green), and DAPI (blue) staining (left). Data were presented as mean ± SD. *n* = 5 animals per genotype. Cell counts: WT, 150; Cep152^W105*/K897*^, 174; Cep152^Q32P/Q32P^, 121. (**D**) Representative images of MEFs double-immunostained for γ-tubulin (red) and Centrin2 (green). Nuclei were stained with DAPI (blue). Centrioles were magnified in the insets. Merged images are also shown. (**E**) Quantification of (**D**). The number of centrioles in MEFs. Data are presented as mean ± SD. *n* = 2 animals per genotype. Cell counts: WT, 322; Cep152^W105*/K897*^, 360; Cep152^Q32P/Q32P^, 325. (**F**) Representative images of MEFs stained for PH3 (red), Centrin2 (green), and nuclei (blue). Merged images are shown, with magnified views of Centrin2 staining. (**G**) Quantification of (**F**). The number of centrioles in mitotic cells. Data were presented as mean ± SD. *n* = 2 animals per genotype. Cell counts: WT, 100; Cep152^W105*/K897*^, 100; Cep152^Q32P/Q32P^, 115. (**H**) Representative images of cortical slices (E15.5) stained with anti-aCasp3 (green) and DAPI (blue). The insets show higher-magnification images. (**I**, **J**) Quantification of (**H**). (**I**) The number of aCasp3-positive cells per unit area (0.04 mm²) in the entire cerebral cortex. Data were presented as mean ± SD. *n* = 7, 5, 7 for WT, Cep152^W105*/K897*^, Cep152^Q32P/Q32P^, respectively. Tukey–Kramer LSD, ****p < 0.0001. (**J**) Distribution of apoptotic cells in Cep152^Q32P/Q32P^ cortices. Data were presented as mean ± SD. (*n* = 4). Total number of cells analyzed =  364. (**K**) Representative images of progenitors and neurons (E15.5) double-stained for aCasp3 (red) together with either Tbr2 or βIII-tubulin (green), respectively. Nuclei were stained with DAPI (blue). Merged images are also shown. The percentages of aCasp3/Tbr2 double-positive progenitor cells and aCasp3/βIII-tubulin double-positive neurons in the cortex were quantified. Data were presented as mean ± SD. *n* = 3 Cep152^Q32P/Q32P^ mice; total number of aCasp3-positive cells analyzed = 45. (**L**) Representative images of cerebellar mitotic cells (P9) stained for PH3 (red), γ-tubulin (green), and nuclei (DAPI, blue). (**M**) Quantification of (**L**) showing the number of γ-tubulin foci (centrosomes) per mitotic cell. Data were presented as mean ± SD. *n* = 4 animals per genotype. Cell counts: WT, 151; Cep152^W105*/K897*^, 160; Cep152^Q32P/Q32P^, 120. Tukey–Kramer LSD, 2 foci (***p = 0.0002, ****p < 0.0001), 1 focus (***p = 0.0001, ****p < 0.0001), no foci (**p = 0.0035, WT vs Cep152^W105*/K897*^; **p = 0.0023, WT vs Cep152^Q32P/Q32P^). (**N**) Representative images of cerebellar slices (P9) stained with anti-aCasp3 (green) and DAPI (blue). (**O**) Quantification of (**N**) showing the number of apoptotic cells per unit area (0.18 mm²) in the external and internal granule layers. Data were presented as mean ± SD. *n* = 8, 4, 5 for WT, Cep152^W105*/K897*^, Cep152^Q32P/Q32P^, respectively. Fields of view: 26, 9, 15 for WT, Cep152^W105*/K897*^, Cep152^Q32P/Q32P^, respectively. Tukey–Kramer LSD, ***p = 0.0003, ****p < 0.0001. Scale bars: 5 μm (**A**, **C**, **F**, **H** right, **K**, **L**, **N** lower), 10 μm (**D**), and 50 μm (**N** upper), 100 μm (**H** left). Source data are available online for this figure.

To investigate the mechanisms underlying cerebral developmental defects in Cep152^W105*/K897*^ and Cep152^Q32P/Q32P^ mice, we examined the mitotic features and spindle pole configurations in VZ progenitors or MEFs. When double immunostaining was performed for phospho-histone H3 (PH3) and γ-tubulin (a PCM marker) in the VZ, control mitotic cells exhibited two distinct γ-tubulin foci, whereas both mutant models showed a reduced number of foci, with the defects being more pronounced in Cep152^Q32P/Q32P^ mice (Fig. 4A,B). Quantification of the proportions of cells with two foci in different mitotic phases (pro/prometaphase, metaphase, and ana/telophase) revealed that both mutant models displayed an increased proportion of cells in pro/prometaphase at E15.5, accompanied by a reduction in cells in ana/telophase, with a more pronounced delay in Cep152^Q32P/Q32P^ mice (Fig. 4C). To determine whether cells displaying an abnormal single γ-tubulin focus were in the early (prophase) or later (meta/anaphase) stages of mitosis, we performed double immunostaining with anti–γ-tubulin and anti–α-tubulin. This analysis revealed that among mitotic cells exhibiting a single γ-tubulin focus, monopolar spindles were observed in 13% of cells in Cep152^W105*/K897*^ mice and 18% in Cep152^Q32P/Q32P^ mice; the majority of these cells were in prophase (Fig. EV4G,H). Serial images of a monopolar spindle were also shown (Fig. EV4I). We next assessed centrosomal structure by focusing on the centrioles. To this end, we prepared MEFs from control and mutant embryos and performed double immunostaining for Centrin2 (a centriole marker) and γ-tubulin. Both mutant cell types displayed a reduced number of centrioles, with the defects being more pronounced in Cep152^Q32P/Q32P^ mice (Fig. 4D,E). We further examined mitotic abnormalities in these MEFs by visualizing PH3 and Centrin2. As a result, mutant MEFs again exhibited reduced centriole numbers, with a more prominent phenotype observed in Cep152^Q32P/Q32P^ cells (Fig. 4F,G).

Then, to determine whether these variants also affect glial cells generation, we examined the cerebral cortices of Cep152^W105*/K897*^ mice at P0. Immunostaining with anti-γ-tubulin and anti- PH3 revealed a significant decrease in bipolar cells, along with a concomitant increase in monopolar and non-polar dividing cells in both models (Fig. EV4J,K).

Taken together, these results demonstrate that neural progenitors in both Cep152^W105*/K897*^ and Cep152^Q32P/Q32P^ mice, as well as MEFs derived from these mice, display defects in centriole duplication, as evidenced by an increased frequency of monopolar spindles and abnormal centriole numbers (1–3 instead of the normal 4 during mitosis). These abnormalities lead to defective spindle pole assembly and mitotic delay, ultimately compromising cell viability (Salisbury et al, 2002).

In line with the observed mitotic delays, we detected a significant increase in apoptosis, as indicated by the number of activated caspase3 (aCasp3)-positive cells. This effect was more pronounced in Cep152^Q32P/Q32P^ mice (Fig. 4H,I). Although apoptotic cells were distributed throughout the cortex, the highest prevalence was observed in the IZ (Fig. 4H,J). To further investigate the cell types undergoing apoptosis, we performed double immunostaining on E15.5 cortical sections from Cep152^Q32P/Q32P^ mice using anti-aCasp3 together with either anti-Tbr2 or anti-βIII-tubulin (a neuronal marker). Apoptotic cells were present in both populations, but were more frequently observed among neurons (Fig. 4K).

We next investigated the mechanisms underlying the cerebellar developmental defects in these models. Granule cells from P9 brains were examined using double-staining for PH3 and γ-tubulin. In wild-type sections, most PH3-positive mitotic granule cells in the external granular layer (EGL) displayed two γ-tubulin foci (Fig. 4L,M). In contrast, in the EGL of both mouse models, a significantly higher proportion of cells exhibited no foci or one focus, with the phenotype being more pronounced in Cep152^Q32P/Q32P^ (Fig. 4L,M). These defects likely contribute to mitotic delay and altered cell viability. Immunostaining using anti-aCasp3 revealed a two-fold and five-fold increase in apoptotic cells in the EGL of Cep152^W105*/K897*^ and Cep152^Q32P/Q32P^ brains (P9), respectively, compared to wild-type littermates (Fig. 4N,O). Since granule cell precursors in the EGL actively proliferate before migrating inward to the internal granular layer (IGL) and differentiating into mature granule cells (Butts et al, 2014; Marzban et al, 2015), increased apoptosis in the EGL may reduce the number of granule cell precursors, contributing to cerebellar hypoplasia. Consistent with cortical findings, cerebellar phenotypes were more prominent in Cep152^Q32P/Q32P^ mice than in Cep152^W105*/K897*^ mice.

**Figure EV5:**
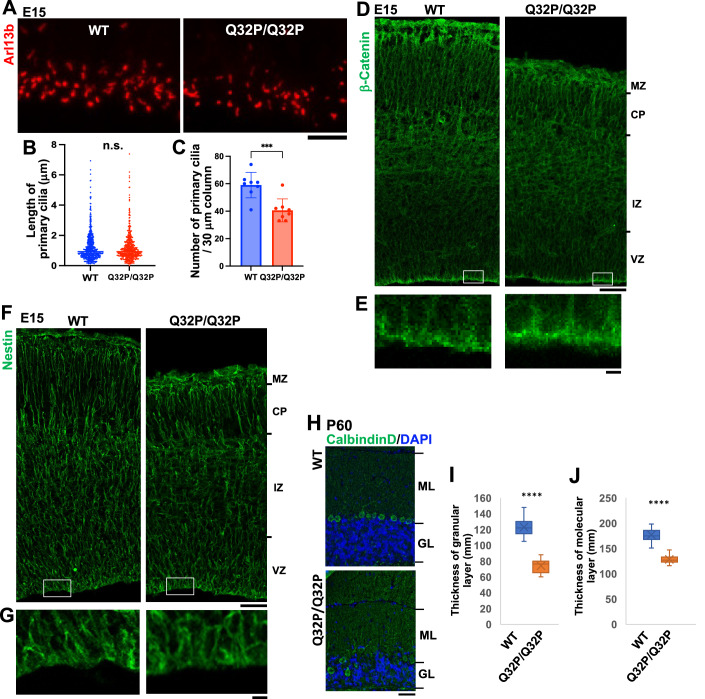
Additional histological analyses of brain structure in Cep152^Q32P/Q32P^ mice. (**A**) Representative images of primary cilia in VZ progenitor cells at E15.5. Cortical slices were stained with anti-Arl13b (red). (**B**, **C**) Quantification of (**A**). (**B**) Length of primary cilia were measured. *n* = 8 and 6 for WT and Cep152^Q32P/Q32P^, respectively. Cell counts: WT, 598; Cep152^Q32P/Q32P^, 556. (**C**) The number of primary cilia per 30 μm column was counted. *n* = 4 animals per genotype; 8 fields of view for WT and Cep152^Q32P/Q32P^, respectively. Welch’s *t*-test, ***p = 0.0010. (**D**) Representative images of cortical slices (E15.5) stained with anti-β-catenin (an adherens junction marker). (**E**) Magnified images from squares in (**D**). (**F**) Representative images of cortical slices (E15.5) stained with anti-nestin (a radial glia marker). (**G**) Magnified images from squares in (**F**). (**H**) Representative images of cerebellar cortex sections in the lobule 4/5 from WT and Cep152^Q32P/Q32P^ mice at P60 stained with anti-CalbindinD (green) and DAPI (blue). ML molecular layer, GL granular layer. (**I**, **J**) Quantification of (**H**): thickness of the granular layer (**I**) and molecular layer (**J**). Box and whisker plots show the median (horizontal line), the 25th and 75th percentiles (box boundaries), and the whiskers extend to the largest and smallest values that are not outliers. A cross inside the boxes indicates the mean. Data were presented as mean ± SD. *n* = 10 and 8 for WT and Cep152^Q32P/Q32P^. Welch’s *t*-test, ****p < 0.0001. Scale bars: 10 μm (**A**), 50 μm (**D**, **F**, J), 5 μm (**E**, **G**).

Centriole loss following depletion of CPAP, another centriole protein, has been reported to cause primary cilium loss, detachment of radial glial cells, and extensive heterotopia due to disruption of neuronal junctions in conditional null mice (Insolera et al, 2014). To determine whether the drastic tissue disorganization observed in Cep152^Q32P/Q32P^ mice results from similar junctional defects, we examined the morphology of primary cilia in VZ progenitor cells by immunostaining with anti-Arl13b. Notably, the primary cilia appeared morphologically normal in Cep152^Q32P/Q32P^ mice, whereas their number was reduced, likely due to a decrease in the population of Pax6-positive apical progenitors (Fig. EV5A–C). The localization of β-catenin, a junctional marker, in the VZ of mutant brains was comparable to that in wild-type brains (Fig. EV5D,E). The morphology of radial glial fibers also appeared preserved (Fig. EV5F,G). Collectively, these findings suggest that the molecular mechanism of *CEP152*-related disorder differs from that of *CPAP* gene abnormalities.

**Figure 5:**
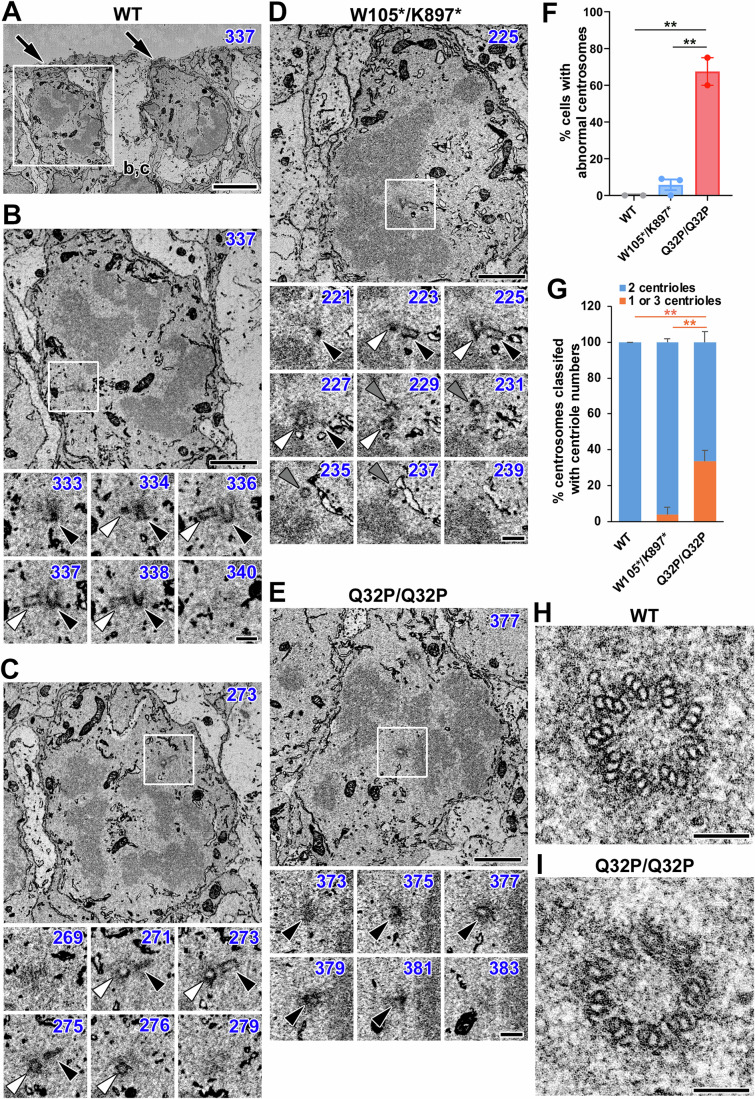
Ultrastructural abnormalities of centrosomes in Cep152^W105*/K897*^ and Cep152^Q32P/Q32P^ mice. (**A**–**C**) Representative serial block-face scanning electron microscopy images of mitotic cells near the ventricular surface of E15.5 WT fetal brains. In WT cells (**A**), two centrosomes are observed in serial sections, each containing a pair of centrioles (**B**, **C**; black and white arrowheads). White square in (**A**) indicates the regions shown in higher-magnification serial images in (**B**, **C**). (**D**, **E**) Representative serial images from Cep152^W105*/K897*^ (**D**) and Cep152^Q32P/Q32P^ (**E**) mutant brains. Abnormal centrosomes containing three centrioles (**D**; black, white, and gray arrowheads) or a single centriole (**E**; black arrowheads) are observed. White squares indicate regions shown at higher magnification. (**F**, **G**) Quantification of centrosomal abnormalities. Data were presented as mean ± SD. *n* = 2, 3, 2 for WT, Cep152^W105*/K897*^, Cep152^Q32P/Q32P^ mice. (**F**) Percentage of mitotic cells containing abnormal centrosomes (each dot represents one animal). Total centrosomes counted: 19, 71, 27 for WT, Cep152^W105*/K897*^, Cep152^Q32P/Q32P^, respectively. Tukey–Kramer LSD, **p = 0.0010 (WT vs Cep152^Q32P/Q32P^), **p = 0.0010 (Cep152^W105*/K897*^ vs Cep152^Q32P/Q32P^). (**G**) Proportion of centrosomes with different centriole numbers in WT, Cep152^W105*/K897*^, and Cep152^Q32P/Q32P^ brains. Total centrosomes counted: 38, 140, 53 for WT, Cep152^W105*/K897*^, Cep152^Q32P/Q32P^, respectively. Orange asterisks indicate comparison of 1 or 3 centrioles in WT, Cep152^W105*/K897*^, and Cep152^Q32P/Q32P^ brains. Tukey–Kramer LSD, **p = 0.0037 (WT vs Cep152^Q32P/Q32P^), **p = 0.0046 (Cep152^W105*/K897*^ vs Cep152^Q32P/Q32P^). (**H**, **I**) TEM images showing the ultrastructure of a centriole in a mitotic WT cell (**H**), which displays clear triplet microtubules, and an abnormal centriole in a Cep152^Q32P/Q32P^ cell (**I**), which exhibits disrupted microtubule arrangement. The number in the upper right corner of each panel indicates the serial slice number within the image stack. Scale bars: 5 μm (**A**), 2 μm (**B**, **D**, **E**), 500 nm (higher-magnification images in **B**, **D**, **E**), 100 nm (**H**, **I**). Source data are available online for this figure.

We further examined the ultrastructure of centrioles in VZ progenitor cells of Cep152^W105*/K897*^ and Cep152^Q32P/Q32P^ mice using serial block-face scanning electron microscopy. Hundreds of serial electron microscopic images containing mitotic (prophase and metaphase) cells were obtained from wild-type embryos at E15.5 (Fig. 5A). Serial image stacks capturing entire centrosomes showed that wild-type cells consistently possessed two centrosomes, each containing a pair of orthogonally arranged centrioles (Fig. 5A–C). In contrast, mutant cells exhibited centrosomes with abnormal centriole numbers, including triplet centrioles in Cep152^W105*/K897*^ mice (Fig. 5D) and predominantly singlet or triplet centrioles in Cep152^Q32P/Q32P^ mice (Fig. 5E). Quantification demonstrated that Cep152^Q32P/Q32P^ mice displayed a markedly higher proportion of mitotic cells with abnormal centrosomes, showing a statistically significant increase compared with wild-type (Fig. 5F). Consistent with this, the distribution of centriole numbers was significantly skewed toward 1 or 3 in Cep152^Q32P/Q32P^ mice (Fig. 5G). By contrast, although some Cep152^W105*/K897*^ cells exhibited abnormal centrosomes, the overall frequency did not differ significantly from wild-type, and the distribution of centriole numbers showed only a mild deviation (Fig. 5F,G). The frequency of mitotic cells with abnormal centriole numbers was higher in Cep152W105*/K897* MEFs than in apical progenitors analyzed by electron microscopy in vivo. This difference may reflect cell type-specific effects as well as methodological differences between immunostaining-based analysis in MEFs and EM-based analysis of apical progenitors. Furthermore, cross-sectional transmission electron microscopy (TEM) analyses revealed normal triplet microtubule organization in wild-type centrioles (Fig. 5H), whereas a subset of Cep152^Q32P/Q32P^ centrioles displayed disorganized microtubule structures (Fig. 5I). Such structural abnormalities were far less frequent in Cep152^W105*/K897*^ mice. Together, these findings indicate that centriole duplication and structural organization are severely disrupted in Cep152^Q32P/Q32P^ progenitors, whereas Cep152^W105*/K897*^ mice display only mild and non–statistically significant defects, consistent with the milder overall neurodevelopmental phenotype.

### CEP152 is crucial for PLK4 localization at the centrosome

**Figure 6:**
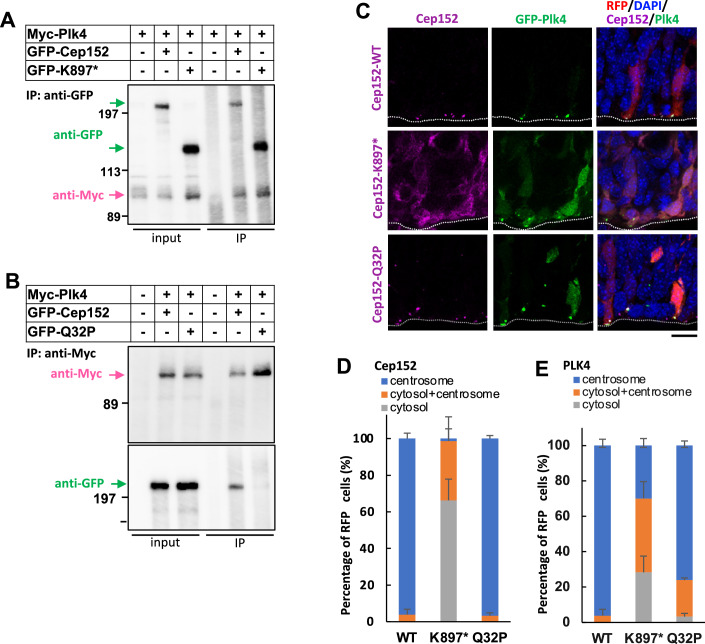
Localization PLK4 is differently influenced by CEP152-K897* and CEP152-Q32P. (**A**) Interaction of CEP152-K897* with PLK4. COS7 cells were transfected with pCAG-GFP-CEP152, CEP152-K897*, and pCAG-Myc-PLK4 (1 µg each) in various combinations as indicated. Cell lysates were immunoprecipitated using a polyclonal anti-GFP. Twenty percent of the immunoprecipitated material was analyzed by Western blotting (7.5% gel) and using polyclonal anti-GFP and anti-Myc (IP). Protein expression in cell lysates (input; 3% of total volume) was similarly confirmed with these antibodies. (**B**) Interaction of CEP152-Q32P with PLK4. COS7 cells were transfected with pCAG-GFP-CEP152, -CEP152-Q32P, and pCAG-Myc-PLK4 (1 µg each) in various combinations as indicated. Cell lysates were immunoprecipitated with a polyclonal anti-Myc. Western blotting was performed as described in (A). (**C**) Effects of CEP152-K897* and CEP152-Q32P on the subcellular distribution of PLK4 in vivo. pCAG-RFP (0.3 µg) was co-electroporated in utero with pCAG-GFP-PLK4 (0.5 µg) and either pCAG-Myc-CEP152 (WT), -CEP152-K897*, or -CEP152-Q32P (0.5 µg each) into VZ cells at E13.5. Coronal brain sections were prepared at E15.5 and stained with anti-GFP (green) and anti-Myc (magenta). Nuclei were stained with DAPI (blue). Representative images of VZ cells expressing CEP152 (*upper* panels), CEP152-K897* (*middle* panels), and CEP152-Q32P (*lower* panels) are shown. Scale bar, 10 μm. (**D**, **E**) Quantification of CEP152 (**D**) and PLK4 (**E**) localization in the cells from (**C**). Data were presented as mean ± SD. *n* = 5 animals per genotype. Cells scored (*n*): in (**D**), WT (156), K897* (302), and Q32P (118); in (**E**), WT (156), K897* (302), and Q32P (121). Source data are available online for this figure.

PLK4, a serine/threonine protein kinase, is crucial for centriole duplication and mitotic progression (Fırat-Karalar and Stearns, 2014; Arquint and Nigg, 2016; Park et al, 2019). Notably, PLK4 has been implicated in microcephaly and MPD (Tsutsumi et al, 2016; Martín-Rivada et al, 2021; Dinçer et al, 2017; Shaheen et al, 2014). CEP152 binds PLK4 and acts as a scaffold to facilitate its recruitment to the centrosome (Cizmecioglu et al, 2010; Hatch et al, 2010). To investigate the impact of *CEP152* variants on this interaction, we performed immunoprecipitation assays to examine the binding of PLK4 with CEP152-K897*, which retains the PLK4-binding region, and with CEP152-Q32P, which carries a substitution within this critical region. Our analysis revealed that both wild-type CEP152 and CEP152-K897* co-precipitated with PLK4, whereas CEP152-Q32P failed to interact with PLK4 under identical conditions (Fig. 6A,B). These results suggest that the p.Q32P variant disrupts the CEP152–PLK4 interaction.

We next conducted in vivo experiments to evaluate the effects of CEP152-K897* and CEP152-Q32P on PLK4 localization in neural progenitors during corticogenesis. In utero electroporation was performed at E13.5 with pCAG-RFP and pCAG-GFP-PLK4, along with expression vectors for Myc-CEP152, -CEP152-K897*, or -CEP152-Q32P. Brain samples were collected at E15.5 and subjected to immunostaining for GFP, Myc, and nuclei. Consistent with results from transfected N2A cells (Fig. 2B), CEP152-K897* exhibited diffuse cytoplasmic localization, whereas wild-type CEP152 and CEP152-Q32P localized to the centrosome in electroporated cells (Fig. 6C,D). Notably, PLK4 localized correctly at the centrosome in the presence of wild-type CEP152 but was mislocalized in cells expressing either CEP152-K897* or CEP152-Q32P, with more pronounced disruption observed in the CEP152-K897* condition (Fig. 6C,E).

### Morphology of cortical excitatory neurons and Purkinje cells in Cep152^W105*/K897*^ and Cep152^Q32P/Q32P^ mice

**Figure 7:**
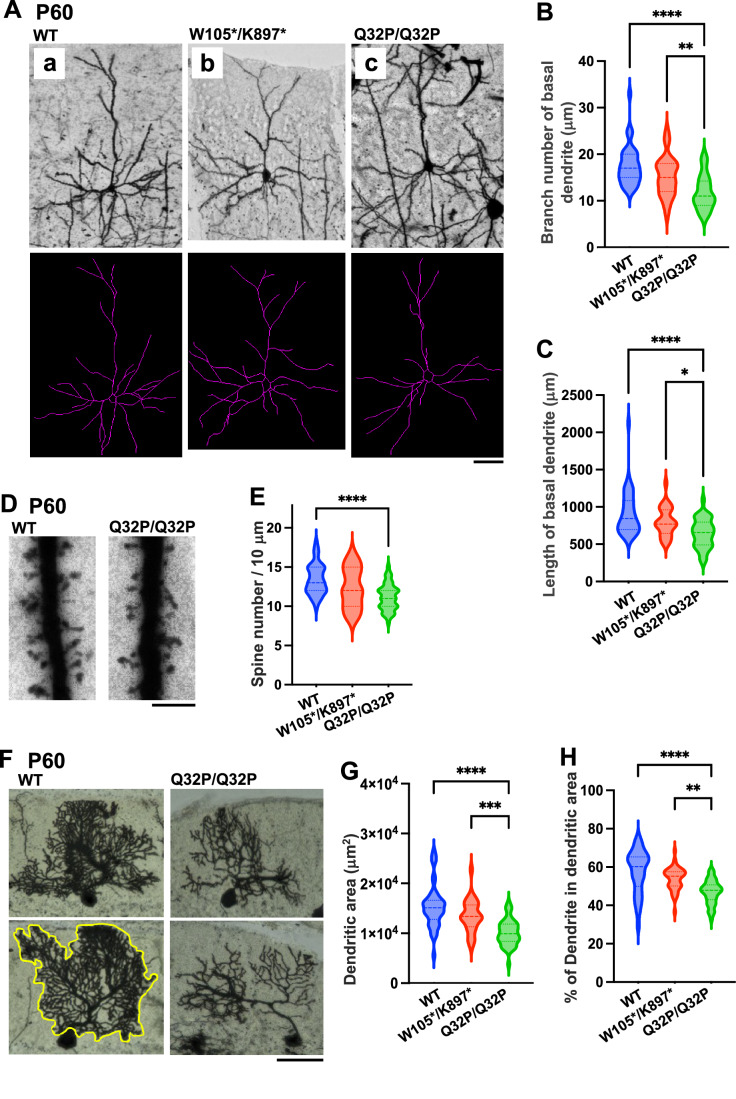
Morphological analyses of dendrites and spines of cortical neurons and Purkinje cells in Cep152^W105*/K897*^ and Cep152^Q32P/Q32P^ mice. (**A**) Representative images of Golgi–Cox-stained pyramidal neurons in the somatosensory cortex of WT, Cep152^W105*/K897*^, and Cep152^Q32P/Q32P^ mice at P60 (*upper* panels). Corresponding neuron tracings generated using ImageJ software are shown (lower panels). (**B**, **C**) Quantification of branch number (**B**) and length (**C**) of basal dendrites from (**A**). Analysis included four animals per genotype, with ten slices per animal. Neurons analyzed (n): WT (39), Cep152^W105*/K897*^ (30), Cep152^Q32P/Q32P^ (38). (**B**) Tukey–Kramer LSD, **p = 0.0020, ****p < 0.0001, (**C**); Tukey–Kramer LSD, *p = 0.0239, ****p < 0.0001. (**D**) Representative images of Golgi–Cox-stained spines from the apical dendrites of layer II/III pyramidal neurons in WT and Cep152^Q32P/Q32P^ mice at P60. (**E**) Quantification of spine density from (**D**), based on four animals per genotype and ten slices per animal. Neurons analyzed (n): WT (34), Cep152^W105*/K897*^ (30), Cep152^Q32P/Q32P^ (41). Tukey–Kramer LSD, ****p < 0.0001 (**F**) Representative images of Golgi–Cox-stained Purkinje cells from the cerebella of WT and Cep152^Q32P/Q32P^ mice at P60. The dendritic area is outlined in yellow. (**G**, **H**) Quantification of dendritic area (**G**) and the percentage of dendrites occupying the dendritic area (**H**) from (**F**), based on four animals per genotype. Analysis was based on ten slices per animal. Neurons analyzed (n): in (**G**), WT (45), Cep152^W105*/K897*^ (28), Cep152^Q32P/Q32P^ (43); in (**H**), WT (57), Cep152^W105*/K897*^ (44), Cep152^Q32P/Q32P^ (44). Tukey–Kramer LSD, **p = 0.0017, ***p = 0.0002, ****p < 0.0001. Scale bars: 50 μm (**A**, **F**), 5 μm (**D**). Source data are available online for this figure.

Given that ID is a primary neurological feature of MCPH, and that an MCPH3 mouse model carrying an inversion of exon 4 in *Cdk5rap2* exhibited abnormal cortical neuron morphology (Zaqout et al, 2019), we hypothesized that cortical neurons in our mouse models might also display morphological abnormalities at the cellular level, potentially impacting the formation and maintenance of synaptic networks (Zaqout et al, 2019). To test this, we analyzed the morphology of dendrites and synapses in Golgi–Cox-stained pyramidal neurons located in layer II/III of the somatosensory cortex of Cep152^W105*/K897*^ and Cep152^Q32P/Q32P^ mice at P60. In Cep152^W105*/K897*^ mice, basal dendrites of pyramidal neurons exhibited normal length and branching comparable to control littermates (Fig. 7A,a, b,B,C), and spine density was similarly unaffected (Fig. 7D,E), indicating preserved neuronal morphology and synaptic integrity. In contrast, Cep152^Q32P/Q32P^ mice showed significant reductions in both dendritic length and branching at P60, as well as decreased spine density (Fig. 7A,a, c,B–E), reflecting more pronounced impairments in neuronal and synaptic development.

We next examined Purkinje cell morphology, and observed significantly underdeveloped Purkinje cells in Cep152^Q32P/Q32P^ mice at P60, characterized by impaired dendritic arborization (Fig. 7F–H). The reduced granular and molecular layer thicknesses (Fig. EV5H–J) may result from a reduced number of granule cells, impaired dendritic development of Purkinje cells, reduced extension of parallel fibers from granule cells, or a combination of these factors. In contrast, Cep152^W105*/K897*^ mice showed no significant alterations in Purkinje cell dendritic development (Fig. 7G,H), suggesting that these variants have minimal effect on Purkinje cell morphology.

### Synaptic transmission and excitability in cortical neurons of Cep152^W105*/K897*^ and Cep152^Q32P/Q32P^ mice

**Figure 8:**
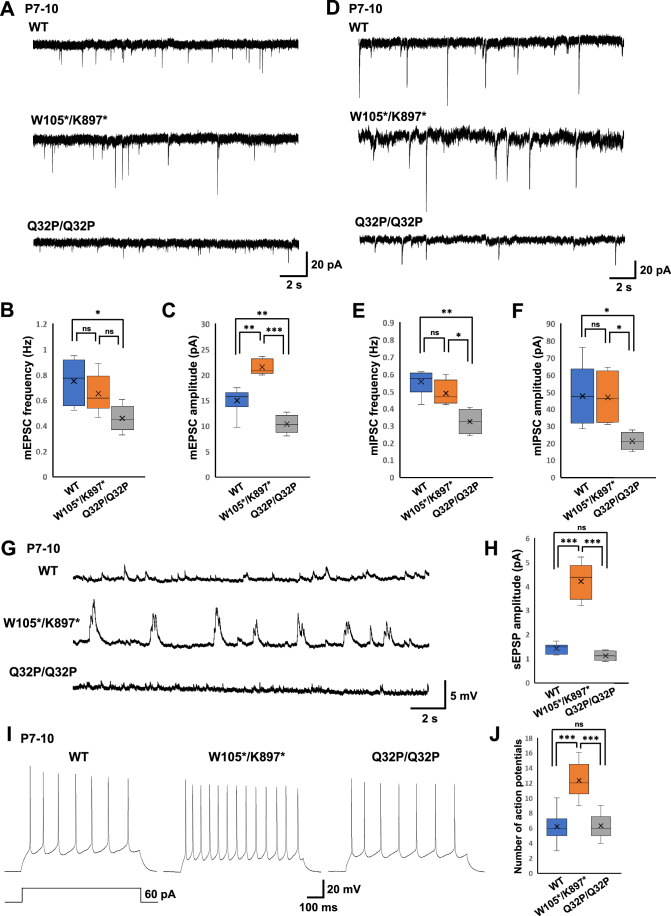
Electrophysiological analyses using whole-cell recordings of pyramidal neurons within layer Ⅱ/Ⅲ of the postnatal cerebral cortex. (**A**–**C**) Postsynaptic currents were recorded at a holding potential of −60 mV in WT, Cep152^W105*/K897*^, and Cep152^Q32P/Q32P^ mice at P7–10. Consecutive traces of mEPSCs were recorded in the presence of bicuculline (a GABA_A_ receptor antagonist, 10 μM), strychnine (a glycine receptor antagonist, 0.5 μM) and tetrodotoxin (TTX) (a sodium channel blocker, 0.5 μM) from wild-type (WT), Cep152^W105*/K897*^ and Cep152^Q32P/Q32P^ mice. Quantification of mEPSC frequency (**B**) and amplitude (**C**) is shown. Recordings were conducted for six WT and five Cep152^W105*/K897*^ neurons from three slices of two mice, and five Cep152^Q32P/Q32P^ neurons from four slices of three mice. Tukey–Kramer LSD, *p = 0.0191 (WT vs Cep152^Q32P/Q32P^) shown in (**B**) and **p = 0.0006 (WT vs Cep152^W105*/K897*^), **p = 0.0096 (WT vs Cep152^Q32P/Q32P^), ***p < 0.0001 (Cep152^W105*/K897*^ vs Cep152^Q32P/Q32P^) shown in (**C**). (**D**–**F**) Consecutive traces of mIPSCs were recorded in the presence of 6,7-Dinitroquinoxaline-2,3-dione (DNQX) (a non-NMDA receptor antagonist, 5 μM), D-AP5 (an NMDA receptor antagonist, 25 μM), strychnine (0.5 μM) and TTX (0.5 μM) in wild-type (WT), Cep152^W105*/K897*^, and Cep152^Q32P/Q32P^ mice at P7–10. Quantification of mIPSC frequency (**E**) and amplitude (**F**) are also shown. Five WT and 4 Cep152^Q32P/Q32P^ neurons were analyzed from three slices of two mice, while four neurons from two slices of two Cep152^W105*/K897*^ mice were also analyzed. Tukey–Kramer LSD, **p = 0.0027 (WT vs Cep152^Q32P/Q32P^), *p = 0.0282 (Cep152^W105*/K897*^ vs Cep152^Q32P/Q32P^) shown in (**E**) and *p = 0.0327 (WT vs Cep152^Q32P/Q32P^), *p = 0.0498 (Cep152^W105*/K897*^ vs Cep152^Q32P/Q32P^) shown in (**F**). (**G**, **H**) Consecutive traces of sEPSPs (**G**) and quantification of the amplitude (**H**). For WT and Cep152^W105*/K897*^ mice, six and five neurons, respectively, were analyzed from two slices per mouse, with two mice analyzed for each genotype. For Cep152^Q32P/Q32P^ mice, six neurons from three slices of three mice were analyzed. Tukey–Kramer LSD, ***p < 0.0001 (WT vs Cep152^W105*/K897*^), ***p < 0.0001 (Cep152^W105*/K897*^ vs Cep152^Q32P/Q32P^). (**I**, **J**) Action potentials evoked by a 60 pA current injection for 800 ms (**I**) and quantification of the number (**J**) in P7–10 mice. For WT mice, ten neurons were analyzed from five slices of three mice. For Cep152^W105*/K897*^ mice, six neurons were analyzed from three slices of two mice. For Cep152^Q32P/Q32P^ mice, nine neurons were analyzed from four slices of three mice. Tukey–Kramer LSD, ***p < 0.0001 (WT vs Cep152^W105*/K897*^), ***p < 0.0001 (Cep152^W105*/K897*^ vs Cep152^Q32P/Q32P^). Source data are available online for this figure.

To investigate the impact of Cep152^W105*/K897*^ and Cep152^Q32P/Q32P^ variants on synaptic function and neuronal excitability, we performed whole-cell patch-clamp recordings on layer II/III pyramidal neurons in cortices from P7–10 mice. Voltage-clamp recordings revealed that miniature excitatory postsynaptic current (mEPSC) frequencies were significantly reduced in Cep152^Q32P/Q32P^ neurons compared to wild-type, while Cep152^W105*/K897*^ neurons showed no significant difference (Fig. 8A,B). mEPSC amplitudes were significantly larger in Cep152^W105*/K897*^ neurons and lower in Cep152^Q32P/Q32P^ neurons compared to wild-type (Fig. 8A,C). In contrast, miniature inhibitory postsynaptic currents (mIPSCs) exhibited reduced frequencies and amplitudes in Cep152^Q32P/Q32P^ neurons, with no significant differences observed in Cep152^W105*/K897*^ neurons (Fig. 8D–F). Based on these findings, the p.W105*/p.K897* variants are likely to enhance excitatory synaptic transmission without affecting inhibitory transmission in layer Ⅱ/Ⅲ pyramidal neurons, thereby shifting the excitatory/inhibitory (E/I) balance toward excitation. In contrast, the p.Q32P variant impairs both excitatory and inhibitory synaptic transmission, likely due to a general disruption of synaptic function rather than a shift in E/I balance.

Current-clamp recordings showed no significant differences in resting membrane potentials among the wild-type, Cep152^W105*/K897*^, and Cep152^Q32P/Q32P^ neurons (−61.4 ± 1.43, −61.3 ± 2.16, and −61.5 ± 1.89 mV, respectively). Although spontaneous action potentials were absent in all genotypes, spontaneous excitatory postsynaptic potentials (sEPSPs) were observed. sEPSP amplitudes were significantly larger in Cep152^W105*/K897*^ neurons compared to wild-type and Cep152^Q32P/Q32P^ neurons (Fig. 8G,H). These findings indicate stronger excitatory input and depolarization in layer Ⅱ/Ⅲ pyramidal neurons of Cep152^W105*/K897*^ mice compared to those of Cep152^Q32P/Q32P^ mice.

To evaluate neuronal excitability, we elicited action potentials with 60 pA current pulses (800 ms). Cep152^W105*/K897*^ neurons displayed significantly higher firing rates compared to wild-type and Cep152^Q32P/Q32P^ neurons (Fig. 8I,J). These results suggest that the p.W105*/p.K897* variants enhance neuronal excitability by activating voltage-gated sodium and/or potassium channel functions, whereas the p.Q32P variant does not affect intrinsic excitability but instead impairs synaptic inputs.

### Significant differential expression of neuronal genes exclusively in Cep152^Q32P/Q32P^ mice

**Figure 9:**
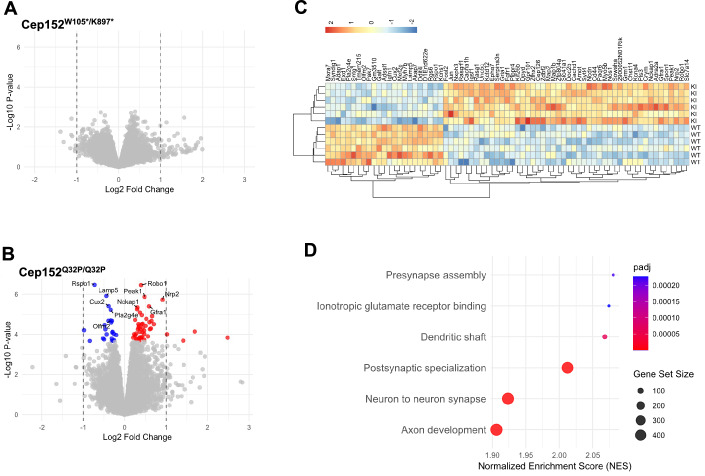
RNA-seq analysis of brain samples from Cep152^Q32P/Q32P^ and Cep152^W105*/K897*^ mice. (**A**, **B**) Volcano plots displaying the distribution of differentially expressed genes based on log2 fold change and statistical significance (−log10 *P* value). The plot is shown for (**A**) Cep152^W105*/K897*^ and (**B**) Cep152^Q32P/Q32P^ mice. Genes with FDR <0.05 are highlighted and were classified as upregulated (red) or downregulated (blue). Non-significant genes are in gray. The top five upregulated and downregulated genes are highlighted with their gene symbols. Dashed vertical lines indicate the fold-change thresholds of log2 ± 1 (fold change of 2). (**C**) Heatmap showing the expression patterns of the differentially expressed genes (FDR <0.05) between Cep152^Q32P/Q32P^ and wild-type mice. Gene expression levels are scaled for each gene to highlight relative differences. Upregulated genes are shown in red and downregulated genes are in blue. (**D**) A plot showing the results of gene ontology enrichment analysis. The plot shows the three top enriched pathways ranked by normalized enrichment score (NES) and three pathways ranked by *p* value. Pathway significance is indicated by color (adjusted *p* value, *p*_adj_), with more significant pathways in red and less significant pathways in blue. The size of each bubble represents the gene set size (number of genes in the pathway). All analyses were performed using the primary and secondary somatosensory cortices of adult mice (P60). *n* = 3 males and 3 females per genotype.

We performed differential expression analysis using RNA-seq on six RNA samples extracted from brain tissues (P60) of Cep152^W105*/K897*^ and six wild-type mice. Among the 15,449 robustly expressed genes, none were found to be significantly differentially expressed at an FDR threshold of <0.05 (Fig. 9A). In contrast, RNA-seq analysis of six RNA samples from Cep152^Q32P/Q32P^ and six wild-type mice identified 71 significantly differentially expressed genes (FDR <0.05) out of 15,423 expressed genes, comprising 23 downregulated and 48 upregulated genes (Fig. 9B,C; Dataset EV1). Gene set enrichment analysis (GSEA) of these differentially expressed genes revealed significant enrichment for multiple gene ontology (GO) terms, primarily driven by upregulated genes (Fig. 9D; Dataset EV2). Notably, the enriched GO terms were related to neuronal development processes such as axon development, dendrite development, forebrain development, and regulation of synapse structure or activity. The three GO terms with the highest normalized enrichment score (NES) were: Presynapse assembly (FDR = 2.2 × 10^−4^), Ionotropic glutamate receptor binding (FDR = 2.3 × 10^−4^), and Dendritic shaft (FDR = 6.3 × 10^−5^). Meanwhile, the three most statistically significant GO terms were: Postsynaptic specialization (FDR = 9.6 × 10^–11^), Axon development (FDR = 1.7 × 10^−9^), and neuron-to-neuron synapse (FDR = 1.7 × 10^−9^). These findings suggest that synaptic formation and function, dendritic morphology, and axon development are disrupted in Cep152^Q32P/Q32P^ mice. This aligns with the observed abnormalities in dendritic architecture, reduced spine density, and altered synaptic activity in this model.

## Discussion

CEP152 plays a critical role in centriole duplication, structural integrity, and the maintenance of cell polarity during the cell cycle (Bettencourt-Dias and Glover, 2007; Conduit et al, 2015; Takeda et al, 2020). In humans, *CEP152* variants that disrupt protein function are thought to impair cell proliferation and cause premature cell death, linking them to MCPH9 and Seckel syndrome (Kalay et al, 2011; Hussain et al, 2012; Zhang et al, 2023). In this study, we examined the pathophysiological significance of novel compound heterozygous variants, c.314 G > A,p.(W105*) and c.2689 A > T,p.(K897*), identified in a Japanese patient, along with a founder homozygous variant, c.95 A > C,p.(Q32P), found in two Saudi Arabian families. While the Japanese patient (#1) exhibited a typical MCPH9 phenotype characterized by proportionately small brain size with simplified gyri, the Saudi Arabian patients (#2 and #3) presented with severe microcephaly with marked gyral simplification, as well as ventricular enlargement, hypogenesis (#2) or hypoplasia (#3) of the corpus callosum, and cyst formation. Notably, Patient #3 exhibited polymicrogyria. These abnormalities are atypical and have not been previously reported in either MCPH9 or Seckel syndrome. A re-analysis of the whole-exome sequencing data of Patient #3 did not identify pathogenic or likely pathogenic variants in known autosomal recessive polymicrogyria-associated genes. Furthermore, no rare deleterious variants with predicted functional impact were detected. These findings therefore indicate that the cortical malformation is most likely attributable to the c.95 A > C, p.(Q32P) variant, although the contribution of unidentified modifying factors cannot be entirely excluded.

Previous studies on abnormalities in centriole-related genes, including *CEP152*, have primarily focused on their effects on cell division and survival in vitro (Hatch et al, 2010; Kalay et al, 2011), suggesting that the neuronal symptoms of MCPH arise from a reduction in neuronal numbers due to impaired neurogenesis and increased apoptosis. However, a smaller brain size does not necessarily correlate with ID. Since synaptic dysfunction is often linked to ID, it is plausible that MCPH involves impairments in synaptic morphology and function. In this context, a recent study reported that CEP152 localizes at excitatory synapses in differentiated primary cultured hippocampal neurons, and is biochemically fractionated in the postsynaptic density (PSD) (Hamada et al, 2022). These findings suggest that CEP152 dysfunction may impact not only neuronal numbers but also synaptic function, particularly in excitatory neurons. We propose that defective synaptic morphology or signaling may contribute to the neuropathophysiology of MCPH by disrupting neuronal connectivity and network function.

To model the pathogenic conditions observed in the patients we identified, we generated Cep152^W105*/K897*^ and Cep152^Q32P/Q32P^ mice. Using these mouse models, we confirmed that the p.K897* and p.Q32P variants differentially impair centriole structure and centrosome-related processes and also induce apoptotic cell death, which likely underlie the brain abnormalities seen in the patients. Importantly, our study further revealed distinct and previously unrecognized effects of these variants on neuronal development. Notably, Cep152^Q32P/Q32P^ mice exhibited more severe cortical and cerebellar abnormalities than Cep152^W105*/K897*^ mice, closely mirroring the patients’ phenotypic severity. The thickness of the deep and superficial cortical layers is reduced in the mutant cortices compared to controls. In the cerebellum, the transient increase in Purkinje cell number observed at P6 in Cep152^Q32P/Q32P^ mice may reflect premature differentiation, followed by excessive apoptosis at later stages, ultimately resulting in their reduced number at P60. Notably, the persistent mislocalization of Purkinje cells in the IGL suggests that this phenotype indicates a migration disorder during cerebellar development. Golgi staining further demonstrated that Cep152^Q32P/Q32P^ mice showed disrupted dendritic development in both cortical pyramidal neurons and Purkinje cells, with decreased spine density in cortical neurons. In contrast, Cep152^W105*/K897*^ mice exhibited normal dendritic and spine morphology in cortical neurons, as well as normal morphology of Purkinje cells. Collectively, these findings indicate that the p.Q32P variant impairs early neuronal development, whereas the compound heterozygous p.W105*/p.K897* variants primarily impair synaptic function, affecting later-stage synaptic integrity.

Electrophysiological analyses revealed that the p.W105*/p.K897* variants enhance excitatory synaptic transmission, as evidenced by increased mEPSC amplitude in layer II/III pyramidal neurons, whereas inhibitory synaptic transmission remains unaffected. Given that mEPSC amplitude reflects the unitary strength of synaptic AMPA receptors (AMPARs), these findings suggest that AMPAR trafficking and/or its function is altered in this mouse model. In this context, glycogen synthase kinase-3, a binding partner of CEP152, has been reported to regulate AMPAR trafficking and its function in cortical neurons (Wei et al, 2010; Liu et al, 2018). These results raise the possibility that the p.K897* variant impairs synaptic cycling and/or the function of AMPARs, leading to enhanced excitatory synaptic transmission and a shift in the E/I balance toward excitation. Increased excitatory drive, in combination with potential dysfunction of voltage-gated sodium and/or potassium channels, might underlie the elevated action potential firing observed in Cep152^W105*/K897*^ mice. These alterations in neuronal excitability are consistent with the epilepsy observed in Patient #1. While inhibitory neurons play essential roles in maintaining E/I balance, their pathophysiological involvement in Cep152^W105*/K897*^ mice has not yet been explored and remains to be elucidated. In contrast, the p.Q32P variant reduces both excitatory and inhibitory synaptic transmission to a similar extent, likely due to impaired network formation resulting from defects in dendritic and synaptic development. The preserved number of action potentials in Cep152^Q32P/Q32P^ mice indicates that the intrinsic function of voltage-gated sodium and potassium channels appears unaffected. These findings suggest that the p.Q32P variant primarily disrupts synaptic function, whereas the p.W105/p.K897 variants predominantly cause functional alterations in synaptic transmission and neuronal excitability. Taken together, this study highlights variant-specific contributions of *CEP152* to synaptic function and cytoskeletal organization, providing a mechanistic framework for understanding the diverse clinical phenotypes associated with *CEP152* variants.

Interestingly, mice with Hertwig’s anemia, caused by an inversion of exon 4 in *Cdk5rap2*, an MCPH3-causative gene encoding a CEP152-interacting partner, exhibit poorly branched dendritic arbors with immature spines and glutamatergic synapses in layer II/III pyramidal neurons (Zaqout et al, 2019). Phenotypic differences among MCPH-causative genes and variant types suggest that *CEP152*, *CDK5RAP2*, and other causal genes contribute to MCPH heterogeneity via distinct molecular mechanisms, with gene- and variant-specific roles in dendritogenesis, synaptogenesis, and synaptic function. The disruption of these roles underpins the variable clinical presentations in microcephaly, ranging from deficits in synaptic function to severe brain structural abnormalities.

RNA-seq analysis was performed at P60, a developmental stage during which neuronal circuit maturation, synaptic remodeling, and plasticity-related transcription are still ongoing, thereby allowing the identification of gene expression changes associated with these processes. This analysis identified differentially expressed genes exclusively in Cep152^Q32P/Q32P^ mice. Despite the severe brain phenotypes observed in Cep152^Q32P/Q32P^ mice, the most significantly upregulated pathways were related to dendritic shaft structure, postsynaptic specialization, and glutamate receptor activity—key regulators of neuronal connectivity and function. This unexpected result suggests that, while *CEP152* variants can disrupt cortical architecture, they may also trigger compensatory mechanisms at the cellular level to support synaptic integrity. These findings provide novel insights into how MCPH-related genes influence neurodevelopment, not only by shaping brain structure but also by modulating neuronal function.

Primary microcephaly is traditionally attributed to aberrant neurogenesis, with neuronal migration and the six-layered cortical architecture presumed unaffected. However, recent reports of MCPH cases suggest that neuronal migration defects may also contribute to the disorder in some instances (Murdock et al, 2011; Bilgüvar et al, 2010). Notably, we identified the *CEP152* c.95 A > C,p.(Q32P) variant in a patient with polymicrogyria and callosal hypogenesis (Shaheen et al, 2019). This variant resides in a critical region of CEP152 that interacts with PLK4, underscoring the importance of the CEP152–PLK4 interaction in neuronal migration and axon elongation during corticogenesis. Furthermore, CEP152 depletion has been shown to impair centriole amplification by PLK4, highlighting the functional significance of this interaction (Dzhindzhev et al, 2010). Recently, patients with *PLK4* variants have been reported (Dinçer et al, 2017) [PMID: 25320347], many of whom exhibit ventricular dilatation, partial agenesis of the corpus callosum, hypoplasia of cerebellum and pons, and arachnoid cysts—symptoms strikingly similar to those associated with the p.Q32P variant. Intriguingly, retinal involvement has also been reported in these patients, which further adds to the clinical overlap between the two syndromes. These findings suggest that the abnormal phenotypes in such cases may stem from impaired PLK4 function due to disrupted PLK4-CEP152 interactions.

*CEP152* variants reported thus far retain the ability to bind PLK4 and show no significant brain dysplasia beyond the common features of microcephaly, such as simplified cerebral gyri resulting from a reduced number of neurons. Since the N-terminal PLK4-binding region remains intact in CEP152-K897*, the truncated protein is likely to form a complex with PLK4 in vivo. This suggests that CEP152-K897* retains partial functionality in supporting centriole duplication and centrosome activity through PLK4-dependent pathways, albeit insufficient for proper subcellular localization of PLK4 (Fig. 6C). In contrast, the lack of the C-terminal region in CEP152-K897* abolishes its interactions with CPAP and CEP192, which are essential for full centrosome integrity. Nevertheless, retention of PLK4 binding may mitigate the severity of clinical features compared to those seen with homozygous p.Q32P or *PLK4* variants. In addition, the residual centrosomal activity may explain the survival of Cep152^W105*/K897*^ mice, whereas complete loss of CEP152 function is embryonically lethal (Bazzi & Anderson, 2014; Hamada et al, unpublished observation).

In conclusion, we expand the mutational spectrum of *CEP152*-related microcephaly by identifying novel compound heterozygous *CEP152* variants, c.314 G > A,p.(W105*) and c.2689 A > T,p.(K897*), in a patient with primary microcephaly, and revisiting the previously reported p.Q32P by identifying a new family with the same variant to confirm its founder nature. Using two mouse models, we established a clear link between these *CEP152* variants and both primary microcephaly and structural defects, highlighting variant-specific differences. This study demonstrates that the pathogenic mechanisms associated with CEP152 vary significantly depending on the type and location of the variant, providing valuable insights into the diverse effects of *CEP152* variants. These findings not only enhance our understanding of CEP152-related neurodevelopmental disorders but also lay the groundwork for the development of targeted therapeutic strategies.

## Methods

**Table:** Reagents and tools table

Reagent/resource	Reference or Source	Identifier or Catalog Number
**Experimental models**		
Mouse: ICR	SLC Japan	N/A
**Recombinant DNA**		
pCAG-EGFP		N/A
pCAG-Myc-hCEP152	This study	N/A
pCAG-Myc-hCEP152-K897*	This study	N/A
pCAG-Myc-hCEP152-W105*	This study	N/A
pCAG-Myc-hCEP152-Q32P	This study	N/A
pCAG-Myc-mPLK4	This study	N/A
pCAG-PACKmKO1	gift by Dr. F. Matsuzaki at RIKEN, Kobe, Japan	N/A
**Antibodies**		
Rabbit anti-CEP152-N	GeneTex	Cat# GTX128027
Rabbit anti-CEP152-C	Homemade	
Rabbit anti-Calbindin-D	Homemade	
Rabbit anti-Myc	Homemade	
Rabbit anti-Parvalbumin	Homemade	
Rabbit anti-Tbr2	Abcam	Cat# ab23345
Rabbit anti-activated	Cell Signaling	Cat# 9664
Caspase3		
Rabbit anti-Cux1	Gene Tex	Cat# GTX56275
Rabbit anti-GFP	Medical & Biological Laboratories	Cat# 598
Rabbit anti-phospho-Histone H3	Cell Signaling	Cat# 9701
Rabbit anti-Olig2	Proteintech	Cat#13999-1-AP
Rabbit anti-Pax6	COVANCE	Cat# PRB-278P
Mouse anti-Myc	Cell Signaling	Cat# 2276
Mouse anti-γ-tubulin	Sigma-Aldrich	Cat# T6557
Mouse anti-Arl13b	Neuromab	Cat# 75-287
Mouse anti-α-Tubulin	Sigma-Aldrich	Cat# T6199
Mouse anti-Nestin	R&D SYSTEMS	Cat# MAB2736
Mouse anti-β-Catenin	BD Biosciences	Cat# 610153
Mouse anti-α-actin	Cell Signaling	Cat# 3700
Rat anti-Ctip2	Abcam	Cat# ab18465
Rat anti-Centrin2	BioLegend	Cat# 698601
**Oligonucleotides and other sequence-based reagents**		
ssODN for Q32P-KI (CCGTGTGACACTGATGTGACTGCCCTTACTCAGTTGCAGCAGCTACTCACAGACCTCCCTCACGACATGCTGGACGATGA)	Integrated DNA Technologies	
**Chemicals, enzymes and other reagents**		
4′,6-Diamidino-2-phenylindole dihydrochloride	Sigma-Aldrich	Cat# D9542
Ethynyl-2’-deoxyuridine	FUJIFILM Wako	Cat# 050-08844
Click-iT TM Plus EdU Alexa Fluor TM 555 imaging kit	Invitrogen	Cat# C10638
Paraformaldehyde	Nacalai Tesque	Cat# 26126-25
PERMAFLUOR: anti-fading mounting medium	Thermo Scientific	Cat# TA-030-FM
FD Rapid GolgiStain kit	FD NeuroTechnologies	Cat# PK401A
Permount: mounting medium	Fisher Scientific	Cat# SP15-100
Sepasol®-RNA I Super	Nacalai Tesque	Cat# G09379-97
MGIEasy RNA Directional Library Prep Set kit	MGI	Cat# 1000006385
DNBSEQ-T7RS High-throughput Sequencing Kit	MGI	Cat# 940-000266-00
Cas9	Integrated DNA Technologies	cat# 1081060
**Software**		
Prism (version 10.1.0)	GraphPad Software	https://www.graphpad.com/features
Fiji/ImageJ	Fiji/ImageJ	https://imagej.net/software/fiji/
pCLAMP8 software	Molecular Devices	https://support.moleculardevices.com/s/article/Axon-pCLAMP-8-Electrophysiology-Data-Acquisition-Analysis-Software-Download-Page
**Other**		
Confocal laser microscope Zeiss LSM880	Zeiss	LSM880
Fluorescence microscope	Keyence	BZ-9000
Electroporator	NEPA Gene	NEPA21
Electrodes	NEPA Gene	CUY650P5
Electron microscopy	Hitachi High-Tech	HT7700
patch-clamp amplifier	Molecular Devices	Axopatch 200B

### Ethics statement

The study involving human participants was conducted in accordance with the Declaration of Helsinki and conformed to the ethical principles outlined in the Belmont Report (US Department of Health and Human Services). Ethical approval was obtained from the local review board, and informed consent was obtained from the parents or legal guardians of all enrolled participants. In addition, written informed consent for publication of patient photographs was obtained from the parent of Patient #1. The human clinical and genomic datasets are available from the corresponding author upon reasonable request. For animal experiments, we followed the guidelines for proper conduct of research and related activities in academic institutions under the jurisdiction of the Japanese Ministry of Education, Culture, Sports, Science, and Technology. All animal handling and treatment protocols were reviewed and approved by the Animal Care and Use Committee of the Institute for Developmental Research, Aichi Developmental Disability Center (approval number: 2019-013).

### Genetic analysis

Human genomic DNAs were isolated from peripheral blood and subjected to exome sequencing using a SureSelect XT Human All Exon V6 Panel (Agilent Technology) on a HiSeq platform (Illumina). The results were confirmed by Sanger sequencing.

### Animals

Mice were housed (one animal in each cage) with a 12-to-12 h-light–dark cycle, humidity (60 ± 5%), with access to food and water ad libitum in individually ventilated cages. A total of 236 animals were used in this study. In vivo analyses were carried out (*n* ≥ 3 animals/group) based on a previous study (Insolera et al, 2014). No samples or animals were excluded from the analysis. Animals were allocated to groups to balance sex and litter, minimizing subjective bias. This study was conducted and reported in accordance with the ARRIVE guidelines.

### Plasmid construction

The human *CEP152* (KIAA0912) and mouse *Plk4* cDNAs were purchased from Kazusa Genome Technologies Inc. and Applied Biological Materials (Cat# 370200140000), respectively. The cDNAs were subsequently inserted into pCAG-Myc and pCAG-GFP vectors (Addgene Inc.). For the visualization of the centrosome, we utilized pCAG-PACKmKO1 (a generous gift by Dr. F. Matsuzaki at RIKEN, Kobe, Japan), which contains a centrosome targeting signal derived from the scaffold protein AKAP450. To generate the three CEP152 variant proteins, CEP152-W105* [c.314 G > A,p.(W105*)], CEP152-K897* [c.2689 A > T,p.(K897*)], and CEP152-Q32P [c.95 A > C,p.(Q32P)], site-directed mutagenesis was performed using pCAG-Myc-CEP152 as a template and a KOD-Plus Mutagenesis kit (Toyobo, Cat# SMK-101). DNA sequencing was employed to confirm the accuracy of all constructed plasmids.

### Antibodies

Anti-CEP152 antibodies targeting the N-terminal region (anti-CEP152-N; GeneTex, Cat# GTX128027, 1:2000) and the C-terminal region (anti-CEP152-C, 1:10,000 [IHC], 1:1000 [Western blotting]) (Hamada et al, 2022) were used. Polyclonal rabbit anti-Calbindin-D, anti-Myc, and anti-Parvalbumin antibodies were produced as described previously (Inaguma et al, 1991; Kurobe et al, 1992; Mizutani et al, 2013). The following rabbit polyclonal antibodies were used: anti-Tbr2 (Abcam, Cat# ab23345, 1:300), anti-activated Caspase3 (aCasp3; Cell Signaling, Cat# 9664, 1:400), anti-Cux1 (Gene Tex, Cat# GTX56275, 1:300), anti-GFP (Medical & Biological Laboratories, Cat# 598, 1:1000), anti-phospho-Histone H3(Ser10) (PH3) (Cell Signaling, Cat# 9701, 1:400), anti-Olig2 (Proteintech, Cat#13999-1-AP, 1:500), and anti-Pax6 (COVANCE, Cat# PRB-278P, 1:500). Mouse monoclonal anti-Myc (Cell Signaling, Cat# 2276, 1:1000), anti-γ-tubulin (Sigma-Aldrich, Cat# T6557, 1:1000), anti-Αrl13b (Neuromab, Cat# 75-287, 1:1000), anti-α-Tubulin (Sigma, Cat# T6199, 1:200), anti-Nestin (R&D SYSTEMS, Cat# MAB2736, 1:100), anti-β-Catenin (BD Biosciences, Cat# 610153, 1:1000), and anti-β-actin (Cell Signaling, Cat# 3700, 1:5000). Rat monoclonal anti-Ctip2 (Abcam, Cat# ab18465, 1:500) and anti-Centrin2 (BioLegend, Cat# 698601, 1:200) were also used. Alexa Fluor 488-, 568-, and 647-labeled IgG (Abcam, Cat# ab150077, ab175471, and ab150075, respectively) were used as secondary antibodies at a 1:1000 dilution. 4’, 6-diamidino-2-phenylindole (DAPI) (Sigma-Aldrich, Cat# D9542, 0.2 μg/ml) was used to stain DNA.

### Cell culture, transfection, immunoprecipitation and Western blotting

Cercopithecus aethiops kidney cell line COS7, (CRL-1651, ATCC; RRID: CVCL_0224) and mouse neuroblastoma cell line N2a (CCL-131, ATCC; RRID: CVCL_0470), which are not listed as commonly misidentified cell lines by the International Cell Line Authentication Committee, were maintained in Dulbecco’s modified Eagle’s medium supplemented with 10% fetal bovine serum (FBS) (Hamada et al, 2023). Cells were used for experiments within 10-15 passages. Primary MEFs were isolated from E14.5 embryos and dissociated by trypsinization. Cells were maintained in high-glucose DMEM (HyClone) supplemented with 10% FBS (Gibco) and 1 mM penicillin, streptomycin, and L-glutamine. Transient transfections were performed using PEImax reagent (Polysciences, Cat# 24765), following the manufacturer’s instructions. Protein concentrations were determined using a BCA protein assay kit (Pierce, Cat# 23227), with bovine serum albumin (BSA) as the standard. SDS-PAGE and Western blotting were performed as described previously (Hamada et al, 2021). For immunoprecipitation analyses (Hamada et al, 2022), COS7 cells expressing Myc-PLK4 and GFP-CEP152 were lysed in RIPA buffer containing 50 mM Tris-HCl (pH 8.0), 150 mM NaCl, 1% NP-40, 0.5% deoxycholate, 0.1% SDS, 1 mM Na3VO4, and a protease inhibitor cocktail (Nacalai Tesque, Cat# 25955-11). After centrifugation at 21,500 × *g* for 10 min, the supernatants were incubated with anti-Myc antibody for 120 min at 4 °C, followed by incubation with Protein A-Sepharose beads (Nacalai Tesque, Cat# 29435-56) for 60 min at 4 °C. After three washes with RIPA buffer, the precipitates were subjected to Western blotting using anti-Myc and anti-GFP antibodies (Mizutani et al, 2013).

### Immunohistochemistry

Mice were deeply anesthetized using a combination of medetomidine (0.75 mg/kg), butorphanol (5 mg/kg), and midazolam (4 mg/kg) (Kawai et al, 2011), followed by perfusion with 4% paraformaldehyde (PFA). Brain tissues were fixed in 4% PFA, processed, and either stored as frozen samples in cryoprotectant medium or embedded in agar at room temperature (RT). Sections (12-μm thick) were cut using a freezing microtome (CM1900, Leica Microsystems) and mounted on glass slides. Alternatively, tissues were sectioned with a vibrating microtome (VT1000, Leica Microsystems) to obtain 100-μm-thick sections. Sections were blocked for 1 h in phosphate-buffered saline (PBS) containing 0.5% Triton X-100 and 0.1% BSA, then incubated overnight at 4 °C with primary antibodies diluted in PBST (PBS containing 0.05% Triton X-100). The following day, sections were incubated with secondary antibodies in PBST for 1 h, followed by DAPI staining for nuclear visualization. After three washes with PBST, the stained sections were mounted using an anti-fading mounting medium (PERMAFLUOR; Thermo Scientific, Cat# TA-030-FM). Fluorescence imaging was performed using a confocal laser microscope (LSM880, Carl Zeiss).

### In utero electroporation

*In utero* electroporation was carried out using pregnant Slc:ICR mice (Japan SLC Inc.) as previously described (Hamada et al, 2023), with minor modifications. Briefly, following anesthesia, the indicated plasmids were co-injected with pCAG-GFP into the lateral ventricles of the embryonic dorsal forebrain. Electroporation was performed using a NEPA21 electroporator (NEPA Gene) with five pulses of 35 V (50 ms per pulse, 450 ms intervals). Plasmids were electroporated into cells of the somatosensory cortex within the parietal lobe. Brains were collected at the indicated embryonic or postnatal timepoints following electroporation and processed for analysis. All experiments were conducted during the daytime. No electroporated animals were excluded from the study or died during the experimental procedures.

### Generation of the CEP152 mutant mouse models, Cep152^W105*/K897*^ and Cep152^Q32P/Q32P^

The Cep152^W105*/K897*^ and Cep152^Q32P/Q32P^ mouse strains were generated using the improved-genome editing via the Oviductal Nucleic Acids Delivery (i-GONAD) technique (Gurumurthy et al, 2019). For Cep152^W105*/K897*^, the crRNAs 5′-AGACTTTGAAAGGGAAAAAG-3′ and 5′-TTTAAACAGTAGAGGAACTG-3′ were selected as a pair targeting exon 2, while 5′-CAGAGCCCAGCAGCAGGAGT-3′ and 5′-GAGGATGGTGAGGATGGTGA-3′ were selected as a pair targeting exon 20 (Table EV2). For Cep152^Q32P/Q32P^, the crRNA 5′- TAGCTGCTGCAACTGAGTAA-3′ was chosen to target exon 3 (Table EV2). ssODN (5′- CCGTGTGACACTGATGTGACTGCCCTTACTCAGTTGCAGCCGCTACTCACAGACCTCCCTCACGACATGCTGGACGATGA-3′), Cas9 (cat# 1081060), and tracrRNA (cat# 1072534) were obtained from Integrated DNA Technologies Inc. For the i-GONAD procedure, Cas9, crRNAs, and tracrRNA were dissolved in Opti-MEM and injected into the oviducts of Slc:ICR pregnant mice 0.5 day post conception. The electroporation parameters were as follows: poring pulse (voltage, 50 V; pulse length, 5 ms; pulse interval, 50 ms; number of pulses, 3; decay rate, 10%; polarity switch, +), and transfer pulse (voltage, 10 V; pulse length, 50 ms; pulse interval, 10 ms; number of pulses, 3; decay rate, 40%; polarity switch, +/−). The genotype of Cep152^W105*/K897*^ was determined by PCR using genomic DNA derived from the mice with the exon 2 mutation (forward primer, 5′-GCCATGAATTGCTGTCATATGGCTGC-3′; reverse primer, 5′-ACCATAAATCACGGTACTCGTCC-3′) and the exon 20 mutation (forward primer, 5′-GTAGGTGGAAACAGCTGTGC-3′; reverse primer, 5′-GCTGGTGCACGTCTCAGAGC-3′). Genotyping of Cep152^Q32P/Q32P^ mice was performed by digesting PCR products with AluI, using genomic DNA extracted from the mice (forward primer, 5′-GGCTTCAGGGCAGCTTGGGC-3′; reverse primer, 5′-CGACATGCTGGACGATGAGC-3′).

### Golgi–Cox staining and spine analysis

Golgi–Cox staining was performed using the FD Rapid GolgiStain kit (FD NeuroTechnologies, Cat# PK401A) according to the manufacturer’s instructions, with minor modifications. One-month-old male mice were deeply anesthetized with isoflurane, decapitated, and their brains were rapidly removed and immersed in FD Solution AB (A = 1:1) for 2 weeks at RT in the dark. The brains were then transferred to FD Solution C or a tissue-protectant solution (20% sucrose and 15% glycerol in water) and stored at 4 °C in the dark for 72 h. Subsequently, 100-µm-thick coronal sections were prepared in the tissue-protectant solution. The sections were mounted and stained according to the kit’s instructions. After dehydration, the slides were mounted with Permount (Fisher Scientific, Cat# SP15-100). Z-stack images (20–30) with 1 µm intervals for Golgi-stained dendrites and 0.1 µm intervals for spines were acquired using 20× and 100× lenses, respectively, on a BZ-9000 microscope. The number and length of basal dendrite branches were analyzed using NeuronJ, a plugin for Fiji. The number of spines on each apical dendrite within 50–100 µm from the cell soma was counted.

### Electron microscopy

The embryonic tissue from three groups, including wild-type, Cep152^W105*/K897*^ and Cep152^Q32P/Q32P^ were fixed by 4% PFA and 2.5% glutaraldehyde in 0.1 M PBS (pH 7.4) and postfixed in the same fixative over a few nights. For TEM, sample preparation and observation were performed as described previously with minor modifications (Otani et al, 2020). Briefly, samples were washed with PBS, postfixed in 2% osmium tetroxide (OsO₄, Nisshin EM Co., Tokyo, Japan) in PBS, dehydrated in a graded ethanol series, treated with dehydrated acetone, and embedded in Quetol 812 epoxy resin (Nisshin EM Co.). Ultrathin sections were cut with a diamond knife, stained with uranyl acetate and lead citrate, and observed with HT7700 (Hitachi High-Tech). For observation by serial block-face scanning electron microscopy, sample preparation, observation, and analyses of acquired data were performed as described previously with minor modifications (Morizawa et al, 2022). Briefly, tissues were washed in PBS, and then sequentially treated with 2% OsO₄ in 1.5% potassium ferrocyanide, 1% filtered thiocarbohydrazide, 2% OsO₄, 2% uranyl acetate, and finally 0.66% lead aspartate. Milli-Q water washes were performed after every incubation step. The samples were then dehydrated through a graded ethanol series, infiltrated with molecular sieve–dried acetone, and embedded in Durcupan resin (Sigma) containing 5% carbon-resin. After polymerization, blocks were mounted on rivets, trimmed, coated with gold to improve conductivity, and imaged in a scanning electron microscope (Merlin, Carl Zeiss) equipped with a 3View in-chamber ultramicrotome system and OnPoint detector (Gatan, Inc.). Images were collected at 1.3-1.4 kV, a resolution of 5 nm per pixel with a dwell time of 1.0-µs per pixel, and sections were cut at thicknesses 40 nm. Images were acquired to cover dividing cells at the ventricular surface, and 19, 71, and 27 cells were observed in two WT, three Cep152^W105*/K897*^, and 2 Cep152^Q32P/Q32P^ mice, respectively. The images were handled with FIJI (https://fiji.sc/) using the TrakEM2 plugin for alignment (Cardona et al, 2012).

### Electrophysiological analyses

The analyses were conducted as previously described (Nishijo and Momiyama, 2016). Briefly, 300-μm-thick coronal cortical sections were prepared from P7 to 10 mice using a micro slicer (PRO7； Dosaka) in ice-cold cutting Krebs solution. The slices were then transferred to a holding chamber containing standard Krebs solution and incubated at RT. During recordings, each slice was superfused with standard Krebs solution at a rate of 3–4 ml/min. To record synaptic currents, patch pipettes made from borosilicate glass capillaries were filled with CsCl-based internal solution. For current-clamp recordings, patch pipettes were filled with K-gluconate-based internal solution. Whole-cell recordings were conducted for pyramidal neurons within cortical layers Ⅱ/Ⅲ using a patch-clamp amplifier (Axopatch 200B, Molecular Devices) and pCLAMP8 software (Molecular Devices).

### Gene expression analysis

Primary and secondary somatosensory areas from adult mice (P60) were dissected, and RNA was extracted using Sepasol®-RNA I Super (Nacalai Tesque Inc., Cat# G09379-97). For Cep152^Q32P/Q32P^, the sample size was five males and one female per genotype and for Cep152^W105*/K897*^ it was three males and three females per genotype. mRNA libraries for sequencing were prepared using the MGIEasy RNA Directional Library Prep Set kit (MGI, Cat# 1000006385), and sequencing was performed with the DNBSEQ-T7RS High-throughput Sequencing Kit (FCL PE150) V3.0 (MGI, Cat# 940-000266-00). RNA-seq reads were aligned to the mouse genome using the STAR aligner (v2.7.11), and gene-level read counts were obtained. Differential expression analysis was conducted using edgeR with raw count data. Genes with low counts were filtered using the filterByExpr function. Differential expression analysis was carried out with the EdgeR Package using a quasi-likelihood negative binomial generalized log-linear model (EdgeR function glmQLFit), with statistical significance set at FDR <0.05. Gene ontology enrichment analysis was performed using the R package fgsea.

### Statistical analyses

Sample sizes for experiments typical of those reported in the field without the use of statistical methods were used in this study, and no randomization was used to collect the data. For all cell imaging experiments, cell counting and trace analysis were performed by a staff member blinded to the experimental conditions. Statistical analyses were conducted using GraphPad Prism 10 (GraphPad Software Inc.) or the R statistical package (version 4.5.1). Results are presented as mean ± SD. For comparisons between two groups, a Welch’s *t*-test was used. For comparisons involving more than three groups, a one-way analysis of variance (ANOVA) was performed, followed by a Tukey–Kramer least significant difference (LSD) test for multiple comparisons. Statistical significance was defined as *p* < 0.05. Data normality was not assessed in this study. Box and whisker plots represent the median (horizontal bars), the 25th to 75th percentiles (box edges), and the whiskers extend to the largest and smallest observed values that are not outliers. The cross inside the boxes indicates the mean.

## Supplementary information

Table EV1

Table EV2

Dataset EV1

Dataset EV2

Peer Review File

Source data Fig. 2

Source data Fig. 3

Source data Fig. 4

Source data Fig. 5

Source data Fig. 6

Source data Fig. 7

Source data Fig. 8

Expanded View Figures

## Data Availability

The datasets produced in this study are available in the following databases: RNA-seq: NCBI Gene Expression Omnibus (GEO) GSE325064 (https://www.ncbi.nlm.nih.gov/geo/query/acc.cgi?acc=GSE325064). The source data of this paper are collected in the following database record: biostudies:S-SCDT-10_1038-S44321-026-00427-3.
