# Supplementary material for: Distinct pathophysiological mechanisms of CEP152 variants in microcephaly and brain abnormalities
Source: EMBO Mol Med. 2026 May 5;18(6):2180–212. doi: 10.1038/s44321-026-00427-3 (PMC13270125; doi:10.1038/s44321-026-00427-3)
Supplement: Supplementary file 1 — Table EV1 [file 44321_2026_427_MOESM1_ESM.pdf]

Table EV1. Clinical findings of patients (n.a.= not applicable)

	Patient 1	Patient 2 24DG0698	Patient 3 (published in PMID: 30214071) 17DG0680	AJHG 87: 40-51, 2010	AJHG 87: 40-51, 2010	AJHG 87: 40-51, 2010	Frontiers in Genetics 13:1052915, 2023	Clin Genet 2013: 83: 446-451	Nat. genet 2010	Nat. genet 43: 23-26, 2010	Nat. genet 43: 23-26, 2010	Nat. genet 43: 23-26, 2010	Nat. genet 43: 23-26, 2010	Nat. genet 43: 23-26, 2010	Nat. genet 43: 23-26, 2010	Nat. genet 43: 23-26, 2010	Apem 28: 225-230, 2023	Apem 28: 225-230, 2023	Glob Med Gene 11: 20-24, 2024
**Sex**	M	F	M	F	M	F	M	M	M	F	M	F	M	F	M	M	F	F	M
**Mutation1**	p.Trp105*	p.Gln32Pro	p.Gln32Pro	p.Gln265Pro	p.Gln265Pro	p.Gln265Pro	p.Arg354*	p.Asn1226del	c.261+1G>C	c.261+1G>C	c.261+1G>C	c.261+1G>C	c.261+1G>C	p.Tyr678*	c.261+1G>C	p.Lys667Arg	p.Trp105Ter	p.Trp105Ter	p.Tyr678Ter
**Mutation2**	p.Lys897*	p.Gln32Pro	p.Gln32Pro	p.Gln265Pro	p.Gln265Pro	p.Arg987Ter	c.1414-14A>G	p.Leu1050Pro	c.261+1G>C	c.261+1G>C	c.261+1G>C	c.261+1G>C	c.261+1G>C	c.2694+1G>T	c.261+1G>C	p.Arg1404Ass*14	p.Tyr678Ter	p.Tyr678Ter	p.Ser1323Ter
**Height (cm)**	74.5 cm at 1.75-year-old	40 cm at birth	N.A	N.A	N.A	N.A	93 cm at 7-year-old	N.A	N.A	N.A	N.A	N.A	N.A	N.A	N.A	N.A	130.6 cm at 11-year-old	108.7 cm at 6-year-old	112.0 cm at 5-year-old
**Short Stature (<-2SD)**	-3.0 SD at 1.75-year-old	-5.0 SD at birth	(+)	(-)	(-)	(-)	<-3 SD	N.A	-8/-9 SD at 10-year-old	-4/-5 SD at 8-year-old	-6/-7 SD at 20-year-old	-7/-8 SD at 5.5-year-old	-5/-6 SD at 5-year-old	-2.3 SD at birth	-5/-6 SD at 7-year-old	-3/-4 SD at 13.5-year-old	-2.28 SD	-1.66 SD	+1.0 SD
**Head Circumference (cm)**	37.0 cm at 1.75-year-old	23.5cm at birth	36cm at 7-year-old	45-46 cm at 10.5-year-old	45-46 cm at 7.5-year-old	44-45 cm at 18-year-old	31 cm at 7-year-old	N.A	N.A	N.A	N.A	N.A	N.A	N.A	N.A	N.A	47 cm at 11-year-old	43 cm at 6-year-old	49.6cm at 5-year-old
**Microcephaly (<-2SD)**	-8.1 SD	-7.7 SD	-12 SD	<-2 SD	<-2 SD	<-2 SD	<-3 SD	-13 SD at 24-year-old	-7/-8 SD at 10-year-old	-7/-8 SD at 8-year-old	-10/-11 SD at 20-year-old	-5/-6 SD at birth	-8 SD at 5-year-old	-3.6 SD at birth	-11 SD at 38-year-old	-6/-7 SD at 13.5-year-old	<-3 SD	<-3 SD	-1.0 SD
**Developmental Delay**	(+)	(+)	(+)	(-)	(-)	(-)	N.A	(-)	(+)	(+)	(+)	(+)	(+)	(+)	(+)	(+)	(-)	(-)	N.A.
**Intellectual Disability**	(+)	N.A	(+)	(-)	(-)	(-)	(+)	(-), speak complete sentences	N.A	N.A	N.A	N.A	N.A	N.A	N.A	N.A	Borderline	N.A.	N.A.
**Developmental Disorders**	(+)	Dear	N.A	(-)	(-)	(+), Moderate cognitive movement	N.A	Self injuries	N.A	N.A	N.A	N.A	N.A	N.A	N.A	N.A	(+), Cognitive disorder, Behavioral disorder, Attention disorder	(+), Cognitive disorder, Behavioral disorder	N.A.
**Brain MRI**	simplified gyri	simplified gyri. Delayed cerebral sulcation for gestational age. The gyral pattern is simplified with anterior to posterior gradient. There is a large left para midline CSF filled interhemispheric cyst noted in the posterior aspect of the flax cerebri displacing the adjacent occipital lobe. There is underdeveloped Sylvian opercultisation. There is lack of development of the frontal horn. Severe hypogenesis of the corpus callosum.	MRI performed at 7 days of age: simplified gyri, polymicrogyria, severe callosal hypogenesis, lateral ventricle dilatation, small right cerebellar hemisphere and inferior vermis, hypoplastic pons MRI performed at 28 months old: large medial and dorsal cyst communicating with the third and left lateral ventricles and causing enlargement of the left cerebellar hemicranium with also right lateral ventricle dilatation. Significant interval increase of the cyst size and ventricular dilatation is noted. The cortex is slightly thick in both frontotemporal regions mainly left with few small gyri and likely polymicrogyria on the left medial side. Small posterior fossa is noted with mainly small right cerebellar hemisphere and inferior vermis as well as hypoplastic pons.	N.A	N.A	No major abnormalities	No abnormalities	N.A	N.A	N.A	N.A	N.A	N.A	N.A	N.A	N.A	No abnormalities	(+), Hypothalamic hamartoma	(+), High signal in the white matter area of the frontal–parietal lobe, Periventricular white matter area of the bilateral parietal lobe, Loss of the right anterior cerebral artery on magnetic resonance angiography
**Epilepsy**	(+)	(-)	(-)	(-)	(-)	(-)	(-)	N.A	N.A	N.A	N.A	N.A	N.A	N.A	N.A	N.A	N.A	N.A.	(+)
**Facial Dysmorphism**	(+)	(-)	(+)	(+)	(+)	(+)	(+)	(-)	(+)	(+)	(+)	(+)	(+)	(+)	(+)	(+)	(+)	(+)	N.A.
**Visual Impairment**	(-)	(+)	(+)	(-)	(-)	(-)	(-)	N.A	N.A	N.A	N.A	N.A	N.A	N.A	N.A	N.A	N.A	N.A	N.A.
**Hearing Impairment**	(+)	(-)	(-)	(-)	(-)	(-)	(-)	N.A	N.A	N.A	N.A	N.A	N.A	N.A	N.A	N.A	N.A	N.A	N.A.
**Cleft Palate**	(+)	(-)	(-)	N.A	N.A	N.A	N.A	N.A	(-)	(-)	(-)	(+)	(-)	(-)	(-)	(-)	N.A	N.A	N.A.
**Tooth Abnormalities**	(+)	N.A	(-)	N.A.	N.A.	N.A.	(+), Selective tooth agenesis	N.A	(+), Selective tooth agenesis, Enamel	(+), Selective tooth agenesis,	(+), Selective tooth agenesis,	(+), Selective tooth agenesis,	(+), Selective tooth agenesis	N.A	N.A	(-)	N.A	N.A	N.A.
**Limb Abnormalities**	(+), fifth finger clinodactyly	N.A	(-)	N.A.	N.A.	N.A.	(+), Fifth finger clinodactyly, Pes planus	N.A	(+), Fifth finger clinodactyly, Pes planus	(+),Hypoplasia of the middle phalanx of the fifth digit	(+), fifth finger clinodactyly, hypoplasia of the middle phalanx of the fifth	(+), Fifth finger clinodactyly	(+), Fifth finger clinodactyly	(+), Fifth finger clinodactyly	N.A	(+), Fifth finger clinodactyly	N.A	N.A	N.A.
**Skeletal Abnormalities**	(+), scoliosis	N.A	(+), scoliosis	N.A	N.A	N.A	N.A	N.A	(+), Eleven ribs, Delayed bone age, Pes planus	(+), Eleven ribs, Pes planus	(+), Eleven ribs, Pes planus	(+), Eleven ribs, Delayed bone age	(+), Eleven ribs, Delayed bone age	(+), Delayed bone age	N.A	(+), Pes planus, Scoliosis	(+), Scolosis	(+), Right-slipped capital femoral epiphysis, Accelerated bone age	N.A.

References

Guernsey, D. L., Jiang, H., Hussin, J., Arnold, M., Bouyakdan, K., Perry, S., ⋯ Samuels, M. E. (2010). Mutations in centrosomal protein CEP152 in primary microcephaly families linked to MCPH4. American journal of human genetics, 87(1), 40–51.

Kalay, E., Yigit, G., Aslan, Y., Brown, K. E., Pohl, E., Bicknell, L. S., ⋯ Wollnik, B. (2011). CEP152 is a genome maintenance protein disrupted in Seckel syndrome. Nature genetics, 43(1), 23–26

Sajid Hussain, M., Marriam Bakhtiar, S., Farooq, M., Anjum, I., Janzen, E., Reza Toliat, M., Eiberg, H., Kjaer, K. W., Tommerup, N., Noegel, A. A., Nürnberg, P., Baig, S. M., & Hansen, L. (2013). Genetic heterogeneity in Pakistani microcephaly families. Clinical genetics, 83(5), 446–451.

Zhang, L., Teng, Y., Hu, H., Zhu, H., Wen, J., Liang, D., Li, Z., & Wu, L. (2023). Two novel variants in CEP152 caused Seckel syndrome 5 in a Chinese family. Frontiers in genetics, 13, 1052915.

Li, W., Lu, X., Shu, J., Cai, Y., Li, D., & Cai, C. (2024). Novel Variants of CEP152 in a Case of Compound-Heterozygous Inheritance of Epilepsy. Global medical genetics, 11(1), 20–24.

Park, J., Jeon, M., Maeng, S., Kwon, D. K., Kim, S., & Lee, J. E. (2023). Central precocious puberty with hypothalamic hamartoma: the first case reports of 2 siblings with different phenotypes of Seckel syndrome 5. Annals of pediatric endocrinology & metabolism, 28(3), 225–230.
