# Supplementary material for: Distinct pathophysiological mechanisms of CEP152 variants in microcephaly and brain abnormalities
Source: EMBO Mol Med. 2026 May 5;18(6):2180–212. doi: 10.1038/s44321-026-00427-3 (PMC13270125; doi:10.1038/s44321-026-00427-3)
Supplement: Supplementary file 13 — Expanded View Figures [file 44321_2026_427_MOESM13_ESM.pdf]

Expanded View Figures

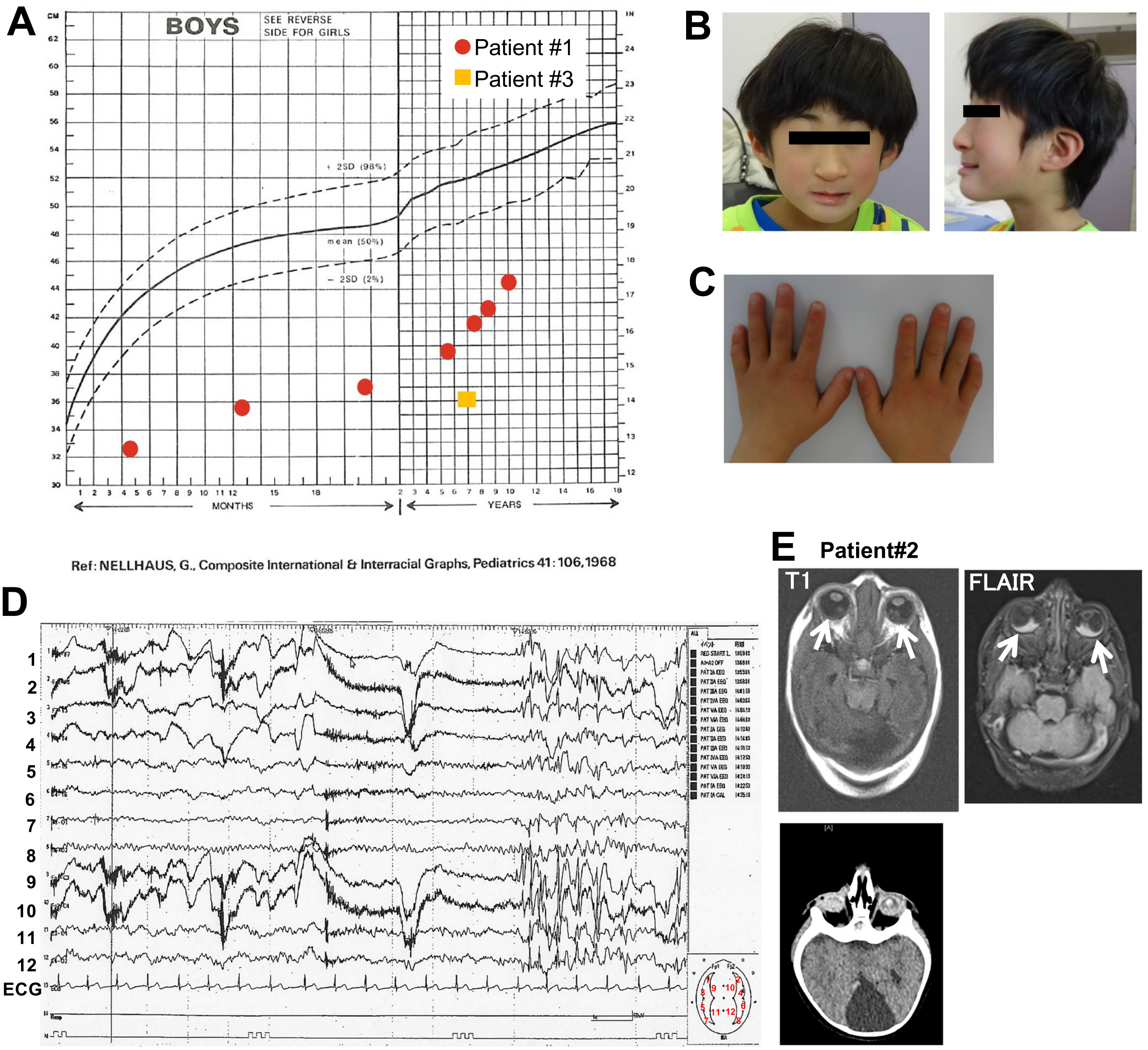

Figure EV1. Clinical characteristics of the patients.

(**A**) OFC measurements of Patients #1 and #3 plotted against population mean values. (**B**, **C**) Facial features (**B**) and clinodactyly of the fifth finger (**C**) of Patient #1 at 6 years of age. Hypotelorism, short palpebral fissures, micrognathia, a broad nasal bridge, and low-set ears were observed. Informed consent for publication of these photographs was obtained from the patient's parents. (**D**) Electroencephalogram (EEG) of Patient #1 showing irregular spike-and-wave complexes localized to the frontal and temporal regions. (**E**) MRI (upper) and CT (bottom) images of Patient #2 demonstrating bilateral retinal detachment due to persistent hyperplastic primary vitreous.

© The Author(s)

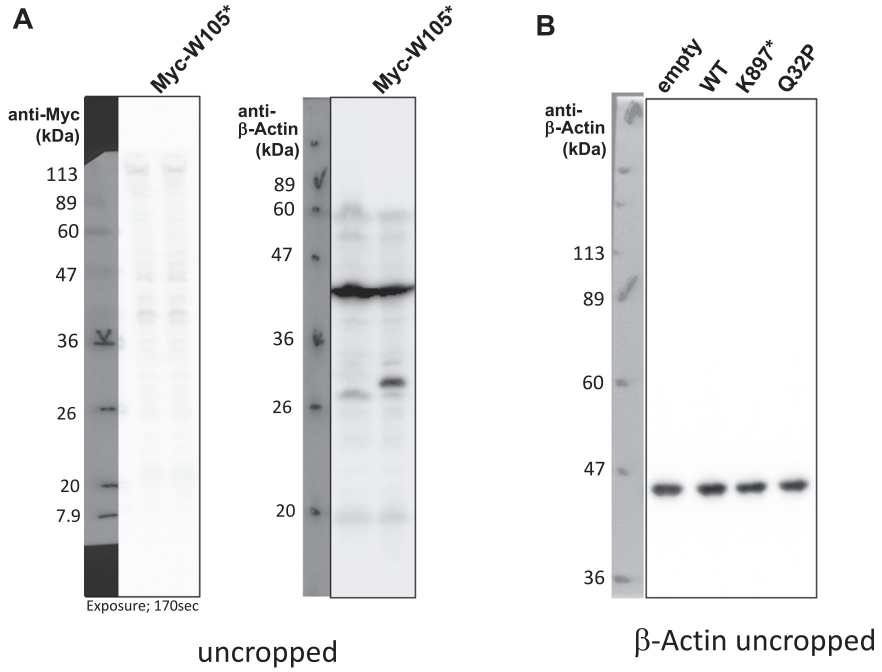

Figure EV2. Full, uncropped Western blot images corresponding to Fig. 2A.

(**A**) Full, uncropped Western blot images corresponding to Fig. 2A,b. (**B**) Full, uncropped Western blot images with anti-β-actin corresponding to Fig. 2A,a.

© The Author(s)

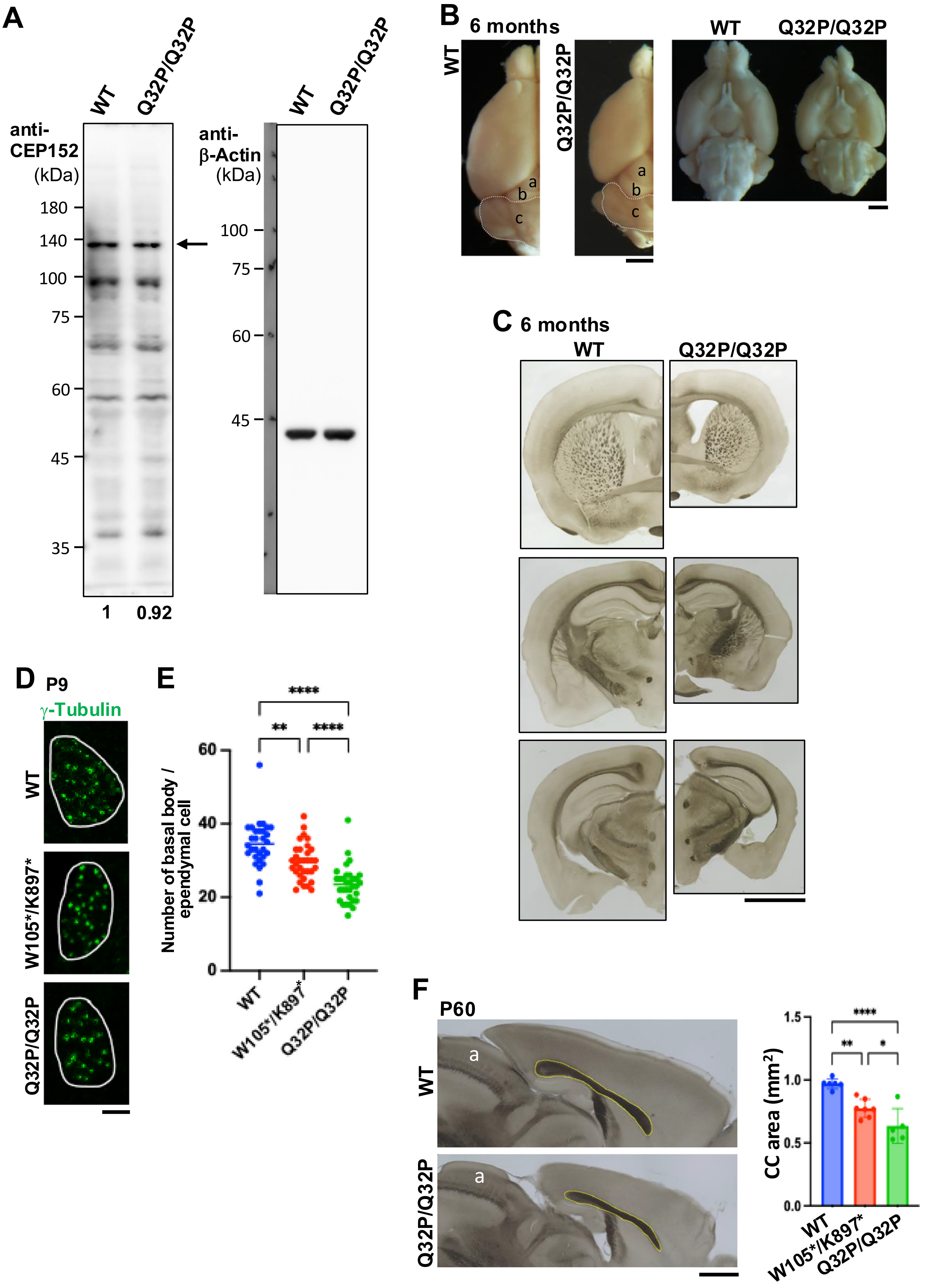
A
WT
Q32P/Q32P
anti-CEP152
(kDa)
180
140
100
75
60
45
35
1
0.92
anti-β-Actin
(kDa)
100
75
60
45
B
6 months
WT
Q32P/Q32P
a
b
c
C
6 months
WT
Q32P/Q32P
D
P9
γ-Tubulin
WT
W105*/K897*
Q32P/Q32P
E
Number of basal body / ependymal cell
60
40
20
0
****
**
****
WT
W105*/K897*
Q32P/Q32P
F
P60
WT
Q32P/Q32P
a
CC area (mm²)
1.5
1.0
0.5
0.0
****
**
*
WT
W105*/K897*
Q32P/Q32P

Figure EV3. Expression of variant proteins in Cep152$^{W105*/K897*}$ and Cep152$^{Q32P/Q32P}$ mice.

(**A**) Expression of endogenous CEP152 and CEP152-Q32P in the brains of WT and Cep152$^{Q32P/Q32P}$ mice. Whole-tissue extracts were subjected to SDS-PAGE (7.5% gel), followed by western blotting with an anti-CEP152-C. β-actin was used as a loading control and visualized on a 10% gel. Band intensities of CEP152 proteins were quantified and normalized to β-actin levels. Full uncropped blots are shown. (**B**) Dorsal and ventral views of whole brains from WT and Cep152$^{Q32P/Q32P}$ mice at 6 months of age. Regions used for macroscopic comparison are indicated (a superior colliculus; b inferior colliculus; c cerebellum outlined by a dotted line). Note that both the cerebral cortex and cerebellum appear markedly reduced in size compared with the midbrain structures, resulting in unusually prominent exposure of the superior and inferior colliculi. This gross phenotype is consistent with that observed in Fig. 3C. (**C**) Vibratome coronal sections from WT and Cep152$^{Q32P/Q32P}$ mice at 6 months, showing global brain morphology. (**D**) Immunostaining of basal bodies using anti-γ-tubulin at the base of motile cilia in ependymal cells lining the ventricular surface in WT, Cep152$^{W105*/K897*}$, and Cep152$^{Q32P/Q32P}$ mice at P9. Basal bodies were visualized as markers of individual motile cilia. Individual cells are indicated by white outlines. (**E**) Quantification of (**D**). Basal bodies were used to quantify motile cilia number. Data were presented as mean ± SD. $n = 4$ animals per genotype. Cell counts: WT, 30; Cep152$^{W105*/K897*}$, 33; Cep152$^{Q32P/Q32P}$, 32. Tukey–Kramer LSD, $^{**}p = 0.0011$, $^{****}p < 0.0001$. (**F**) Corpus callosum morphology from vibratome sagittal sections at P60. The outlined region (yellow) indicates the area used for quantification. The bar graph shows the callosal area in WT and Cep152$^{Q32P/Q32P}$ mice. Data were presented as mean ± SD. $n = 6, 7, 6$, for WT, Cep152$^{W105*/K897*}$, and Cep152$^{W105*/K897*}$, respectively. Tukey–Kramer LSD, $^{*}p = 0.0398$, $^{**}p = 0.0031$, $^{****}p < 0.0001$. Scale bars: 5 μm (**D**), 500 μm (**F**), 2 mm (**B**, **C**).

© The Author(s)

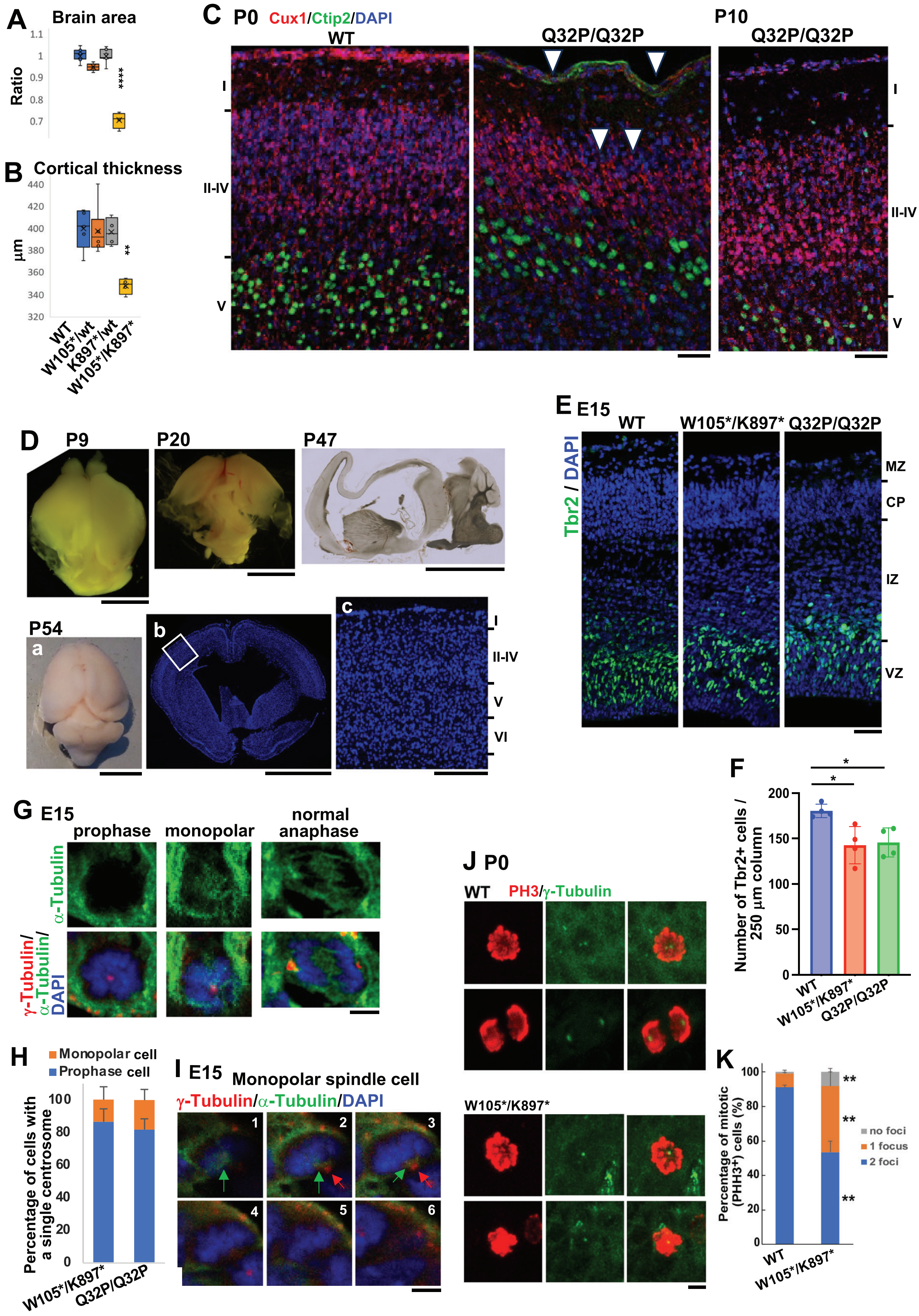

A Brain area
Ratio
B Cortical thickness
µm
WT
W105*/wt
K897*/wt
W105*/K897*
C P0 Cux1/Ctip2/DAPI
WT
Q32P/Q32P
P10 Q32P/Q32P
I
II-IV
V
D P9 P20 P47
P54
E E15
WT
W105*/K897*
Q32P/Q32P
Tbr2 / DAPI
MZ
CP
IZ
VZ
F
Number of Tbr2+ cells / 250 µm column
G E15
prophase
monopolar
normal anaphase
α-Tubulin
γ-Tubulin/α-Tubulin/DAPI
H
Monopolar cell
Prophase cell
Percentage of cells with a single centrosome
I E15 Monopolar spindle cell
γ-Tubulin/α-Tubulin/DAPI
J P0
WT PH3/γ-Tubulin
W105*/K897*
K
Percentage of mitotic (PH3+) cells (%)
no foci
1 focus
2 foci

Figure EV4. Gross and histological analyses of brain morphology in Cep152$^{W105*/K897*}$ and Cep152$^{Q32P/Q32P}$ mice.

(**A**) Quantification of brain area in WT, Cep152$^{W105*/wt}$, Cep152$^{K897*/wt}$, and Cep152$^{W105*/K897*}$ mice at P0. Values are shown as ratios relative to WT. Tukey–Kramer LSD, ****$p < 0.0001$. (**B**) Cortical thickness measured in the same cohort at P0. Sample sizes: (**A**) $n = 12$, 5, 7, 4; (**B**) $n = 6$, 4, 6, 4 for WT, Cep152$^{W105*/wt}$, Cep152$^{K897*/wt}$, and Cep152$^{W105*/K897*}$, respectively. Tukey–Kramer LSD, **$p = 0.0011$. Box and whisker plots show the median (horizontal line), the 25th and 75th percentiles (box boundaries), and the whiskers extend to the largest and smallest values that are not outliers. A cross inside the boxes indicates the mean. (**C**) Representative images of cortical layers in WT (P0) and Cep152$^{Q32P/Q32P}$ (P0 and P10), immunostained for Cux1 (upper-layer neurons), Ctip2 (deep-layer neurons), and nuclei (DAPI). Defects observed in the Cep152$^{Q32P/Q32P}$ section were indicated by arrowheads. Note that the anti-Cux1 antibody also detects a Golgi-associated Cux1 isoform that is predominantly present during early postnatal stages. (**D**) Gross appearance of brains from Cep152$^{Q32P/Q32P}$ mice at P9, P20, and P54, imaged using a fluorescent stereomicroscope, and a vibratome sagittal section at P47. A coronal section of the P54 brain (a) was stained with DAPI (b), and the boxed area in (b) is magnified to illustrate cortical layer organization (c). Note that the yellow coloration observed in the P9 and P20 images results from the characteristics of the fluorescent stereomicroscope filter and does not represent intrinsic tissue color. (**E**) Representative images of cortical sections from WT, Cep152$^{W105*/K897*}$, and Cep152$^{Q32P/Q32P}$ mice at E15.5 stained with anti-Tbr2 (a basal progenitor marker) and DAPI. (**F**) Quantification of (**E**). The number of Tbr2-positive cells per section was counted. $n = 4$ animals per genotype. Tukey–Kramer LSD, *$p = 0.0188$ (WT vs Cep152$^{W105*/K897*}$), *$p = 0.0286$ (WT vs Cep152$^{Q32P/Q32P}$). (**G**) Immunostaining of cerebral cortices from Cep152$^{Q32P/Q32P}$ mice at E15.5 using anti-α-tubulin (spindle; green) and anti-γ-tubulin (centrosome; red). (**H**) Quantification of (**G**). Percentages of mitotic cells with a single centrosome in prophase or meta/anaphase were quantified. $n = 5$ and 4 for Cep152$^{W105*/K897*}$ and Cep152$^{Q32P/Q32P}$, respectively. Cell counts: Cep152$^{W105*/K897*}$, 35; Cep152$^{Q32P/Q32P}$, 35. (**I**) Sequential images of a monopolar mitotic cell in the VZ of a Cep152$^{Q32P/Q32P}$ mouse at E15. Green and red arrows indicate spindle fibers and the centrosome, respectively. (**J**) Representative images of mitotic glial cells in the cortical plate at P0. Slices from WT and Cep152$^{W105*/K897*}$ mice were stained for PH3 (red) and γ-tubulin (green) as shown in Fig. 4A. (**K**) Quantification of γ-tubulin foci in mitotic cells shown in (**J**). The foci were scored in mitotic glial cells. $n = 4$ animals per genotype. Cell counts: WT, 151; Cep152$^{W105*/K897*}$, 200. Data were presented as mean ± SD for each animal. Scale bars: 5 mm (**D**; P9–P47 and P54, panels a and b), 500 µm (**D**; P54, panel c), 50 µm (**C**, **E**), 5 µm (**G**, **I**, **J**). Welch's *t*-test, 2 foci (**$p = 0.0013$), 1 focus (**$p = 0.0064$), no foci (**$p = 0.0058$).

 © The Author(s)

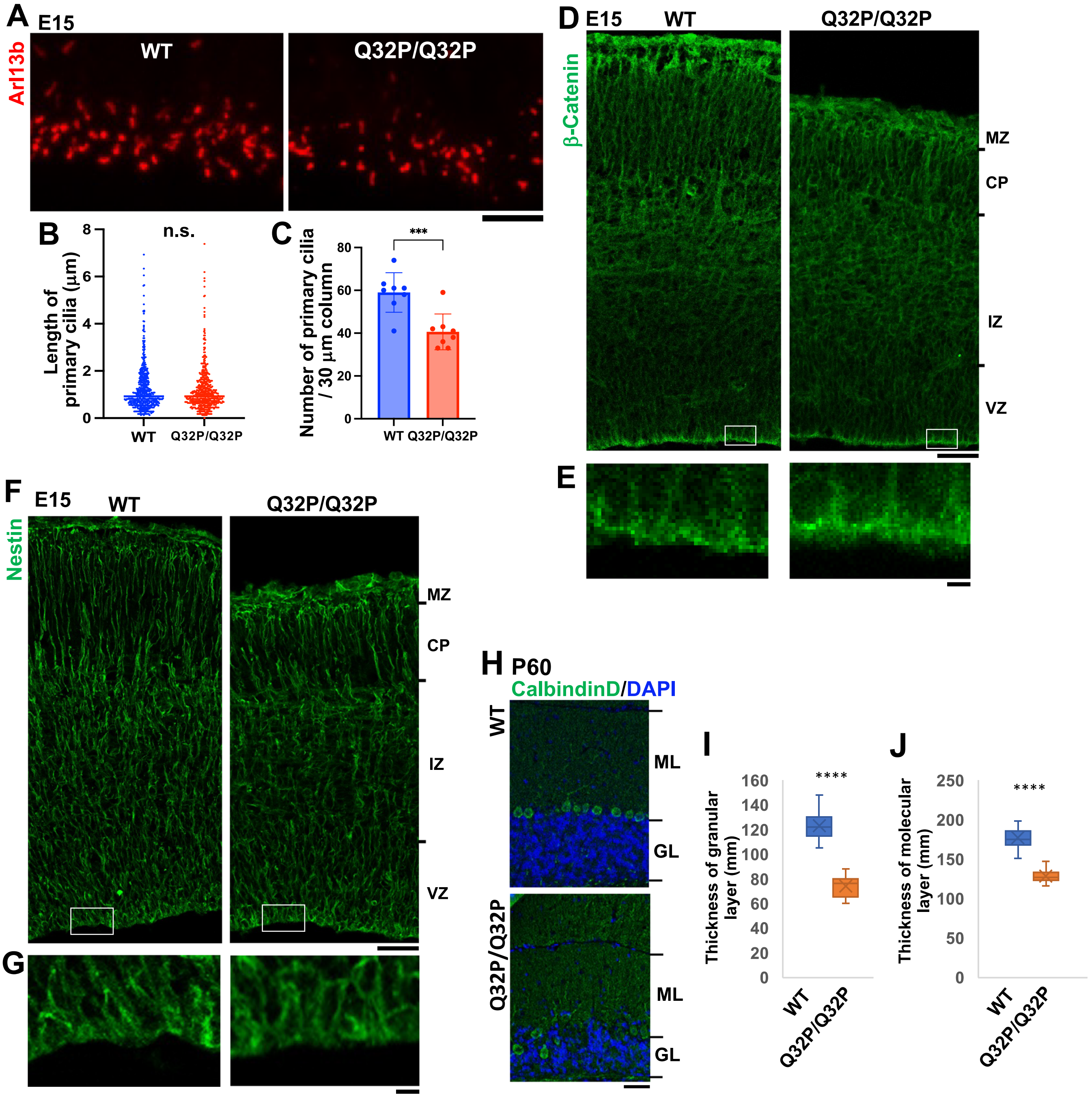

Figure EV5. Additional histological analyses of brain structure in Cep152$^{Q32P/Q32P}$ mice.

(A) Representative images of primary cilia in VZ progenitor cells at E15.5. Cortical slices were stained with anti-Arl13b (red). (B, C) Quantification of (A). (B) Length of primary cilia were measured. $n = 8$ and 6 for WT and Cep152$^{Q32P/Q32P}$, respectively. Cell counts: WT, 598; Cep152$^{Q32P/Q32P}$, 556. (C) The number of primary cilia per 30 μm column was counted. $n = 4$ animals per genotype; 8 fields of view for WT and Cep152$^{Q32P/Q32P}$, respectively. Welch's *t*-test, ***$p = 0.0010$. (D) Representative images of cortical slices (E15.5) stained with anti-β-catenin (an adherens junction marker). (E) Magnified images from squares in (D). (F) Representative images of cortical slices (E15.5) stained with anti-nestin (a radial glia marker). (G) Magnified images from squares in (F). (H) Representative images of cerebellar cortex sections in the lobule 4/5 from WT and Cep152$^{Q32P/Q32P}$ mice at P60 stained with anti-CalbindinD (green) and DAPI (blue). ML molecular layer, GL granular layer. (I, J) Quantification of (H): thickness of the granular layer (I) and molecular layer (J). Box and whisker plots show the median (horizontal line), the 25th and 75th percentiles (box boundaries), and the whiskers extend to the largest and smallest values that are not outliers. A cross inside the boxes indicates the mean. Data were presented as mean ± SD. $n = 10$ and 8 for WT and Cep152$^{Q32P/Q32P}$. Welch's *t*-test, ****$p < 0.0001$. Scale bars: 10 μm (A), 50 μm (D, F, J), 5 μm (E, G).

© The Author(s)
