# Supplementary material for: Distinct pathophysiological mechanisms of CEP152 variants in microcephaly and brain abnormalities
Source: EMBO Mol Med. 2026 May 5;18(6):2180–212. doi: 10.1038/s44321-026-00427-3 (PMC13270125; doi:10.1038/s44321-026-00427-3)
Supplement: Supplementary file 5 — Peer Review File [file 44321_2026_427_MOESM5_ESM.pdf]

EMBO Molecular Medicine

Distinct pathophysiological mechanisms of CEP152 variants in microcephaly and brain abnormalities

Nanako Hamada, Lama AlAbdi, Tomoko Uehara, Looprasertkul Sasikarn, Takuma Nishijo, Reut Suliman-Lavie, Mais Hashem, Majid Alfadhel, Shatha Alhefdhi, Brahim Tabarki, Malak Ali Alghamdi, Ikuko Iwamoto, Toshiki Takenouchi, Kenjiro Kosaki, Sagiv Shifman, Seiji Mizuno, Nobuhiko Ohno, Fowzan Alkuraya, and Koh-ichi Nagata

Corresponding author: Koh-ichi Nagata (knagata@inst-hsc.jp)

Review Timeline:

Submission Date:	11th May 25
Editorial Decision:	17th Jun 25
Revision Received:	27th Jan 26
Editorial Decision:	25th Feb 26
Revision Received:	25th Mar 26
Accepted:	30th Mar 26

Editor: Zeljko Durdevic

Transaction Report:

(Note: With the exception of the correction of typographical or spelling errors that could be a source of ambiguity, letters and reports are not edited. Depending on transfer agreements, referee reports obtained elsewhere may or may not be included in this compilation. Referee reports are anonymous unless the Referee chooses to sign their reports.)

17th Jun 2025

Dear Dr. Nagata,

Thank you for the submission of your manuscript to EMBO Molecular Medicine. We have now received feedback from the three reviewers who agreed to evaluate your manuscript. As you will see from the reports, all three referees recognize potential interest of the study, but they also raise serious and partially overlapping concerns that should be addressed in a major revision. If you would like to discuss further the points raised by the referees, I am available to do so via email or video. Let me know if you are interested in this option.

Further consideration of a revision that addresses reviewers' concerns in full will entail a second round of review. EMBO Molecular Medicine encourages a single round of revision only and therefore, acceptance or rejection of the manuscript will depend on the completeness of your responses included in the next, final version of the manuscript. For this reason, and to save you from any frustrations in the end, I would strongly advise against returning an incomplete revision.

We would welcome the submission of a revised version within three months for further consideration. Please let us know if you require longer to complete the revision.

Please use this link to login to the manuscript system and submit your revision: https://embomolmed.msubmit.net/cgi-bin/main.plex

I look forward to receiving your revised manuscript.

Yours sincerely,

Zeljko Durdevic

Zeljko Durdevic
Senior Editor
EMBO Molecular Medicine

*****

When submitting your revised manuscript, please carefully review the instructions that follow below. We perform an initial quality control of all revised manuscripts before re-review; failure to include requested items will delay the evaluation of your revision.

We require:

1) A .docx formatted version of the manuscript text (including legends for main figures, EV figures and tables). Please make sure that the changes are highlighted to be clearly visible.

2) Individual production quality figure files as .eps, .tif, .jpg (one file per figure). For guidance, download the 'Figure Guide PDF': (https://www.embopress.org/page/journal/17574684/authorguide#figureformat).

3) A .docx formatted letter INCLUDING the reviewers' reports and your detailed point-by-point responses to their comments. As part of the EMBO Press transparent editorial process, the point-by-point response is part of the Review Process File (RPF), which will be published alongside your paper.

4) A complete author checklist, which you can download from our author guidelines (https://www.embopress.org/page/journal/17574684/authorguide#submissionofrevisions). Please insert information in the checklist that is also reflected in the manuscript. The completed author checklist will also be part of the RPF.

5) Please note that all corresponding authors are required to supply an ORCID ID for their name upon submission of a revised manuscript.

6) It is mandatory to include a 'Data Availability' section after the Materials and Methods. Before submitting your revision, primary

datasets produced in this study need to be deposited in an appropriate public database, and the accession numbers and database listed under 'Data Availability'. Please remember to provide a reviewer password if the datasets are not yet public (see https://www.embopress.org/page/journal/17574684/authorguide#dataavailability).

In case you have no data that requires deposition in a public database, please state so in this section. Note that the Data Availability Section is restricted to new primary data that are part of this study.

7) For data quantification: please specify the name of the statistical test used to generate error bars and P values, the number (n) of independent experiments (specify technical or biological replicates) underlying each data point and the test used to calculate p-values in each figure legend. The figure legends should contain a basic description of n, P and the test applied. Graphs must include a description of the bars and the error bars (s.d., s.e.m.). See also 'Figure Legend' guidelines: https://www.embopress.org/page/journal/17574684/authorguide#figureformat

8) At EMBO Press we ask authors to provide source data for the main manuscript figures. You will receive a separate email with instructions for providing source data with your revised manuscript, including how to upload and organize the files.

9) Our journal encourages inclusion of *data citations in the reference list* to directly cite datasets that were re-used and obtained from public databases. Data citations in the article text are distinct from normal bibliographical citations and should directly link to the database records from which the data can be accessed. In the main text, data citations are formatted as follows: "Data ref: Smith et al, 2001" or "Data ref: NCBI Sequence Read Archive PRJNA342805, 2017". In the Reference list, data citations must be labeled with "[DATASET]". A data reference must provide the database name, accession number/identifiers and a resolvable link to the landing page from which the data can be accessed at the end of the reference. Further instructions are available at .

10) We replaced Supplementary Information with Expanded View (EV) Figures and Tables that are collapsible/expandable online. A maximum of 5 EV Figures can be typeset. EV Figures should be cited as 'Figure EV1, Figure EV2" etc... in the text and their respective legends should be included in the main text after the legends of regular figures.

- For the figures that you do NOT wish to display as Expanded View figures, they should be bundled together with their legends in a single PDF file called *Appendix*, which should start with a short Table of Content. Appendix figures should be referred to in the main text as: "Appendix Figure S1, Appendix Figure S2" etc.

- Additional Tables/Datasets should be labeled and referred to as Table EV1, Dataset EV1, etc. Legends have to be provided in a separate tab in case of .xls files. Alternatively, the legend can be supplied as a separate text file (README) and zipped together with the Table/Dataset file.

See detailed instructions here:

.

11) The paper explained: EMBO Molecular Medicine articles are accompanied by a summary of the articles to emphasize the major findings in the paper and their medical implications for the non-specialist reader. Please provide a draft summary of your article highlighting

- the medical issue you are addressing,

- the results obtained and

- their clinical impact.

This may be edited to ensure that readers understand the significance and context of the research. Please refer to any of our published articles for an example.

12) Author contributions: You will be asked to provide CRediT (Contributor Role Taxonomy) terms in the submission system. These replace a narrative author contribution section in the manuscript.

13) A Conflict of Interest statement should be provided in the main text.

14) Every published paper now includes a 'Synopsis' to further enhance discoverability. Synopses are displayed on the journal webpage and are freely accessible to all readers. They include a short stand first (maximum of 300 characters, including space) as well as 2-5 one-sentences bullet points that summarizes the paper. Please write the bullet points to summarize the key NEW findings. They should be designed to be complementary to the abstract - i.e. not repeat the same text. We encourage inclusion of key acronyms and quantitative information (maximum of 30 words / bullet point). Please use the passive voice. Please attach

these in a separate file or send them by email, we will incorporate them accordingly.

Please also suggest a striking image or visual abstract to illustrate your article as a PNG file 550 px wide x 300-600 px high.

15) Include a Reagents and Tools Table as part of the Methods section, which can be downloaded from our author guidelines (https://www.embopress.org/page/journal/17574684/authorguide#structuredmethods)

EMBO Molecular Medicine has a "scooping protection" policy, whereby similar findings that are published by others during review or revision are not a criterion for rejection. Should you decide to submit a revised version, I do ask that you get in touch after three months if you have not completed it, to update us on the status.

***** Reviewer's comments *****

Referee #1 (Remarks for Author):

The manuscript by Hamada et al. performs comparative analysis of the consequences on brain development of CEP152 locus mutations, newly identified in the genome of three microcephaly patients. They generate for the first time two mouse mutant lines that carry the human CEP152 pathological variants and phenocopy the human pathology. They report unexpected differences regarding the penetrance of the phenotypes, likely related to the capacity of the pathological forms of CEP152 proteins to be stabilized and localized at the centrosome. More, they show differences in the neuronal excitability comparing the two mutant brains, which has not been reported thus far after mutation of a centriolar protein. The manuscript is convincingly illustrated, written and discussed. Nonetheless, there are some experiments missing to establish a causal relationship between centrosome defects (illustrated by the defects in the number of foci in mitotic cells) and CEP152 loss-of-function, while the authors claim no defects in centrosome structure and do not explore centriole number or integrity problems. Furthermore, some tissue dysplasia reported in one of the mutants (ventricle enlargement, neuronal layer disorganization) can be attributed to cilia defects, which have not been explored in this manuscript and could be related to centriole architecture impairment again. Centrosome-related defects have to be better characterized, especially in the context of the literature.
1) Lines 3-4 page 7, the authors cannot claim that: "expression of these variants did not affect centrosome structure, as indicated by normal gamma-tubulin staining". Centrosome structure cannot be only assessed after a gamma-tubulin staining. Centrosomes are made of centrioles and peri-centriolar material (PCM) and gamma-tubulin is typically a PCM marker, with a localization that changes at different stages of the cell cycle. It has to be combined with another centriole marker. A glutamylated tubulin staining could be used for that purpose. If the best characterization of centriole architecture is by electron microscopy, super-resolution microscopy with a combination of centriole markers can also unravel defects in centriole architecture. The authors should further investigate the organization of centrioles after super-resolution microscopy.
2) Expression and stability of the pathological variants are investigated in a normal context in N2A cells, meaning in the presence of a normal version of CEP152. The patients exhibit only pathological versions of CEP152 in their genome. If the authors perform siRNA against CEP152 to remove the normal version of the protein in N2A cells and perform the same investigation regarding pathological variant localization, stability and centriole/centrosome architecture, do they obtain the same results?
3) The authors need to investigate any defects in the localization of CEP152 partner proteins in the mouse mutant brains and more importantly centrosome (centriole and PCM) and cilium architecture. Indeed, the enlargement of ventricular area observed in Figure 3A-C could be related to defects in motile cilia at the surface of ependymal cells as a consequence of impaired circulation of the cerebrospinal fluid. Such defects in cilia could be due to defects in centriole architecture in the CEP152 Q32P/Q32P mutant brain, which has not been explored. If the case, centriole defects should be different in the W105/K897 mutant brain, as no ventricular enlargement can be observed.
4) Lines 30-32, page 9, the authors propose that as cell death is most prevalent in the IZ, it primarily occurred in differentiated neurons. The position of dying cells in the neuroepithelium is not sufficient to conclude on dying cells identity as progenitor cells always detach before undergoing apoptosis as described in the literature. Therefore, if the authors want to conclude on the identity of dying cells, they should perform a double staining with the tubulin ßIII marker for example.
5) Apoptosis and premature neuronal differentiation have been shown as consequences of mitotic defects in microcephaly brain models. The authors have reported apoptosis in their CEP152 mutant brains. As they propose that differentiated neurons are the ones dying, there is a possibility that progenitors having experienced mitotic defects exit prematurely the cell cycle to undergo neuronal differentiation before dying. If the case, they might see a decrease in the TBR2-positive basal progenitor population and an increase in the tubulin ßIII-positive population. The authors need to explore this possibility.
6) Defects in the number of mitotic foci indeed reflect defects in centrosome number and are typically analysed in mitotic cells, and prometaphase especially. Nonetheless, the number of gamma-tubulin foci does not enable to investigate centrosome integrity defects, as PCM can organize in the absence of centrioles. Can the authors check the presence and number of centrioles at each pole after a centrin staining?
7) Centriole loss after CPAP removal (another centriole protein) results in primary cilium loss, radial glia cell detachment and massive heterotopia as a consequence of neuroepithelial junction disorganization. As the authors report some tissue disorganization in the cerebral cortex and cerebellum in their Q32P/Q32P homozygous mutant model, can it also result from similar (even if milder) developmental defects in the neuroepithelium?

Minor points:
1) Can the authors provide higher magnifications of centrosomes in Fig2B?
2) In figure 4, panel G reporting the % of mitotic cells should be placed in D, as it is in the middle of the % of apoptotic cell analysis.
3) In figure 4, can the authors better explain in the figure legend which region is analysed in panels E and H.
4) The authors record different defects in neuron excitability in their two CEP152 mutant brains. Can they discuss more these observations in the discussion section, do they have any explanation regarding the differences observed?

Referee #2 (Remarks for Author):

The work of Hamada and colleagues is very important because it provides a new perspective on the genetic mechanisms underlying defects in brain growth such as microcephaly, using both the phenotypic description of 3 patients and mouse mutants reproducing the human phenotype. The novelty of this study is to have shown a differential effect of 3 different CEP152 mutations on mitosis progression, the loss of interaction between the mutated CEP152 centrosomal protein and one of its partners, as well as long-term defects that these mutations cause in the development of the cortex, but also of neuronal dendrites, and cerebellum.
However, I recommend that the following points be explored in greater depth in order the results meet expectations in the field.
Major points regarding the human phenotype:
The main concern is whether or not there is severe cerebral or cortical malformations defined by tha authors as agenesis of the corpus callosum, polymicrogyria, ventricular dilatation, interhemispheric cyst, associated with microcephaly. The information provided by the authors does not allow this to be confirmed:
i) On axial images (figure 1C), the corpus callosum appears to exist anteriorly in patients 2 and 3, coronal and strict sagittal images are required.
ii) According to the results and Figure 1C, Patient 2 has no polymicrogyria, and the polymicrogyria claimed by the authors for patients 3 carrying the same variants (c.95A>C) is not obvious on the MRI images (figure 1C); an axial and coronal T1-weight sequence is essential to make a conclusion. This aspect resembles microcephaly with a very simplified gyration.
iii) Ventricular dilatation may be secondary to an obstacle (is the aqueduct of sylvius permeable?), or may reflect insufficient growth (a vacuo dilatation), but in both cases, this is not classified as a cerebral malformation by the international classifications.
iv) Interhemispheric cyst is not a cerebral malformation either, as it is extracerebral and has developed at the expense of the arachnoid.
Without these additional imaging data, it seems impossible to affirm that patients 2 and 3 have a cerebral or cortical malformations associated with their microcephaly. Microcephaly with very simplified gyration and interhemispheric cyst +/- hypoplasia of the corpus callosum would be more appropriate, and this should be modified in the title, summary, results and discussion. If the OFC of patient 2 and 3 were < -10 SD, the term micrencephaly could be used.
Major points regarding cell biology in vitro experiments or in vivo models:
Several data raise questions:
1. Do the Cep152 Q32P/Q32P mutant have a cortical and cerebellar malformations?
P8 lines 14-16: what is this "abnormal depression of the cortical surface"? This work would be greatly improved if the authors could well characterize the cortical plate of these Q32P/Q32P mutant mice by showing images of the whole brain and images of the cortical plate at objective 40 in Figure 3 or a supplementary figure for the 9 brains that have been analysed. It is important to compare brain structure of mouse and human brains mutated for CEP152 to verify the adequacy of the model.
On Figure 3 A and B, corpus callosum is present. It is important to mention this in the results and to compare it with brain MRI images of patients.
P9 lines 1-3 and Figure 3M: "Dentritic development of Purkinje cells also appeared impaired in Q32P/Q32P mice", however, dendritic cells seem to be increased. It would be important to quantify this at P6 and to and then try to interpret this result with the apoptosis at P9.
What is the structure of the centrosome after overexpression of the variants in vitro or in Ki mutant mice in vivo?
"The variants do not affect the structure of the centrosome", however the experiments shown in figure 2 and in Results (paragraph "biological and biochemical properties of the CEP152 variants ...." and γtubulin staining alone) are not sufficient to affirm that centrosome structure is not affected. Knowing the role of CEP152 in centriole biogenesis, this work would be greatly improved if the authors could provide data to support their hypothesis by investigating the centrosome using centriole staining, high magnification and high resolution microscopy.
The authors show an increase in the proportion of cells in mitosis with a single point of gamma tubulin and an increased proportion of cells in prophase/prometaphase and conclude that there is a high proportion of monopolar spindles.
i) Could the authors show these images in 3D to demonstrate whether these cells have a monopolar spindle in only one side of the metaphase plate or are almost normal prophase with a single centrosome instead of two?

ii) "p9 line 22: "These finding indicate a prolonged pro/prometaphase duration in mitotic progenitors". Unless authors film the cells using live-microscopy to measure mitosis duration, this can't be affirmed. This experiment would be important and show the outcome of these cells.
iii) P10 lines 1-3: "these results demonstrate that CEP152 is critical for maintaining centrosome integrity and mitotic fidelity". Experiments are lacking to affirm this since i) only the W105* mutant shows the absence of CEP152, ii) centrosome integrity has not been clearly demonstrated here, iii) images of monopolar spindles have not yet been shown in 3D.
Specific comments:
Introduction
P4 lines 1-7: Seckel syndrome is characterized by proportionate short stature or primordial dwarfism, associated with an extreme smallness of the head, with a peculiar aspect resembling "bird-headed", and is related to PCNT variants. The subgroup of patients with CEP152 variants do not exhibit proportionate dwarfism and microcephaly (height and OFC were not equally reduced), as expected in patients with Seckel syndrome. Prefer here "microcephalic primordial dwarfism".
P4 line 14: Please change severe or multiple brain malformations for severe microcephaly with marked gyral simplification or micrencephaly (see above)
Results
Developmental stages of mice should be indicated in the text to help the reader to understand the altered developmental processes. This must also be added in all the figures
P5 line 11: NICU: please define
P5 line 19: "bilateral intraocular detachment, consistent with retinal detachment": This could be a new phenotype related to CEP152, CT-scan n Figure 3 A and B must be shown / added in Figure 1 to affirm this sentence.
P5 line 29: patient 3 is blind. Could the authors explain why? Is there ophthalmological examination or eye imaging available?
P5 line 32-33: photographs of patient 3 must be shown in supplementary figure 2.
P6 line 3-4: ERG and VEP should be added in supplementary figure 2.
P7 line1: in Guernsey' article, it's CEP152-R987* and not -R986*, please verify.
P8 line 9: "this reduction was more pronounced in the superficial layers compared to the deep layers": what statistical test did the authors perform to compare both variables, groups and superficial vs deep layers thickness, and affirm this sentence and the following one? This is not mentioned in the methods or the legend to figure 3.
P8 line 18 and Suppl figure 5C: What is the yellow coloration? It is not explained in the results or on the figure legend. The cerebrum and cerebellum structure seem to be respected as shown in Figure 3 DFH. Could the authors explain this?
P8 -P9: see above
P9 lines 30-32: "Although cell death ... was most prevalent in the IZ, suggesting that it primarily occurred in differentiated neurons rather than in progenitors". It is important to demonstrate this hypothesis by a double immunostaining.
P10 line 19: "cerebellar hypoplasia and malformations": which malformations? There is no obvious malformation in Figure 3.
P11 lines 8-10: Could this decrease of dendritic arborisation be due to the reduction of neuronal density?
P11 lines 26-30: Do the authors suggest changes in the excitatory / inhibitory balance? Could the authors confirm this hypothesis by showing this in IF?
Discussion
P14 lines 6-8 and p15 line 28: reword this sentence, in particular multiple cerebral malformations, unless these malformations are shown more clearly on MRI in patients 2 and 3 (see above)
P14 lines 13-14: ID is present in many MOPDII patients, this is not an argument.
P14 lines 25-27: Please rephrase after taking into account new experiments aimed at better characterising the structure of the centrosome
P14 lines 32-34: Could the authors explain more the arguments in favor of this hypothesis?
P15 lines 1-8: Could the authors also discuss the possibility of an imbalance between excitatory and inhibitory neurons?
P15 lines 27-34: to comment on the results of transcriptomic data, could the authors replace at what stage the RNASeq has been performed (P60) and the developmental processes that are still ongoing or maintained at that stage?
Methods
Precise the statistical tests that were used (see above) and the imaging protocol for mitosis experiments
Figures and Tables
Figures 2-8: developmental stages of mice should be indicated in each figure to help the reader to understand the altered developmental processes. This should also be added in the results
Figure 2:
Show the whole blot for Myc-CEP152-W105*
Please enlarge Fig 2C
Figure 3E: Precise what statistical tests were used
Figure 4A: add a 3D image of monopolar spindles
I did not find Table 1. Is it Supplementary Table 1?
Supplementary Figure 2:
- There is too many OFC values for patient 1 between age 6 and 10y that makes the OFC curve confusing. This should be clarified.
- Add OFC values of patients 2 and 3 to the OFC curve of patient 1
- Add photographs and ERG/VEP of patient 3
Supplementary Figure 6A: I would point out to the authors that the selected image shows a lagging chromosome in the WT
Supplementary Table 1:

Supplementary Table 1 is incomplete. At least 3 previously reported are lacking. This should be updated. Values of OFC and height of patients (at least in SD) should be added to define the severity of microcephaly, characterize short stature as primordial dwarfism or not and to state that one patient is more severe than another.
The references should be written in the title of the columns and cited below the table as bibliographic references
Please correct the spelling errors
References
P3 line 11: the first 3 references do not concern NDDs

Referee #3 (Comments on Novelty/Model System for Author):

In this study, Nagata et al. report three CEP152 variants identified in microcephaly patients and characterize their distinct subcellular localization patterns. By generating two knock-in mouse models, the authors further investigate the phenotypic consequences in cortical and cerebellar size, as well as electrophysiological and gene expression profiles. While the in vivo findings provide valuable insights, the lack of mechanistic studies in a cultured cell model significantly weakens the novelty and depth of the reported CEP152 variants.

Referee #3 (Remarks for Author):

Centrosomal protein CEP152 is implicated in autosomal recessive primary microcephaly, yet the detailed mechanistic link between CEP152 mutations and microcephaly remains unclear. In this study, Nagata et al. report three CEP152 variants identified in microcephaly patients and characterize their distinct subcellular localization patterns. By generating two knock-in mouse models, the authors further investigate the phenotypic consequences in cortical and cerebellar size, as well as electrophysiological and gene expression profiles. While the in vivo experiments are well-designed and the results appear convincing, the mechanistic insights into how the two CEP152 variants affect centrosome function, particularly centrosome duplication, require further strengthening.

Major points:

1. In Fig. 2A, the authors overexpress CEP152-WT and its variants in N2A cells, concluding that the expression levels of the mutants are comparable to CEP152-WT. However, since expression levels can vary between vectors, it is essential to examine and quantify endogenous CEP152 levels in the brains of the two knock-in mouse models using immunoblotting. Additionally, the altered localization of CEP152 variants observed in Fig. 2B should be validated using mouse embryonic fibroblast (MEF) cells derived from the knock-in models to confirm the findings in a physiologically relevant context.

2. In Fig. 4, the authors propose that cell death primarily occurs in differentiated neurons rather than progenitor cells. To strengthen this conclusion, co-staining of Caspase-3 with specific neuronal markers and quantification of neuron numbers across different cortical layers (as shown in Fig. 3D and F) are necessary. This would provide more robust evidence supporting the observed cell death phenotype.

3. Representative images are required to accompany the quantification data presented in Fig. 4G and H. Furthermore, the number of Purkinje cells should be quantified from the images in Fig. 3K-N to provide a more comprehensive analysis of cerebellar abnormalities.

4. Several centrosomal dysfunctions, such as defects in centrosome duplication or disengagement, could underlie the mitotic phenotypes observed in Fig. 4A-C. To further elucidate the impact of the two CEP152 variants on centrosome function, the following experiments using MEF cells from the knock-in models are recommended: 1) quantify the centriole number; 2) examine and quantify the centrosomal localization of PLK4 and other duplication factors (such as CEP192); 3) quantify the percentage of abnormal mitotic cells.

Minor points:

1. The evolutionary conservation of the mutation sites between humans and mice should be presented to highlight their functional significance.

2. The age of the mice used in each experiment should be clearly labeled in Fig. 3 and 4 to ensure reproducibility and contextual clarity.

3. The ventricular size shown in Fig. 3A should be quantified to provide a more objective assessment of the observed phenotype.

Reviewer's comments

Referee #1 (Remarks for Author):

1) Lines 3-4 page 7, the authors cannot claim that: "expression of these variants did not affect centrosome structure, as indicated by normal gamma-tubulin staining". Centrosome structure cannot be only assessed after a gamma-tubulin staining. Centrosomes are made of centrioles and peri-centriolar material (PCM) and gamma-tubulin is typically a PCM marker, with a localization that changes at different stages of the cell cycle. It has to be combined with another centriole marker. A glutamylated tubulin staining could be used for that purpose. If the best characterization of centriole architecture is by electron microscopy, super-resolution microscopy with a combination of centriole markers can also unravel defects in centriole architecture. The authors should further investigate the organization of centrioles after super-resolution microscopy.

Response: We agree with the reviewer's concern that centrosome structure cannot be accurately assessed based solely on γ-tubulin staining. As our original intent in Fig. 2B was to illustrate the subcellular distribution of CEP152-K897*, CEP152-W105*, and CEP152-Q32P rather than to evaluate their effects on centrosome architecture, we have deleted the statement "Notably, the expression of these variants did not affect centrosome structure, as indicated by … (Fig. 2B)" (p. 7, lines 3 -4 in the original manuscript) from the revised manuscript. Instead, we have made a description without overestimation in the revised manuscript (p. 8, lines 12 - 13).

In response to the reviewer's insightful suggestion, we analyzed the co-localization of Centrin2 (a centriole marker) with γ-tubulin (a PCM marker) or PHH3 (phosphorylated histone H3; a mitotic marker) in MEFs derived from Cep152$^{W105*/K897*}$ and Cep152$^{Q32P/Q32P}$ mice to examine the centriole integrity. The results obtained were shown in Fig 4D - G in the revised manuscript, with the corresponding description added to the Materials and Methods and Results sections (p. 22, lines 27 – 29; p. 11, lines 18 -25). Furthermore, we performed electron microscopy on VZ progenitor cells in cortical slices to examine potential defects in centriole architecture. These findings are shown in Fig. 5 of the revised manuscript, and the corresponding description has been added to the Materials and Methods (p. 25, line 11 - p. 26, line 2) and Results sections (p. 13, lines 4 - 26).

2) Expression and stability of the pathological variants are investigated in a normal context in N2A cells, meaning in the presence of a normal version of CEP152. The patients exhibit only pathological versions of CEP152 in their genome. If the authors perform siRNA against

CEP152 to remove the normal version of the protein in N2A cells and perform the same investigation regarding pathological variant localization, stability and centriole/centrosome architecture, do they obtain the same results?

Response: We appreciate the reviewer's insightful comment that expression of the pathological variants should be analyzed in the absence of endogenous wild-type CEP152. Since we believe that analyses using knock-in mouse models, which closely mimic the patients' pathophysiological conditions, provide more directly relevant insights than studies in N2A cells, we examined the expression and subcellular localization of endogenous CEP152 variant proteins in VZ progenitor cells of $Cep152^{W105*/K897*}$ and $Cep152^{Q32P/Q32P}$ mice, as well as in MEFs derived from these mice. To this end, we used an anti-CEP152-N, which targets the N-terminal region of the protein (Genetex Cat# GTX128027), as anti-CEP152-C is not useful because the p.K897* variant lacks its C-terminal epitope region. Immunofluorescence analyses revealed that CEP152-K897* is predominantly localized in the cytoplasm rather than at the centrosome, whereas CEP152-Q32P is localized at the centrosome as in the case of wild-type CEP152 both in cortical slices and MEFs. We included these results in Fig. 2G and H in the revised manuscript. Notably, these results are similar to those in the overexpression analyses using N2A cells and migrating cortical neurons (Fig. 2B and C). The corresponding description has been added to the Results section (p. 9, lines 6–13) and the figure legend.

3) The authors need to investigate any defects in the localization of CEP152 partner proteins in the mouse mutant brains and more importantly centrosome (centriole and PCM) and cilium architecture. Indeed, the enlargement of ventricular area observed in Figure 3A-C could be related to defects in motile cilia at the surface of ependymal cells as a consequence of impaired circulation of the cerebrospinal fluid. Such defects in cilia could be due to defects in centriole architecture in the CEP152 Q32P/Q32P mutant brain, which has not been explored. If the case, centriole defects should be different in the W105/K897 mutant brain, as no ventricular enlargement can be observed.

Response: We appreciate the reviewer's thoughtful comment. Because the commonly used anti-PLK4 antibodies (Proteintech Cat#12952-1-AP; Santa Cruz Cat#sc-100413) didn't stain the centrosomes in either MEFs or cortical slices. We instead tested CPAP (also known as CENPJ/SAS-4; Proteintech Cat#11517-1-AP), a known CEP152-binding partner. Although the manufacturer's protocol indicates that this antibody is suitable for immunocytochemistry, it failed to detect CPAP in MEFs. We also considered analyzing CEP192, another CEP152-

interacting protein; however, suitable antibodies are not available for mouse CEP192, as currently available reagents were raised against the human protein, which differs considerably in amino acid sequence from the mouse orthologue.

According to the reviewer's comment, we analyzed the number of motile cilia by counting the basal bodies of ependymal cells at P9. The number of motile cilia was reduced in both models, with a more severe reduction observed in Cep152$^{Q32P/Q32P}$ mice. This more pronounced loss of motile cilia in Cep152$^{Q32P/Q32P}$ mice may lead to severely impaired cerebrospinal fluid circulation and, consequently, ventricular enlargement. These results have been added as Fig. EV2D and E, and the corresponding description has been incorporated into the revised manuscript (p. 9, lines 16 – 25).

We also investigated the primary cilia of VZ progenitor cells by staining for Arl13b, a ciliary membrane marker. We stained cortical slices (E15.5) with anti-Arl13b and analyzed the length and number of them. As a result, the primary cilia appeared morphologically normal in Cep152$^{Q32P/Q32P}$ mice, whereas their number was reduced (Fig. EV4A-C in the revised manuscript). The corresponding descriptions have been added to the Results section (p. 12, lines 25 - 31) and figure legend.

4) Lines 30-32, page 9, the authors propose that as cell death is most prevalent in the IZ, it primarily occurred in differentiated neurons. The position of dying cells in the neuroepithelium is not sufficient to conclude on dying cells identity as progenitor cells always detach before undergoing apoptosis as described in the literature. Therefore, if the authors want to conclude on the identity of dying cells, they should perform a double staining with the tubulin ßIII marker for example.

Response: We agree with the reviewer's comment. We double stained E15.5 cortical sections from WT and Cep152$^{Q32P/Q32P}$ mice with anti-aCasp3 together with anti-Tbr2 (a basal progenitor marker) or anti–βIII-tubulin (a neuronal marker). The results are shown in Fig. 4K of the revised manuscript. The corresponding descriptions have been added to the Results section (p. 12, lines 6 - 10) and figure legend.

5) Apoptosis and premature neuronal differentiation have been shown as consequences of mitotic defects in microcephaly brain models. The authors have reported apoptosis in their CEP152 mutant brains. As they propose that differentiated neurons are the ones dying, there is a possibility that progenitors having experienced mitotic defects exit prematurely the cell cycle to

undergo neuronal differentiation before dying. If the case, they might see a decrease in the TBR2-positive basal progenitor population and an increase in the tubulin ßIII-positive population. The authors need to explore this possibility.

Response: We appreciate the reviewer's insightful comment. In response, we performed immunostaining on E15.5 cortical sections from WT, Cep152$^{W105*/K897*}$, and Cep152$^{Q32P/Q32P}$ embryos using an anti-Tbr2 antibody (basal progenitor marker) and an anti–βIII-tubulin antibody (neuronal marker). Quantitative analysis revealed a significant reduction in the Tbr2-positive basal progenitor population in the mutant cortices (Fig. EV3E and F), supporting the possibility that mitotic defects may impair basal progenitor maintenance. We have added these data in the revised manuscript (p. 10, lines 10 - 13). In contrast, we were unable to reliably quantify the βIII-tubulin–positive neuronal population, as βIII-tubulin is broadly expressed throughout the neuronal cytoplasm, including axons and dendrites, which precludes accurate identification and counting of individual neuronal cell bodies. We displayed the image of βIII-tubulin staining below. Another neuronal marker NeuN was not suitable as an alternative neuronal marker because it is not expressed at this developmental stage.

By contrast, in Fig. 4K (p. 12, lines 6 - 10) of the revised manuscript, quantification was feasible because βIII-tubulin was co-stained with aCasp3, allowing unambiguous identification of dying neurons. In addition, apoptotic neurons exhibited relatively a rounded morphology, further facilitating reliable cell counting.

We hope this additional analysis and explanation adequately address the reviewer's concern.

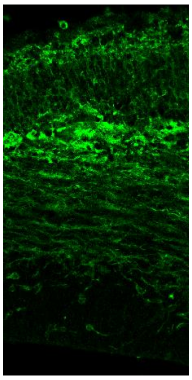

6) Defects in the number of mitotic foci indeed reflect defects in centrosome number and are typically analysed in mitotic cells, and prometaphase especially. Nonetheless, the number of gamma-tubulin foci does not enable to investigate centrosome integrity defects, as PCM can organize in the absence of centrioles. Can the authors check the presence and number of centrioles at each pole after a centrin staining?

Response: We appreciate the reviewer's comment. We stained centrioles using anti-Centrin2 together with either anti-γ-tubulin or anti-pHH3 in MEFs from each genotype. The results are shown in Fig. 4D - G of the revised manuscript. The corresponding description has been added to the Results section (p. 11, lines 18–25) and to the figure legends.

In addition, we performed electron microscopy to examine centriole structure. The resulting data have been included in Fig. 5 of the revised manuscript, and the corresponding descriptions have been added to the Materials and Methods (p. 25, line 11 - p. 26, line 2) and Results sections (p. 13, lines 4 - 26).

7) Centriole loss after CPAP removal (another centriole protein) results in primary cilium loss, radial glia cell detachment and massive heterotopia as a consequence of neuroepithelial junction disorganization. As the authors report some tissue disorganization in the cerebral cortex and cerebellum in their Q32P/Q32P homozygous mutant model, can it also result from similar (even if milder) developmental defects in the neuroepithelium?

Response: We appreciate the reviewer's insightful comment. To investigate whether the tissue disorganization in Cep152$^{Q32P/Q32P}$ mice might result from developmental defects in the VZ, we analyzed the morphology of primary cilia in VZ progenitor cells (E15) by immunostaining with anti-Arl13b. Notably, although the morphology of primary cilia appeared normal in the Q32P model, their number was reduced (Fig. EV4A–C). This decrease likely reflects the reduced number of progenitor cells. Additionally, the localization of β-catenin (a marker of adherens junctions) in the VZ of the mutant brains was comparable to that in wild-type brains (Fig. EV4D and E). The morphology of radial glial fibers also appeared preserved when nestin was stained (Fig. EV4F and G). These findings suggest that centriole defects in these mice do not lead to structural defects in primary cilia, radial glial detachment, or disorganization of neuronal junctions. The corresponding description has been added to the Results section (p. 12, line 25– p. 13, line 3) with the figure legend updated accordingly.

Minor points:

1) Can the authors provide higher magnifications of centrosomes in Fig2B?

Response: Following the reviewer's suggestion, we have shown higher magnification images of Fig. 2B in the revised manuscript.

2) In figure 4, panel G reporting the % of mitotic cells should be placed in D, as it is in the middle of the % of apoptotic cell analysis.

Response: We appreciate the reviewer's suggestion regarding the panel arrangement. In the original version of Fig. 4, panels A–F focus on mitosis and apoptosis in the cerebral cortex, whereas panels G and H present analyses performed in the cerebellum. To preserve this anatomical and thematic separation, we prefer to retain the current panel order. In the revised manuscript, we have added representative images corresponding to panels G and H from the initial manuscript. We hope this explanation clarifies our rationale and adequately addresses the reviewer's concern.

3) In figure 4, can the authors better explain in the figure legend which region is analysed in panels E and H.

Response: We analyzed the entire cerebral cortex for the quantification shown in Fig. 4E in the original version. In contrast, the quantification in Fig. 4H was performed using the external and internal granule layers of the cerebellum. In accordance with the reviewer's suggestion, we have revised the figure legend (Fig. 4I and O in the revised manuscript) to clearly specify the anatomical regions analyzed in these panels. We hope this clarification satisfactorily addresses the reviewer's comment.

4) The authors record different defects in neuron excitability in their two CEP152 mutant brains. Can they discuss more these observations in the discussion section, do they have any explanation regarding the differences observed?

Response: We appreciate the reviewer's insightful comment. We tried to make discussion on the different defects in neuron excitability in the two CEP152 mutant brains in the revised manuscript (p. 18, lines 9 - 26).

Referee #2 (Remarks for Author):

Major points regarding the human phenotype:

The main concern is whether or not there is severe cerebral or cortical malformations defined by the authors as agenesis of the corpus callosum, polymicrogyria, ventricular dilatation, interhemispheric cyst, associated with microcephaly. The information provided by the authors does not allow this to be confirmed:

i) On axial images (figure 1C), the corpus callosum appears to exist anteriorly in patients 2 and 3, coronal and strict sagittal images are required.

Response: We thank the reviewer for the comment. We have now modified the text to read "hypogenesis of the corpus callosum" after the MRI images were carefully examined and consultation with a neuroradiology expert. Please see below:

Patient 2:

Sagittal MRI showing severe hypogynesis of the corpus callosum, the arrow indicates the

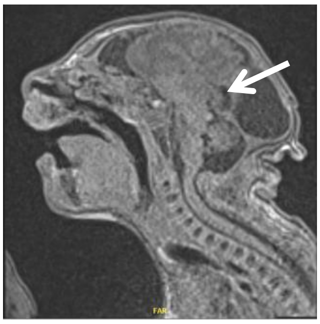

splenium. We revised the image as Fig. 1C upper middle panel and amended the text (p. 6, lines 11-12) and figure legend in the revised manuscript.

Patient 3:

Axial and sagittal MRI shows hypogenesis of the body and splenium of the corpus callosum as indicated by arrows: left and right arrows show hypogenesis of the splenium and body of the corpus callosum, respectively. We added these MRI data in the revised manuscript as Fig. EV 1F and described these results (p. 6, lines 32 - 33) and figure legend in the revised manuscript.

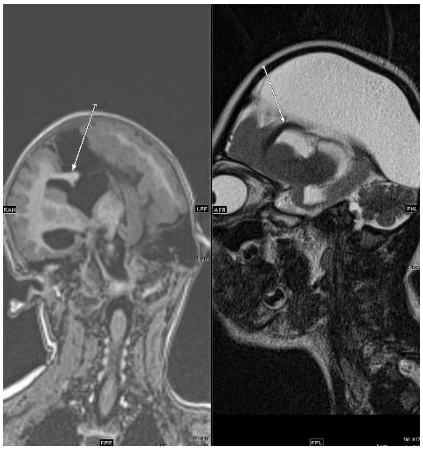

ii) According to the results and Figure 1C, Patient 2 has no polymicrogyria, and the polymicrogyria claimed by the authors for patients 3 carrying the same variants (c.95A>C) is not obvious on the MRI images (figure 1C); an axial and coronal T1-weight sequence is

essential to make a conclusion. This aspect resembles microcephaly with a very simplified gyration.

Response: We appreciate the reviewer's comment. After consultation with a neuroradiologist, we have amended the text and the table because there is no evidence of polymicrogyria in Patient #2. However, Patient #3 does exhibit polymicrogyria, as shown by the axial T1-weighted MRI image provided (Fig. EV1G in the revised manuscript). A corresponding description has also been included (p. 6, line 33 - p. 7, line 1).

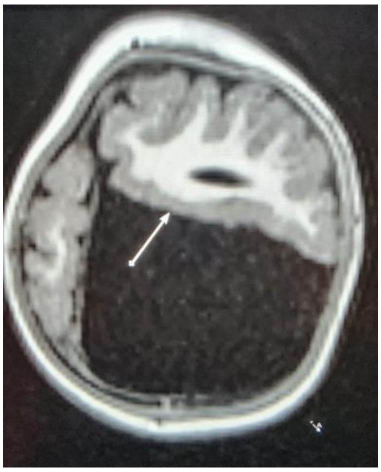

iii) Ventricular dilatation may be secondary to an obstacle (is the aqueduct of sylvius permeable?), or may reflect insufficient growth (a vacuo dilatation), but in both cases, this is not classified as a cerebral malformation by the international classifications.

iv) Interhemispheric cyst is not a cerebral malformation either, as it is extracerebral and has developed at the expense of the arachnoid.

Without these additional imaging data, it seems impossible to affirm that patients 2 and 3 have a cerebral or cortical malformations associated with their microcephaly. Microcephaly with very simplified gyration and interhemispheric cyst +/- hypoplasia of the corpus callosum would be more appropriate, and this should be modified in the title, summary, results and discussion. If the OFC of patient 2 and 3 were < -10 SD, the term micrencephaly could be used.

Response: We thank the reviewer for the comment. We have now amended the text and replaced "brain malformation" with "severe microcephaly with marked gyral simplification" regarding to the Patient #2 (p. 6, line 22 in the revised text). The OFC of Patient#2 is more than -10 SD, not meeting the criteria of "micrencephaly," whereas that of Patient #3 is -12 SD. Based on the findings obtained, we modified the title as "Distinct pathophysiological mechanisms of *CEP152* variants in microcephaly and brain abnormalities" in the revised version. We hope these amendments satisfactorily addresse the reviewer's comments.

Major points regarding cell biology in vitro experiments or in vivo models:

Several data raise questions:

1. Do the Cep152 Q32P/Q32P mutant have a cortical and cerebellar malformations?

P8 lines 14-16: what is this "abnormal depression of the cortical surface"? This work would be greatly improved if the authors could well characterize the cortical plate of these Q32P/Q32P mutant mice by showing images of the whole brain and images of the cortical plate at objective 40 in Figure 3 or a supplementary figure for the 9 brains that have been analysed. It is important to compare brain structure of mouse and human brains mutated for CEP152 to verify the adequacy of the model.

Response: We appreciate the reviewer's insightful comment. By "abnormal depression of the cortical surface," we refer to localized indentations on the cortical surface, resembling shallow grooves or dimples, which are observed at neonatal period but are no longer detectable by P10 (Fig. 3E and Fig. EV3C). We described this in the revised manuscript (p. 10, lines 4 - 6). We have further characterized the Cep152$^{Q32P/Q32P}$ brains by providing images of the whole brain as well as high-magnification images of the cortical plate (objective 40×). These data are now included as Fig. EV2B, C, F and Fig. EV3C, D in the revised manuscript (p. 9, lines 16 – 18, lines 25 - 27; p. 10, lines 4 – 6, line 9). Cep152$^{Q32P/Q32P}$ mice also recapitulate the patient's phenotype such as ventricular enlargement at 6 months (Fig. EV 2C in the revised manuscript).

2. On Figure 3 A and B, corpus callosum is present. It is important to mention this in the results and to compare it with brain MRI images of patients.

Response: We appreciate the reviewer's comment. Hypogenesis of the corpus callosum was observed in both mouse models at P60, with a more pronounced phenotype in Cep152$^{Q32P/Q32P}$ mice (Fig. EV2F in the revised manuscript). We mentioned the size of the corpus callosum in WT, Cep152$^{W105*/K897*}$ and Cep152$^{Q32P/Q32P}$ mice, and compared them with brain MRI images of patients in the revised manuscript (p. 9, lines 25 - 27).

3. P9 lines 1-3 and Figure 3M: "Dentritic development of Purkinje cells also appeared impaired in Q32P/Q32P mice", however, dendritic cells seem to be increased. It would be important to quantify this at P6 and to and then try to interpret this result with the apoptosis at P9.

Response: Following the reviewer's suggestion, we quantified localization and number of Purkinje cells at P6 (Fig. 3P-R in the revised manuscript). While the number of immature

Purkinje cells increased at P6 (Fig. 3R), these cell number reduced thereafter at P60 (Fig. 3M and O in the revised manuscript). Notably, Purkinje cells were ectopically distributed at P60 (Fig. 3N). We described this interpretation in the revised manuscript (p. 10, line 21; lines 26 - 30).

4.What is the structure of the centrosome after overexpression of the variants in vitro or in Ki mutant mice in vivo?

"The variants do not affect the structure of the centrosome", however the experiments shown in figure 2 and in Results (paragraph "biological and biochemical properties of the CEP152 variants ...." and γtubulin staining alone) are not sufficient to affirm that centrosome structure is not affected. Knowing the role of CEP152 in centriole biogenesis, this work would be greatly improved if the authors could provide data to support their hypothesis by investigating the centrosome using centriole staining, high magnification and high resolution microscopy.

Response: We appreciate the reviewer's insightful comment. In response, we further investigated the effects of these variants on centriole organization. To this end, we analyzed the co-localization of Centrin2 (a centriole marker) with γ-tubulin (a PCM marker) or pHH3 (a mitotic marker) in MEFs derived from $Cep152^{W105*/K897*}$ and $Cep152^{Q32P/Q32P}$ mice. The results obtained were shown in Fig 4D - G in the revised manuscript, with the corresponding description added to the Results section (p. 11, lines 18 - 25). Furthermore, to analyze the effects of the variants on the centriole and centrosome structures, we performed electron microscopy on VZ progenitor cells in cortical slices to analyze the effects of the variants on centriole architecture. These findings are shown in Fig. 5 of the revised manuscript, and the corresponding descriptions have been added to the Materials and Methods (p. 25, line 11 - p. 26, line 2) and Results sections (p. 13, lines 4 - 26).

5. The authors show an increase in the proportion of cells in mitosis with a single point of gamma tubulin and an increased proportion of cells in prophase/prometaphase and conclude that there is a high proportion of monopolar spindles.

i) Could the authors show these images in 3D to demonstrate whether these cells have a monopolar spindle in only one side of the metaphase plate or are almost normal prophase with a single centrosome instead of two?

Response: According to the reviewer's suggestion, we tried to demonstrate whether the mitotic cells with a single point of γ-tubulin have a monopolar spindle in only one side of the

metaphase plate or are in prophase with a single centrosome instead of two. To this end, we performed double immunostaining of VZ progenitors from Cep152$^{W105*/K897*}$ and Cep152$^{Q32P/Q32P}$ genotypes using anti–α-tubulin and anti–γ-tubulin antibodies. We then quantified the proportion of cells with a single centrosome in prophase and those with a monopolar spindle in metaphase/anaphase. These results have been shown in Fig. EV3G - I in the revised manuscript. The corresponding description has also been incorporated into the Results section (p. 11, lines 12–18).

ii) "p9 line 22: "These finding indicate a prolonged pro/prometaphase duration in mitotic progenitors". Unless authors film the cells using live-microscopy to measure mitosis duration, this can't be affirmed. This experiment would be important and show the outcome of these cells.

Response: We agree with the reviewer’s concern that the duration of mitotic phases cannot be determined without live-cell imaging. Since Cep152$^{W105*/K897*}$ and Cep152$^{Q32P/Q32P}$ mice are born at sub-Mendelian ratios, collecting a sufficient number of embryos for live imaging requires a considerable amount of time. Therefore, we have deleted this sentence in the revised manuscript. We hope the reviewer will kindly accept this explanation.

iii) P10 lines 1-3: "these results demonstrate that CEP152 is critical for maintaining centrosome integrity and mitotic fidelity". Experiments are lacking to affirm this since i) only the W105* mutant shows the absence of CEP152, ii) centrosome integrity has not been clearly demonstrated here, iii) images of monopolar spindles have not yet been shown in 3D.

Response: We agree with the reviewer’s concerns and suggestions. To strengthen our conclusions, we co-immunostained MEFs derived from the mouse models with anti-Centrin2 together with anti-γ-tubulin or anti-PHH3 to examine the centriole integrity. As a result, both mutant cell types displayed a reduced number of centrioles, with the defects being more pronounced in Cep152$^{Q32P/Q32P}$ mice (Fig. 4D - G in the revised manuscript). We then confirmed defects in centriole structure by electron microscopy (Fig. 5 in the revised manuscript). In addition, we assessed monopolar spindle formation in mitotic neural progenitors by double immunostaining with anti-α-tubulin and anti-γ-tubulin. This analysis revealed that cells with an abnormal single γ-tubulin focus were observed both in prophase and in meta/anaphase (Fig. EV3G - I in the revised manuscript). The corresponding descriptions have been added to the

Results and Materials and Methods sections in the revised manuscript (p. 11, lines 12 - 25; p.13, lines 4– 26; p.25, line 11– p.26, line 2).

Specific comments:

Introduction

1) P4 lines 1-7: Seckel syndrome is characterized by proportionate short stature or primordial dwarfism, associated with an extreme smallness of the head, with a peculiar aspect resembling "bird-headed", and is related to PCNT variants. The subgroup of patients with CEP152 variants do not exhibit proportionate dwarfism and microcephaly (height and OFC were not equally reduced), as expected in patients with Seckel syndrome. Prefer here "microcephalic primordial dwarfism".

Response: According to the reviewer's suggestion, we revised the manuscript and the subgroup of patients with CEP152 variants was referred to as "microcephalic primordial dwarfism" in the revised manuscript (p. 4, line 26 - p. 5, line 2).

2) P4 line 14: Please change severe or multiple brain malformations for severe microcephaly with marked gyral simplification or micrencephaly (see above)

Response: According to the reviewer's suggestion, we rewrote the sentence in the revised manuscript (p. 5, lines 9 - 10). We also amended the Abstract in the revised manuscript.

Results

3) Developmental stages of mice should be indicated in the text to help the reader to understand the altered developmental processes. This must also be added in all the figures

Response: As suggested by the reviewer, we have added the developmental stages of the mice throughout the main text to clarify the timing of the observed phenotypes. In addition, we have included the corresponding developmental stages in all relevant figure panels in the revised manuscript.

4) P5 line 11: NICU: please define

Response: We have defined the term in the revised manuscript (p. 6, line 9).

5) P5 line 19: "bilateral intraocular detachment, consistent with retinal detachment": This could be a new phenotype related to CEP152, CT-scan n Figure 3 A and B must be shown / added in Figure 1 to affirm this sentence.

Response: We thank the reviewer for this comment. We have now added the CT-scan and MRI images showing the retinal detachment for Patient #2 (Fig. EV1E in the revised manuscript) and described the results in the revised manuscript (p.6, lines 15-17).

To analyze the eye phenotypes in the mutant mice, we consulted Prof. Akira Hara (Department of Pathology, Gifu University Graduate School of Medicine, Japan), a long-term collaborator with extensive experience in ocular research. Prof. Hara has published numerous studies on eye pathology and emphasized that preparation of intact mouse eye samples is technically challenging, as retinal detachment frequently occurs during normal tissue processing. He noted that, in his own analyses, he typically focuses on retinal regions that remain attached after preparation. Based on his experience, he cautioned that it would be difficult to determine whether the retinal detachment observed in $Cep152^{Q32P/Q32P}$ mice is genuinely caused by CEP152 deficiency or represents an artifact introduced during sample preparation. We therefore respectfully ask the reviewer to consider this inherent technical limitation of the current analysis.

6) P5 line 29: patient 3 is blind. Could the authors explain why? Is there ophthalmological examination or eye imaging available?

Response: We thank the reviewer for this insightful comment. Unfortunately, ophthalmological imaging was not available, as we were unable to obtain the examination images. Only the written ophthalmology report was accessible in the patient’s medical chart, which stated that Patient #3 was blind due to bilateral retinal detachment associated with persistent hyperplastic primary vitreous (PHPV). We described this issue in the revised manuscript (p.7, lines 3 - 4).

7) P5 line 32-33: photographs of patient 3 must be shown in supplementary figure 2.

Response: We thank the reviewer for this comment. Unfortunately, we were unable to obtain consent from the parents to publish the photographs in the manuscript. Therefore, we are not able to include the images in the revised manuscript. We respectfully ask the reviewer to accept this limitation.

8) P6 line 3-4: ERG and VEP should be added in supplementary figure 2.

Response: We thank the reviewer for this comment. Unfortunately, ERG and VEP examination

images were not available, as we were unable to obtain the original ophthalmological imaging data. Only the written ophthalmology report was accessible in the patient's medical chart; therefore, these examinations cannot be included in Fig. EV2. We respectfully ask the reviewer to accept this limitation.

9) P7 line1: in Guernsey' article, it's CEP152-R987* and not -R986*, please verify.

Response: We are sorry for this simple mistake. Following the reviewer's suggestion, we corrected the typo in the revised manuscript (p. 8, line 3).

10) P8 line 9: "this reduction was more pronounced in the superficial layers compared to the deep layers": what statistical test did the authors perform to compare both variables, groups and superficial vs deep layers thickness, and affirm this sentence and the following one? This is not mentioned in the methods or the legend to figure 3.

Response: We thank the reviewer for this important comment. The original statement ("this reduction was more pronounced in the superficial layers compared to the deep layers") could not be statistically supported using Fig. 3F (Fig. 3E in the original manuscript) alone, as Fig. 3F presents the absolute thickness of each cortical layer and does not allow a direct statistical comparison between superficial and deep layers. To address the reviewer's concern, we have now added a new analysis (Fig. 3G in the revised manuscript) in which we calculated the ratio of layer II-IV and layer V-VI, enabling appropriate statistical comparison between superficial (II-IV) and deep (V-VI) layers. The corresponding descriptions, including details of statistical analyses, have been added to the figure legend. We hope this additional analysis sufficiently addresses the reviewer's question.

11) P8 line 18 and Suppl figure 5C: What is the yellow coloration? It is not explained in the results or on the figure legend. The cerebrum and cerebellum structure seem to be respected as shown in Figure 3 DFH. Could the authors explain this?

P8 -P9: see above

Response: The yellow coloration observed in Fig. EV3D in the revised manuscript (originally Supple Fig. 5C) is not a biological feature but results from the filter characteristics of the fluorescence stereomicroscope used to capture the images. As this coloration is an artifact of the imaging system, the interpretation should be based on the morphological features rather than the color itself. We have now clarified this point in the legend for Fig. EV3D. We also added

histological and immunohistochemical data demonstrating the cerebral and cerebellar structures in the revised manuscript (Fig. EV2B, C, and F and Fig. EV3C and D).

12) P9 lines 30-32: "Although cell death ... was most prevalent in the IZ, suggesting that it primarily occurred in differentiated neurons rather than in progenitors". It is important to demonstrate this hypothesis by a double immunostaining.

Response: We agree with the reviewer's comment. As suggested, we performed double immunostaining of E15.5 cortical slices from Cep152$^{Q32P/Q32P}$ mice using anti–aCasp3 together with either anti–βIII-tubulin (a neuronal marker) or anti–Tbr2 (a basal progenitor marker). Apoptotic cells were detected in both populations, with a greater number observed among neurons. These results are presented in Fig. 4K of the revised manuscript. Accordingly, we have amended the corresponding text in the Results section (p. 12, lines 6 – 10) as well as the figure legend in the revised manuscript.

13) P10 line 19: "cerebellar hypoplasia and malformations": which malformations? There is no obvious malformation in Figure 3.

Response: As the reviewer correctly pointed out, we deleted the word "malformation" from the text in the revised manuscript (p. 12, line 23).

14) P11 lines 8-10: Could this decrease of dendritic arborisation be due to the reduction of neuronal density?

Response: We thank the reviewer for this insightful comment. In the revised manuscript, we presented data showing that both the granular and molecular layers are thinner at P60 in Cep152$^{Q32P/Q32P}$ mice (Fig. EV4H-J). This thinning is most likely to reflect reduced neuronal density, which may decrease synaptic input to Purkinje cells. Such a reduction in input could plausibly contribute to the observed impairment in dendritic arborization. Consistent with this idea, quantification of Purkinje cell numbers per unit area revealed a decrease in cell density in Cep152$^{Q32P/Q32P}$ mice at P60 (Fig. 3M and O in the revised manuscript). However, we currently do not have direct evidence demonstrating that reduced neuronal density is the causal factor underlying the diminished dendritic arborization. We therefore interpret this as a possible, but not yet proven, contributing mechanism. We hope that this clarification adequately addresses the reviewer's question.

15) P11 lines 26-30: Do the authors suggest changes in the excitatory / inhibitory balance? Could the authors confirm this hypothesis by showing this in IF?

Response: We appreciate the reviewer's thoughtful comment. We agree that our findings suggest alterations in the excitatory/inhibitory (E/I) balance in Cep152$^{W105*/K897*}$ mice. In this model, we observed a selective increase in mEPSC amplitude, whereas both the frequency and amplitude of mIPSCs remained unchanged, indicating a shift toward enhanced excitatory synaptic drive. In contrast, Cep152$^{Q32P/Q32P}$ mice exhibited reductions in both mEPSC and mIPSC frequencies and amplitudes, suggesting a broad impairment in synaptic function rather than a specific alteration of the E/I balance. We have revised the text accordingly and now explicitly refer to E/I balance in the revised manuscript (p. 14, line 29 – p. 15, line 2). To further assess this possibility, we attempted immunostaining of cortical slices from Cep152$^{W105*/K897*}$ mice using an anti-AMPA receptor antibody (Abcam, Cat# ab31232); however, the staining patterns were nonspecific, and we were unable to obtain interpretable data. We hope this explanation satisfactorily addresses the reviewer's concern.

Discussion

16) P14 lines 6-8 and p15 line 28: reword this sentence, in particular multiple cerebral malformations, unless these malformations are shown more clearly on MRI in patients 2 and 3 (see above)

Response: We thank the reviewer for this helpful comment. As shown in the new MRI image (Fig. EV1G), we confirmed that Patient #3 indeed has polymicrogyria. Accordingly, we have revised the relevant text while retaining the term "polymicrogyria" in the revised manuscript (p. 17, lines 9 - 11). In addition, we have amended the wording on p.15, line 28 of the initial manuscript (p. 19, line 13 in the revised manuscript). We hope that this amendment satisfactorily addresses the reviewer's concern.

17) P14 lines 13-14: ID is present in many MOPDII patients, this is not an argument.

Response: We agree with the reviewer and have deleted the relevant statement from the manuscript (originally p. 14, lines 13–14). This change is reflected in the revised manuscript (p. 17, lines 16 – 17).

18) P14 lines 25-27: Please rephrase after taking into account new experiments aimed at better characterising the structure of the centrosome

Response: In line with the reviewer's suggestion, the sentence has been rephrased to reflect the results of the additional analyses aimed at better characterizing the centrosome structure (p. 17, lines 27– 31).

19) P14 lines 32-34: Could the authors explain more the arguments in favor of this hypothesis?

Response: We appreciate the reviewer's constructive feedback and have provided a more detailed rationale in support of this hypothesis in the revised manuscript (p. 17, line 31 - p. 18, line 8; p. 18, lines 28 - 30).

20) P15 lines 1-8: Could the authors also discuss the possibility of an imbalance between excitatory and inhibitory neurons?

Response: We thank the reviewer for this comment. We referred to this issue in the revised manuscript (p. 18, lines 9 – 26). We hope this addition satisfactorily addresses the reviewer's comment.

21) P15 lines 27-34: to comment on the results of transcriptomic data, could the authors replace at what stage the RNASeq has been performed (P60) and the developmental processes that are still ongoing or maintained at that stage?

Response: We appreciate the reviewer's comment. In the revised manuscript, we have clarified that RNA-Seq was performed on cortical tissue at P60, a stage when neuronal circuit maturation and synaptic remodeling are still ongoing. These biological contexts are important for interpreting the transcriptomic changes observed. We have added a description of these developmental processes to better contextualize the transcriptomic findings (p.19, lines 9 - 11 in the revised manuscript). We hope that this clarification addresses the reviewer's concern.

Methods

Precise the statistical tests that were used (see above) and the imaging protocol for mitosis experiments

Figures and Tables

1) Figures 2-8: developmental stages of mice should be indicated in each figure to help the reader to understand the altered developmental processes. This should also be added in the results

Response: We appreciate the reviewer's comment. We indicated developmental stages of mice in each figure panel and the Results section in the revised manuscript.

2) Figure 2: Show the whole blot for Myc-CEP152-W105*

Response: We added the whole blot for the data in Fig. 2A as Fig. EV5A in the revised manuscript.

3) Please enlarge Fig 2C

Response: According to the reviewer's suggestion, we enlarged Fig. 2C in the revised manuscript.

4) Figure 3E: Precise what statistical tests were used

Response: According to the reviewer's comment, we clarified statistical tests used for Fig. 3F (Fig. 3E in the original manuscript) in the legend of the revised manuscript.

5) Figure 4A: add a 3D image of monopolar spindles

Response: In response to the reviewer's suggestion, we determined whether the cells with a single centrosome are in the meta/anaphase with a monopolar spindle or in the prophase. To this end, we conducted double immunostaining analysis with α-tubulin and γ-tubulin antibodies. We then calculated the proportion of cells with a single centrosome in the prophase and cells in the meta/anaphase with a single centrosome. This analysis revealed that cells with a single γ-tubulin focus were observed both in prophase and in meta/anaphase. We added the data in Fig. EV3G and H in the revised manuscript. Representative serial images of a monopolar spindle were also shown (Fig. EV3I). We have also added the corresponding description to the Results section (p. 11, lines 12–18). We hope the additional data satisfactorily address the reviewer's concern.

6) I did not find Table 1. Is it Supplementary Table 1?

Response: We apologize for the confusion. The intended reference was to Table EV1. The supplementary materials include four tables in total.

7) Supplementary Figure 2:

- There is too many OFC values for patient 1 between age 6 and 10y that makes the OFC curve confusing. This should be clarified.

- Add OFC values of patients 2 and 3 to the OFC curve of patient 1

Response: We appreciate the reviewer's comments. In response to the reviewer's suggestions, we revised the Supplementary Fig. 2A in the initial manuscript to improve clarity. Specifically, in Fig. EV1A in the revised manuscript, we reconstructed the OFC curve for Patient #1 (male) by reducing the number of plotted data points between ages 6 and 10 years, thereby avoiding overcrowding in this age range. In addition, we added the OFC measurement of Patient #3 (male) at age seven to the figure. With regard to Patient #2 (female), the OFC value at birth was below the lower limit of the chart scale for girls as shown below; therefore, it could not be plotted in the figure and is described only in the revised text (p. 6, lines 2 – 4). We hope this revision adequately addresses the reviewer's concern.

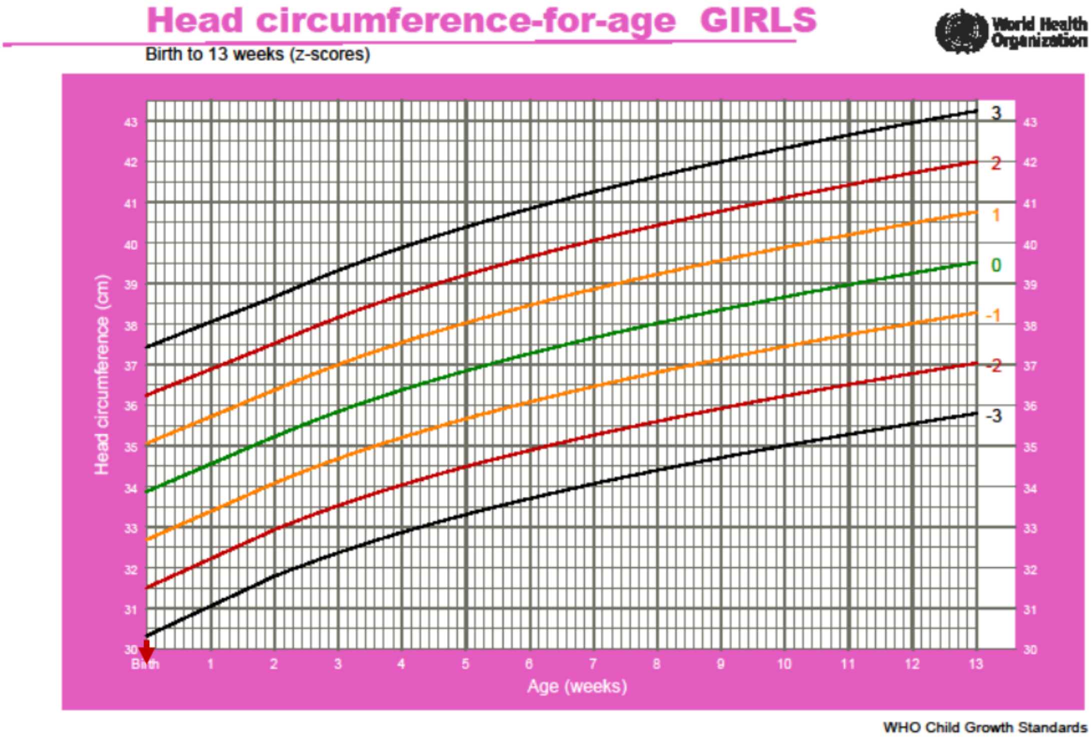

- Add photographs and ERG/VEP of patient 3

Response: We thank the reviewer for this comment. Unfortunately, we were unable to obtain the ophthalmological examination images for Patient #3. Only a written report was available in the patient's medical chart, and therefore the requested photographs and ERG/VEP data cannot be included. We respectfully ask the reviewer to accept this limitation.

8) Supplementary Figure 6A: I would point out to the authors that the selected image shows a lagging chromosome in the WT

Response: We appreciate the comment. We realized that we had unintentionally chosen an atypical cell for the original image, and have now replaced it with one that more accurately represents the typical phenotype (Fig. EV3J in the revised manuscript).

Supplementary Table 1:

9) Supplementary Table 1 is incomplete. At least 3 previously reported are lacking. This should be updated. Values of OFC and height of patients (at least in SD) should be added to define the severity of microcephaly, characterize short stature as primordial dwarfism or not and to state that one patient is more severe than another.

Response: We thank the reviewer for this important comment. As suggested, we have updated Table EV1 to include the previously reported cases that were initially missing. We have also added OFC and height values (expressed in standard deviation scores, where available) for all patients, including those in our study and previously reported cases. These additions allow a more precise assessment of microcephaly severity, a better characterization of short stature in relation to primordial dwarfism, and clearer comparison among patients regarding phenotypic severity.

10) The references should be written in the title of the columns and cited below the table as bibliographic references

Please correct the spelling errors

Response: We appreciate the reviewer's helpful suggestion. As recommended, we have added the corresponding reference numbers or author names to the column headers in Table EV1 to clearly indicate the source of each previously reported case. In addition, we have listed the full bibliographic references below the table for clarity and proper citation. We also checked spelling errors.

References

P3 line 11: the first 3 references do not concern NDDs

Response: We agree with the reviewer's comment and have deleted the first 3 references.

Referee #3 (Remarks for Author):

Major points:

1. In Fig. 2A, the authors overexpress CEP152-WT and its variants in N2A cells, concluding that the expression levels of the mutants are comparable to CEP152-WT. However, since expression levels can vary between vectors, it is essential to examine and quantify endogenous CEP152 levels in the brains of the two knock-in mouse models using immunoblotting. Additionally, the altered localization of CEP152 variants observed in Fig. 2B should be validated using mouse embryonic fibroblast (MEF) cells derived from the knock-in models to confirm the findings in a physiologically relevant context.

Response: We appreciate the reviewer's insightful comment. In response, we examined endogenous CEP152 levels in the brains of Cep152$^{W105*/K897*}$ and Cep152$^{Q32P/Q32P}$ mice by immunoblotting. We previously generated an anti-CEP152-C antibody targeting the C-terminal region (Hamada et al., Dev. Neurosci. 44, 162–170, 2022. DOI:10.1159/000523922). Western blot analysis using this antibody showed that endogenous CEP152-Q32P protein levels in Cep152$^{Q32P/Q32P}$ mice were comparable to wild-type CEP152 in littermates. These results are presented in Fig. EV2A, and the corresponding description has been added to the Results section (p. 9, lines 1–6) in the revised manuscript. On the other hand, because CEP152-W105* and CEP152-K897* lack the C-terminal epitope recognized by this antibody, we tested a commercially available N-terminal CEP152 antibody (anti–CEP152-N; GeneTex Inc., # GTX128027). However, this antibody failed to detect CEP152 in Western blotting, making it difficult to detect endogenous CEP152-K897* by immunoblotting. Nevertheless, we found that this antibody successfully recognized endogenous CEP152, CEP152-Q32P, and CEP152-K897* in cortical slice (Fig. 2G in the revised manuscript) and MEFs (Fig. 2H in the revised manuscript), indicating that anti–CEP152-N recognizes conformational rather than linear epitopes. We described this issue in the revised manuscript (p. 9, lines 6–13).

We appreciate the reviewer's suggestion to validate CEP152 localization in a physiologically relevant context using MEFs. To this end, we prepared MEFs derived from the knock-in models and analyzed the subcellular localization of each CEP152 variant using the anti–CEP152-N. The results are presented in Fig. 2H, with corresponding revisions made in the Results section (p. 9, lines 6–13).

2. In Fig. 4, the authors propose that cell death primarily occurs in differentiated neurons rather than progenitor cells. To strengthen this conclusion, co-staining of Caspase-3 with specific

neuronal markers and quantification of neuron numbers across different cortical layers (as shown in Fig. 3D and F) are necessary. This would provide more robust evidence supporting the observed cell death phenotype.

Response: We appreciate the reviewer's insightful suggestion. In response, we performed double immunostaining on E15.5 cortical slices from Cep152$^{Q32P/Q32P}$ mice using anti-aCasp3 together with either anti-Tbr2 (a basal progenitor marker) or anti-βIII-tubulin (a neuronal marker). The results are presented in Fig. 4K of the revised manuscript. Corresponding descriptions have been added to the Results section (p. 12, lines 6 – 10) and the figure legend.

Regarding the quantification of neuron numbers across different cortical layers, we would like to note that apoptotic cells are predominantly observed around E15. Notably, at E15, the upper cortical layers have not yet formed, making it technically impossible to quantify neuron numbers according to cortical layers (as done for P0/P60 cortices). On the other hand, very few aCasp3–positive cells can be detected in the cortex by P0. For these reasons, layer-specific neuronal quantification cannot be performed at this developmental stage. We hope that these additional explanations help clarify the rationale behind our analyses and sufficiently address the reviewer's comment.

3. Representative images are required to accompany the quantification data presented in Fig. 4G and H. Furthermore, the number of Purkinje cells should be quantified from the images in Fig. 3K-N to provide a more comprehensive analysis of cerebellar abnormalities.

Response: In response to the reviewer's comment, we have included representative images (Fig. 4L and N in the revised manuscript) corresponding to the quantification data (previously Fig. 4G and H) shown. The figure legend has been updated accordingly. Additionally, we quantified the number of Purkinje cells using the images previously shown in Fig. 3K and M. The quantification results are now presented in Fig. 3O and R of the revised manuscript, with corresponding descriptions provided in the Results section (p. 10, lines 21 and 26–30) and in the figure legend.

4. Several centrosomal dysfunctions, such as defects in centrosome duplication or disengagement, could underlie the mitotic phenotypes observed in Fig. 4A-C. To further elucidate the impact of the two CEP152 variants on centrosome function, the following experiments using MEF cells from the knock-in models are recommended: 1) quantify the centriole number; 2) examine and quantify the centrosomal localization of PLK4 and other

duplication factors (such as CEP192); 3) quantify the percentage of abnormal mitotic cells.

Response:

We appreciate the reviewer's valuable suggestion and accordingly performed the recommended analyses using MEF cells derived from the knock-in models.

1) We quantified centriole numbers by staining with Centrin2, a marker for centrioles (Fig. 4D - G in the revised manuscript). We described the results in the revised manuscript (p. 11, lines 18 – 25).

2) We examined the localization of PLK4 and other centriole duplication factors. We first tested PLK4 using established anti-PLK4 antibodies (Proteintech, Cat#12952-1-AP; Santa Cruz, Cat#sc-100413); however, these antibodies did not recognize centrosomal PLK4. We next attempted to evaluate the localization of the CEP152-binding partner CPAP/CENPJ. However, the anti-CPAP antibody (Proteintech, Cat#11517-1-AP) failed to detect the target protein either in cortical slices from E15 brains or in MEFs, although the manufacturer's protocol indicates that this antibody is suitable for immunocytochemistry. Finally, we examined the distribution of CEP192, which is also known to interact with CEP152; however, the available anti-CEP192 (Proteintech, Cat No. 28700-1-AP) is raised against human CEP192, and the amino acid sequence of mouse CEP192 differs considerably from that of the human orthologue. We respectfully ask the reviewer to accept this limitation.

3) We then assessed the proportion of MEFs exhibiting abnormal mitotic figures (Fig. 4F and G in the revised manuscript). Descriptions of these experiments and the corresponding findings have been added to the Results section (p. 11, lines 22–25) of the revised manuscript.

Minor points:

1. The evolutionary conservation of the mutation sites between humans and mice should be presented to highlight their functional significance.

Response: We appreciate the reviewer's insightful comment. In response, we have included an analysis of the evolutionary conservation of the mutation sites between humans and mice in Fig. 1E of the revised manuscript to underscore their potential functional significance. The corresponding description has also been added to the Results section (p. 7, lines 10–12) and the figure legend.

2. The age of the mice used in each experiment should be clearly labeled in Fig. 3 and 4 to ensure reproducibility and contextual clarity.

Response: In response to the reviewer's comment, we have labeled the age of the mice used in each experiment in Fig. 3 and 4 in the revised manuscript.

3. The ventricular size shown in Fig. 3A should be quantified to provide a more objective assessment of the observed phenotype.

Response: In response to the reviewer's suggestion, we quantified the ventricular size shown in Fig. 3A and showed as Fig. 3B in the revised manuscript. The figure legend has been amended accordingly.

25th Feb 2026

Dear Dr. Nagata,

Thank you for the submission of your revised manuscript to EMBO Molecular Medicine. I am pleased to inform you that we will be able to accept your manuscript pending the following final amendments:

1) Please address referees' #1 and #2 minor concerns.
2) Authors: We note name discrepancy Shatha Alhifthi in the manuscript and Shatha Alhefdhi in our submission system. Please correct.
3) Figures:
- Figure EV1B - Please add a black box to cover the eyes of the patient.
- The resolution of the submitted figures is too low for blot and microscopy images. This reduction in resolution is commonly caused by converting original 16-bit TIFF files to RGB format for publication. While this is not inherently problematic, it can raise concerns about image integrity for critical readers. To avoid any misunderstanding and to meet EMBO Press standards, we kindly ask that you:
1. Resubmit the complete figure set at the captured original data resolution.
2. Apply the same resolution standards to the source data files.
4) In the main manuscript file, please do the following:
- Please address all comments suggested by our data editors listed below:
1. Please note that the box plots need to be defined in terms of minima, maxima, center, bounds of box and whiskers, and percentile in the legends of figures 3I, EV3 A, B, EV4 I, J.
2. Please note that information related to n is missing in the legends of figures 5F, G; 9A, B
3. Please note that the error bars are not defined in the legends of figures 2D-F, 3B, D, F, G, K, L, N, O, Q, R; 4B, C, E, G, J, M, O; 5F, G; 8D, E; EV2 E, F.
- Please correct the order and headings of the sections to: Abstract / Keywords / The Paper Explained / Introduction / Results / Discussion / Methods / Data Availability / Acknowledgements / Disclosure and Competing Interests Statement / References / Main Figure Legends / Expanded View Figure Legends.
- Please remove "data not shown" (3 mentions on p.8).
- Remove the abbreviation list. The abbreviations should be incorporated into the text.
- In Methods, please confirm that in addition to the WMA Declaration of Helsinki experiments also conformed to the principles set out in the Department of Health and Human Services Belmont Report.
- In the "Disclosure and competing interests statement" please state employment in a company (affiliation #16).
- Author contributions: Please remove it from the manuscript and specify author contributions in our submission system. CRediT has replaced the traditional author contributions section because it offers a systematic machine-readable author contributions format that allows for more effective research assessment. You are encouraged to use the free text boxes beneath each contributing author's name to add specific details on the author's contribution. More information is available in our guide to authors:
https://www.embopress.org/page/journal/17574684/authorguide#authorshipguidelines
- Remove the EV table legends from the manuscript text and add them to the corresponding files.
- In data availability statement remove the current sentence and provide information about raw data from large-scale datasets like RNA-seq that should be deposited in one of the relevant databases and made freely available prior the publication of the manuscript. Use the following format to report the accession number of your data:

The datasets produced in this study are available in the following databases:
[data type]: [full name of the resource] [accession number/identifier] ([doi or URL or identifiers.org/DATABASE:ACCESSION])

Please check "Author Guidelines" for more information.
https://www.embopress.org/page/journal/17574684/authorguide#availabilityofpublishedmaterial
5) Tables: Please correct the nomenclature in the legend of Table EV1 and add a legend to the top of the page for Table EV2. Table EV3 and EV4 should be renamed Datatset EV1 and Dataset EV2, and both files will need legends added, either in a separate tab/worksheet, or as a simple readme text file zipped to each csv file.
6) Funding: Please merge it with "Acknowledgments" and make sure that information about all sources of funding are complete in both our submission system and in the manuscript. Currently the following funding sources are missing in our submission system: Grant-in-Aid for Transformative Research Areas — Platforms for Advanced Technologies and Research Resources "Advanced Bioimaging Support".
7) The Paper Explained: Please provide "The Paper Explained" and add it to the main manuscript text. Please check "Author Guidelines" for more information. https://www.embopress.org/page/journal/17574684/authorguide#researcharticleguide
8) Synopsis:
- Synopsis image: Please upload the image to 550 px-wide x 300-600 pixels high and upload it as a high-resolution jpeg file.
- Please check your synopsis text and image before submission with your revised manuscript. Please be aware that in the proof stage minor corrections only are allowed (e.g., typos).

9) As part of the EMBO Publications transparent editorial process (see our Editorial at http://embomolmed.embopress.org/content/2/9/329), EMBO Molecular Medicine will publish online a Review Process File (RPF) to accompany accepted manuscripts. This file will be published in conjunction with your paper and will include the anonymous referee reports, your point-by-point response and all pertinent correspondence relating to the manuscript. Let us know if you want to remove or not any figures from it prior to publication. Please note that the Authors checklist will be published at the end of the RPF.
10) Please provide a point-by-point letter INCLUDING my comments as well as the reviewer's reports and your detailed responses (as Word file).

I look forward to reading a new revised version of your manuscript as soon as possible.

Yours sincerely,

Zeljko Durdevic

Zeljko Durdevic
Senior Editor
EMBO Molecular Medicine

*** Instructions to submit your revised manuscript ***

*** PLEASE NOTE *** As part of the EMBO Publications transparent editorial process initiative (see our Editorial at https://www.embopress.org/doi/pdf/10.1002/emmm.201000094), EMBO Molecular Medicine will publish online a Review Process File to accompany accepted manuscripts.

In the event of acceptance, this file will be published in conjunction with your paper and will include the anonymous referee reports, your point-by-point response and all pertinent correspondence relating to the manuscript. If you do NOT want this file to be published, please inform the editorial office at contact@embomolmed.org.

*****

When preparing your revised manuscript, please refer to our guidelines: https://link.springer.com/journal/44321/submission-guidelines#cms-Revised-submissions. We perform an initial quality control of all revised manuscripts before re-review; failure to include requested items will delay the evaluation of your revision.

We require:

1) A .docx formatted version of the manuscript text (including legends for main figures, EV figures and tables). Please make sure that the changes are highlighted to be clearly visible.

2) Individual production quality figure files as .eps, .tif, .jpg (one file per figure). For guidance, download the 'Figure Guide PDF': https://media.springernature.com/original/springer-cms/rest/v1/content/27825798/data/v1.

3) A .docx formatted letter INCLUDING the reviewers' reports and your detailed point-by-point responses to their comments. As part of the EMBO Press transparent editorial process, the point-by-point response is part of the Review Process File (RPF), which will be published alongside your paper.

4) A complete author checklist, which you can download from our author guidelines. Please insert information in the checklist that is also reflected in the manuscript. The completed author checklist will also be part of the RPF.

5) Please note that all corresponding authors are required to supply an ORCID ID for their name upon submission of a revised manuscript.

6) It is mandatory to include a 'Data Availability' section after the Materials and Methods. Before submitting your revision, primary

datasets produced in this study need to be deposited in an appropriate public database, and the accession numbers and database listed under 'Data Availability'. Please remember to provide a reviewer password if the datasets are not yet public.

In case you have no data that requires deposition in a public database, please state so in this section. Note that the Data Availability Section is restricted to new primary data that are part of this study.

7) For data quantification: please specify the name of the statistical test used to generate error bars and P values, the number (n) of independent experiments (specify technical or biological replicates) underlying each data point and the test used to calculate p-values in each figure legend. The figure legends should contain a basic description of n, P and the test applied. Graphs must include a description of the bars and the error bars (s.d., s.e.m.).

8) At EMBO Press we ask authors to provide source data for the main manuscript figures. You will receive a separate email with instructions for providing source data with your revised manuscript, including how to upload and organize the files.

9) Our journal encourages inclusion of *data citations in the reference list* to directly cite datasets that were re-used and obtained from public databases. Data citations in the article text are distinct from normal bibliographical citations and should directly link to the database records from which the data can be accessed. In the main text, data citations are formatted as follows: "Data ref: Smith et al, 2001" or "Data ref: NCBI Sequence Read Archive PRJNA342805, 2017". In the Reference list, data citations must be labeled with "[DATASET]". A data reference must provide the database name, accession number/identifiers and a resolvable link to the landing page from which the data can be accessed at the end of the reference.

10) We replaced Supplementary Information with Expanded View (EV) Figures and Tables that are collapsible/expandable online. A maximum of 5 EV Figures can be typeset. EV Figures should be cited as 'Figure EV1, Figure EV2" etc... in the text and their respective legends should be included in the main text after the legends of regular figures.

- For the figures that you do NOT wish to display as Expanded View figures, they should be bundled together with their legends in a single PDF file called *Appendix*, which should start with a short Table of Content. Appendix figures should be referred to in the main text as: "Appendix Figure S1, Appendix Figure S2" etc.

- Additional Tables/Datasets should be labeled and referred to as Table EV1, Dataset EV1, etc. Legends have to be provided in a separate tab in case of .xls files. Alternatively, the legend can be supplied as a separate text file (README) and zipped together with the Table/Dataset file.

11) The paper explained: EMBO Molecular Medicine articles are accompanied by a summary of the articles to emphasize the major findings in the paper and their medical implications for the non-specialist reader. Please provide a draft summary of your article highlighting

- the medical issue you are addressing,

- the results obtained and

- their clinical impact.

This may be edited to ensure that readers understand the significance and context of the research. Please refer to any of our published articles for an example.

12) Author contributions: You will be asked to provide CRediT (Contributor Role Taxonomy) terms in the submission system. These replace a narrative author contribution section in the manuscript.

13) A Conflict of Interest statement should be provided in the main text.

14) Every published paper includes a 'Synopsis' to further enhance discoverability. Synopses are displayed on the journal webpage and are freely accessible to all readers. They include a short stand first (maximum of 300 characters, including space) as well as 2-5 one-sentences bullet points that summarizes the paper. Please write the bullet points to summarize the key NEW findings. They should be designed to be complementary to the abstract - i.e. not repeat the same text. We encourage inclusion of key acronyms and quantitative information (maximum of 30 words / bullet point). Please use the passive voice. Please attach these in a separate file or send them by email, we will incorporate them accordingly.

Please also suggest a striking image or visual abstract to illustrate your article as a PNG file 550 px wide x 300-600 px high.

15) Include a Reagents and Tools Table as part of the Methods section, which can be downloaded from our author guidelines.

EMBO Molecular Medicine has a "scooping protection" policy, whereby similar findings that are published by others during

review or revision are not a criterion for rejection. Should you decide to submit a revised version, I do ask that you get in touch after three months if you have not completed it, to update us on the status.

*Additional important information regarding Figures

Each figure should be given in a separate file and should have the following resolution:
Graphs 800-1,200 DPI
Photos 400-800 DPI
Colour (only CMYK) 300-400 DPI"

Figures are not edited by the production team. All lettering should be the same size and style; figure panels should be indicated by capital letters (A, B, C etc). Gridlines are not allowed except for log plots. Figures should be numbered in the order of their appearance in the text with Arabic numerals. Each Figure must have a separate legend and a caption is needed for each panel.

*Additional important information regarding figures and illustrations can be found at
https://media.springernature.com/original/springer-cms/rest/v1/content/27825798/data/v1

***** Reviewer's comments *****

Referee #1 (Remarks for Author):

I acknowledge that the authors made a lot of efforts to reply to all my concerns and/or comments. I am convinced that the results presented are almost ready for publication. Nonetheless, I would recommend some clarifications of the figures, especially of the new panels provided to help the readers understand what they are looking at.
1) In Figure 2G, a low magnification image should be provided (similar to the ones in Figures EV4D-E and EV4F-G) so that we understand that these panels are higher magnifications of the ventricular lining, where centrosomes are located. Can the authors also provide a co-staining with a membrane marker (like phalloidin that stains actin at the plasma membrane for example), so that the sub-cellular localization in the cytoplasm is clearer for the W105/K897 mutant?
2) The same is valid for Figure EV4A, a lower magnification and a membrane marker co-staining should be provided. In this case, can the authors suggest in the main result section, that the reduction in the number of cilia is likely due to a decrease in the population of Pax6 positive apical progenitors, as stated in the rebuttal letter?
3) The same comment for figure EV3D-E, a lower magnification and a membrane marker co-staining should be provided.
4) The authors should provide the same evaluation of the reduction in the number of Pax6 positive cells, as for Tbr2 positive cells (Figure EV3F), instead of VZ thickness only (Figure 3I).
5) In figure EV3B-C, can the authors highlight on the panels the observed defects between control and mutant sections?
6) It would make much more sense for the flow of the manuscript that Figure 8 (characterization of interactions between Cep152 and Plk4) appears as Figure 6, before the characterization of neuron morphology and activity, current figures 6 /7.
7) I also have some questions after the new EM data provided in Figure 5. It now clearly appears that mitosis in mutant brains display abnormalities in the number of centrioles per cell (Figure 4F-G) and also structural abnormalities in the case of the Q32P mutant (Figure 5). Nonetheless, if the authors evaluate between 20 to 30% the number of MEF mitosis with 0-3 centrioles per cell, the same evaluation performed after EM in the mitotic apical progenitor population report less than 5% of centriole number abnormalities in Figure 5G for the W105/K897 mutant. Can the authors add a comment regarding this point in the main result section of the manuscript?
8) In Figure 5, the contrast should be increased so that the centriole structures are more visible in the mitotic cells.
9) Phospho-histone 3 is normally abbreviated as PH3, and not pHH3.
10) The authors have now enough convincing evidence that their Cep152 mutant brains display a centriole duplication defect (preponderance of monopolar spindles in mitosis and mitosis with 1,2 or 3 centrioles instead of 4). They should state it more clearly in the main text.

Referee #2 (Remarks for Author):

The authors have added new clinical and experimental data that greatly improve the quality of the article. In particular, they clarified the phenotype of patients 1 and 2 which they analyzed in parallel with the brain phenotype of the mutant mice and they analyzed the consequences of variants on centriole biogenesis and centrosome structure in mutant mice.
However, I recommend that the few following points be explored in greater depth.

Patient's phenotype:
The authors succeeded in demonstrating the origin of the blindness for patient 2 and in characterizing the cerebral anatomy of

this child.
1. The axial Flair images (Fig EV1E) is convincing, in showing the characteristic flame-like appearance of retinal detachment. However, the persistence of the hyperplastic primary vitreous is not visible on this sequence. I suggest to mention it using the conditional tense.
2. Sagital and axial sections show very severe microcephaly and gyral simplification, I agree with the authors.

For Patient 3:
The structure of the brain is still difficult to characterize. I agree that the position of the child and the presence of the interhemispheric cyst do not allow for strict sagittal sections to be made.
The corpus callosum is present anteriorly (sagittal sections, Figure 1C, and coronal section EV1F). This could be hypoplasia (prefer this term to that of hypogenesis p 6 l33) or dysgenesis, as for patient 1.
The gyration of the cortex is probably not normal in contact with the cyst on the right side of the EV1 G image. Coronal image F is more convincing and clearly shows polymicrogyria of the hemisphere on the right side of the image.
Rather than image EV1G, I would suggest that the reviewers show several sequential coronal sections showing the cortex and passing through the corpus callosum before and after the EV1F coronal image to clarify the phenotype (extensive polymicrogyria? Unilateral or on both hemispheres? Hypoplasia or dysgenesis of the corpus callosum?).
To assist the reader, I would suggest that the authors group all MRI and CT images of patient 3 in Figure 1, even if it means reducing the size of Figures 1A and B.

Given that the phenotypes of the two patients carrying the same homozygous c.95A>C variant are ultimately different (polymicrogyria in patient 3 but not in patient 2), could the authors verify that the genes involved in autosomal recessive polymicrogyria have been carefully examined in patient 3 and do not contain deleterious variants?

P7 l4: "The medical chart of the patient stated that the blindness was likely due to persistent hyperplastic primary vitreous". Instead of this sentence, I suggest that authors write in the manuscript what they wrote in their point-by-point response, using the conditional tense.

in vitro experiments or in vivo models
1. Morphology/structure of the cerebral cortex:
Figures 2B, C, and F clearly show the decrease in volume of the telencephalic vesicles in mutants compared to controls. Figures 3E-G and EV3 show the reduction of the cortical thickness. In Figure EV3C, does the red labeling correspond to Cux1? Please indicate this. Cux1 staining should be nuclear. This appears to be the case in the right panel (P10), but not in the left panel (P0). Do the authors have other images to provide? Please show the images at the same scale between P0 and P10. Could the authors place the third panel (QQ32P/Q32P) of Figure 3C next to the first two panels and graphs 3F-G?

2. Morphology /structure of the cerebellar cortex:
The authors have now shown that there are more Purkinje cells in the cerebellum of Q32P/ Q32P mutants than in controls at P6, and that a fraction of these Purkinje cells are mis-localized (in the IGL rather than the PCL, Figures 3 P-R), at a stage when control Purkinje cells have completed their migration and are arranged in a monolayer.
As at P60, the Purkinje cells of the mutants:
- are reduced as compared to controls
- remained mis-localized in the IGL,
It would be interesting to discuss (not in the results section but in the discussion section):
- the hypothesis of premature differentiation and excessive apoptosis of Purkinje cells
- that this would not indicate a delay but rather a migration disorder of Purkinje cells.

3. Apoptosis
The authors now clarified the identity of the apoptotic cells (neurons > Tbr2 progenitors)

4. Biogenesis of the centriole and Structure of the centrosome
The authors showed magnificent electron microscopy images of the structure of centrioles in the mutant brains and MEFs (Figure 5), and immunofluorescence experiments (figures 4 and EV3) which perfectly answered the questions that had been raised.

Specific comments:
P6 l10: change "microcephalus" for "microcephaly"
P7 l30: "which possess an intact centrosome structure": please indicate the corresponding figure
P8 l13-14: The PCM structure appeared not to be affected by the expression of the three CEP152 variants (Fig. 2C, b'- d'). Is the reference number for the figure correct? The figures 2C b'-d' do not show the PCM.
P18 l7: "These abnormalities included abnormal cortical lamination". Since the localization of ctip2- and cux1-positive neurons is similar in controls and mutants, the following sentence would seem more appropriate: The thickness of the deep and superficial layers is reduced in mutants compared to controls.
P18 l10 "These results suggest cytoskeletal dysregulation", p18 l12 "Collectively, these findings indicate that the p.Q32P variant impairs early neuronal development through cytoskeletal signaling disruption" and p19 l1 "These findings suggest that the

p.Q32P variant primarily disrupts synaptic function through cytoskeleton-based structural impairments". Without any new experiments to support abnormal cytoskeleton in Q32P/Q32P neurons, I recommend not speculating on this hypothesis.
P27 l11: please change Hypogenesis to hypoplasia

EV1 Table has been now completed. Please review the layout of the EV1 table and references.

Referee #3 (Comments on Novelty/Model System for Author):

In this work, the authors appiled knock-in mouse model and MEF cell culture to mimic the phenotypes in CEP152 muntant patients. Overall, the cell biology and mouse model experiments are well designed, and the results are solid.

Referee #3 (Remarks for Author):

In this round of revision work, the authors have addressed all my concerns on the function of CEP152 mutants on centrosomes, I recommended to publish this manuscript as the current version.

Referee #1 (Remarks for Author):

I acknowledge that the authors made a lot of efforts to reply to all my concerns and/or comments. I am convinced that the results presented are almost ready for publication. Nonetheless, I would recommend some clarifications of the figures, especially of the new panels provided to help the readers understand what they are looking at.

1) In Figure 2G, a low magnification image should be provided (similar to the ones in Figures EV4D-E and EV4F-G) so that we understand that these panels are higher magnifications of the ventricular lining, where centrosomes are located. Can the authors also provide a co-staining with a membrane marker (like phalloidin that stains actin at the plasma membrane for example), so that the sub-cellular localization in the cytoplasm is clearer for the W105/K897 mutant?

We thank the reviewer for this helpful suggestion. We carefully examined whether a low-magnification image could be provided for Fig. 2G. However, because centrosomes are extremely small intracellular organelles, they cannot be clearly identified at low magnification

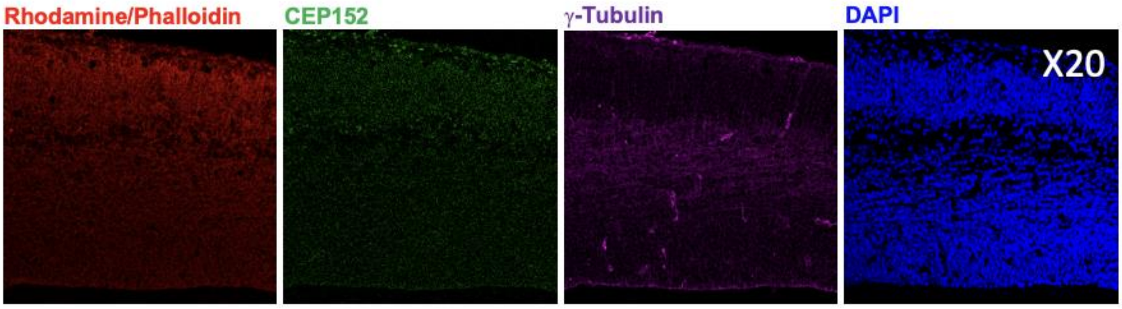

in ventricular zone images. In such images, the centrosomal signals are not distinguishable from the surrounding cellular structures, making it difficult to interpret the localization of CEP152 (please see Figures below). For this reason, we present higher-magnification images that allow clear visualization of centrosomes, which we believe provide a more accurate representation of the subcellular localization. Representative images have been carefully selected to show the centrosomal localization of CEP152 in ventricular lining cells.

In addition, we attempted co-staining of the W105/K897 mutant with anti-CEP152 and rhodamine-phalloidin to visualize the plasma membrane. However, rhodamine-phalloidin did not provide reliable membrane labeling under our experimental conditions as shown below. As shown in Fig EV4F, the monoclonal anti-Nestin antibody stains the cell membrane. However, the anti-γ-tubulin and anti-Arl13b antibodies are also monoclonal antibodies, which makes it difficult to perform multiple staining. Therefore, we present images double-stained with anti-CEP152 and DAPI (Fig. 2G in the revised manuscript), which clearly demonstrate the cytoplasmic distribution of CEP152 in the mutant. The corresponding figure legend has been revised accordingly.

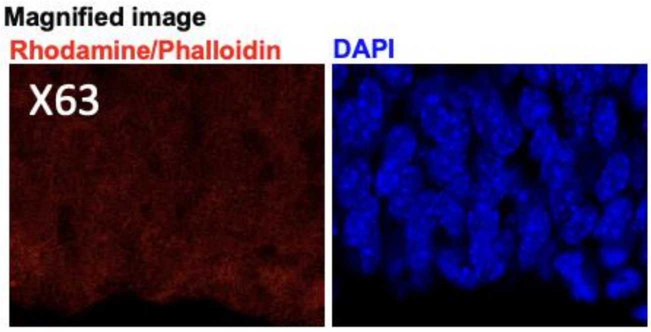

2) The same is valid for Figure EV4A, a lower magnification and a membrane marker co-staining should be provided. In this case, can the authors suggest in the main result section, that the reduction in the number of cilia is likely due to a decrease in the population of Pax6 positive apical progenitors, as stated in the rebuttal letter?

As in the case of the comment #1, primary cilia cannot be clearly identified at low magnification in ventricular zone images. In such images, the Arl13b (a cilia marker) signals are not distinguishable from the surrounding cellular structures. For this reason, we present higher-magnification images that allow clear visualization of primary cilia. In addition, as in the case of Fig. 2G, co-staining with anti-CEP152 and rhodamine-phalloidin did not provide reliable membrane labeling.

Following the reviewer's suggestion, we have added a statement in the Results section indicating that the reduction in the number of cilia is likely due to a decrease in the population of Pax6-positive apical progenitors in the revised manuscript (p. 12, lines 32– p. 13, lines 1).

3) The same comment for figure EV3D-E, a lower magnification and a membrane marker co-staining should be provided.

We apologize if this was not sufficiently clear in the original version. Figures EV3D and EV3E already show low-magnification images of the ventricular lining. We hope this clarifies the concern; however, we would be happy to provide additional images if needed.

4) The authors should provide the same evaluation of the reduction in the number of Pax6 positive cells, as for Tbr2 positive cells (Figure EV3F), instead of VZ thickness only (Figure 3I).

We appreciate the reviewer's comment and re-made Figure 3I to show the evaluation of the reduction in the number of Pax6 positive cells in the revised manuscript. Accordingly, the figure legend has been amended.

5) In figure EV3B-C, can the authors highlight on the panels the observed defects between control and mutant sections?

Following the reviewer's suggestion, we have added control images to Fig. EV3C and indicated the observed defects by arrowheads to facilitate clearer comparison between control and the Cep152$^{Q32P/Q32P}$ sections.

6) It would make much more sense for the flow of the manuscript that Figure 8 (characterization of interactions between Cep152 and Plk4) appears as Figure 6, before the characterization of neuron morphology and activity, current figures 6 /7.

We appreciate the reviewer's insightful comment. We have moved the subsection "CEP152 is crucial for PLK4 localization at the centrosome" so that it now appears before the sections describing neuronal morphology and activity (formerly Figs. 6 and 7).

7) I also have some questions after the new EM data provided in Figure 5. It now clearly appears that mitosis in mutant brains display abnormalities in the number of centrioles per cell (Figure 4F-G) and also structural abnormalities in the case of the Q32P mutant (Figure 5). Nonetheless, if the authors evaluate between 20 to 30% the number of MEF mitosis with 0-3 centrioles per cell, the same evaluation performed after EM in the mitotic apical progenitor population report less than 5% of centriole number abnormalities in Figure 5G for the W105/K897 mutant. Can the authors add a comment regarding this point in the main result section of the manuscript?

We thank the reviewer for this thoughtful comment. The observed difference may be attributed to cell type–specific effects and methodological differences between MEF immunostaining and tissue EM analyses of apical progenitors. We have now added a statement in the Results section to clarify this point and discuss the potential reasons for this discrepancy (p. 13, lines 20–24).

8) In Figure 5, the contrast should be increased so that the centriole structures are more visible in the mitotic cells.

We thank the reviewer for this comment. The contrast of the images in Figure 5 has been increased to improve the visibility of centriole structures in mitotic cells.

9) Phospho-histone 3 is normally abbreviated as PH3, and not pHH3.

We thank the reviewer for the comment. We have now changed pHH3 to PH3 throughout the manuscript.

10) The authors have now enough convincing evidence that their Cep152 mutant brains display a centriole duplication defect (preponderance of monopolar spindles in mitosis and mitosis with 1,2 or 3 centrioles instead of 4). They should state it more clearly in the main text.

We appreciate the reviewer's comment. We have now stated that the Cep152 mutant brains

display a centriole duplication defect (preponderance of monopolar spindles in mitosis and mitosis with 1,2 or 3 centrioles instead of 4) (p. 11, lines 30– p. 12, lines 3).

Referee #2 (Remarks for Author):

The authors have added new clinical and experimental data that greatly improve the quality of the article. In particular, they clarified the phenotype of patients 1 and 2 which they analyzed in parallel with the brain phenotype of the mutant mice and they analyzed the consequences of variants on centriole biogenesis and centrosome structure in mutant mice.

However, I recommend that the few following points be explored in greater depth.

Patient's phenotype:

The authors succeeded in demonstrating the origin of the blindness for patient 2 and in characterizing the cerebral anatomy of this child.

1. The axial Flair images (Fig EV1E) is convincing, in showing the characteristic flame-like appearance of retinal detachment. However, the persistence of the hyperplastic primary vitreous is not visible on this sequence. I suggest to mention it using the conditional tense.

We thank the reviewer for this insightful comment. We agree that persistent hyperplastic primary vitreous cannot be directly visualized on the axial FLAIR sequence. Therefore, we have revised the corresponding description in the manuscript to reflect this interpretation in the conditional tense (p. 6, lines 13–15).

2. Sagital and axial sections show very severe microcephaly and gyral simplification, I agree with the authors.

We appreciate the reviewer's comment.

For Patient 3:

The structure of the brain is still difficult to characterize. I agree that the position of the child and the presence of the interhemispheric cyst do not allow for strict sagittal sections to be made.

The corpus callosum is present anteriorly (sagittal sections, Figure 1C, and coronal section EV1F). This could be hypoplasia (prefer this term to that of hypogenesis p 6 l33) or dysgenesis, as for patient 1.

We appreciate the reviewer’s comment and have changed the term “hypogenesis” to “hypoplasia” in the revised manuscript (p. 6, line 31).

The gyration of the cortex is probably not normal in contact with the cyst on the right side of the EV1 G image. Coronal image F is more convincing and clearly shows polymicrogyria of the hemisphere on the right side of the image.
Rather than image EV1G, I would suggest that the reviewers show several sequential coronal sections showing the cortex and passing through the corpus callosum before and after the EV1F coronal image to clarify the phenotype (extensive polymicrogyria? Unilateral or on both hemispheres? Hypoplasia or dysgenesis of the corpus callosum?).

We thank the reviewer for this helpful suggestion. To better clarify the phenotype, we have added four additional sequential coronal sections (Fig. 1E) in the revised manuscript, showing the cortex and corpus callosum around the region previously presented in Fig. EV1F. These images clearly demonstrate polymicrogyria and hypoplasia of the corpus callosum. The corresponding description has been added to the revised manuscript (p. 6, line 32 – p. 7, line 2).

To assist the reader, I would suggest that the authors group all MRI and CT images of patient 3 in Figure 1, even if it means reducing the size of Figures 1A and B.
We appreciate the reviewer’s insightful suggestion. In the revised manuscript, all MRI and CT images of Patient 3 have been grouped in Figure 1. The corresponding text has been revised accordingly (p. 6, line 32 - p. 7, line 2, and the figure legend).

Given that the phenotypes of the two patients carrying the same homozygous c.95A>C variant are ultimately different (polymicrogyria in patient 3 but not in patient 2), could the authors verify that the genes involved in autosomal recessive polymicrogyria have been carefully examined in patient 3 and do not contain deleterious variants?
We thank the reviewer for this important question. We carefully re-examined the whole-exome sequencing data of Patient #3, focusing specifically on genes known to be associated with autosomal recessive polymicrogyria. No pathogenic or likely pathogenic variants were identified in these genes. In addition, no rare deleterious variants with predicted functional impact were detected that could plausibly explain the polymicrogyria phenotype independently of the CEP152 variant.

These findings support the conclusion that the observed cortical malformation is most likely attributable to the homozygous p.Q32P variant in CEP152. However, we cannot completely exclude the possibility of unidentified genetic modifiers contributing to the phenotypic variability between the two patients. We have clarified this point in the revised manuscript (p. 17, lines 17-22).

P7 l4: "The medical chart of the patient stated that the blindness was likely due to persistent hyperplastic primary vitreous". Instead of this sentence, I suggest that authors write in the manuscript what they wrote in their point-by-point response, using the conditional tense.
We appreciate the reviewer's helpful comment. The corresponding sentence has been revised in the manuscript to use the conditional tense (p. 7, lines 4–6).

in vitro experiments or in vivo models
1. Morphology/structure of the cerebral cortex:
Figures 2B, C, and F clearly show the decrease in volume of the telencephalic vesicles in mutants compared to controls.
We appreciate the reviewer's comment.

Figures 3E-G and EV3 show the reduction of the cortical thickness. In Figure EV3C, does the red labeling correspond to Cux1? Please indicate this.
In line with the reviewer's suggestion, we indicated that red and green labeling show Cux1 and Ctip2, respectively, in Fig. EV3C in the revised manuscript.

Cux1 staining should be nuclear. This appears to be the case in the right panel (P10), but not in the left panel (P0). Do the authors have other images to provide? Please show the images at the same scale between P0 and P10. Could the authors place the third panel (Q32P/Q32P) of Figure 3C next to the first two panels and graphs 3F-G?
We thank the reviewer for this helpful comment. The Cux1 antibody from GeneTex (Cat# GTX56275) also detects a Golgi-localized Cux1 isoform. During early postnatal stages, Cux1 is predominantly expressed in a Golgi-associated form, whereas with maturation the nuclear form becomes more prominent. Although the Golgi-associated and nuclear forms share the same N-terminal sequence, the GeneTex antibody was raised against an N-terminal epitope. Therefore, this antibody detects the Golgi-associated Cux1 isoform present during early developmental

stages. This property of Cux1 localization has been reported previously (Alison K. et al., *Molecular Biology of the Cell* 13, 3761–3774, 2002). This point has been clarified in the figure legend of Figure EV3C in the revised manuscript. We now show the P0 and P10 images at the same scale. In addition, we have placed the third panel (Q32P/Q32P) of Fig. 3E next to the first two panels. Although we attempted to reposition graphs 3F and G for improved clarity, only graph 3F could be moved due to space constraints.

2. Morphology /structure of the cerebellar cortex:
The authors have now shown that there are more Purkinje cells in the cerebellum of Q32P/Q32P mutants than in controls at P6, and that a fraction of these Purkinje cells are mis-localized (in the IGL rather than the PCL, Figures 3 P-R), at a stage when control Purkinje cells have completed their migration and are arranged in a monolayer.
As at P60, the Purkinje cells of the mutants:
- are reduced as compared to controls
- remained mis-localized in the IGL,
It would be interesting to discuss (not in the results section but in the discussion section):
- the hypothesis of premature differentiation and excessive apoptosis of Purkinje cells
- that this would not indicate a delay but rather a migration disorder of Purkinje cells.
We appreciate the reviewer’s insightful suggestion. We discussed the interesting phenotypes pointed out by the reviewer (p. 18, lines 12– 16 in the revised manuscript).

3. Apoptosis
The authors now clarified the identity of the apoptotic cells (neurons > Tbr2 progenitors)
We thank the reviewer for this comment.

4. Biogenesis of the centriole and Structure of the centrosome
The authors showed magnificent electron microscopy images of the structure of centrioles in the mutant brains and MEFs (Figure 5), and immunofluorescence experiments (figures 4 and EV3) which perfectly answered the questions that had been raised.
We thank the reviewer for this comment.

Specific comments:
P6 l10: change "microcephalus" for "microcephaly"

According to the reviewer’s suggestion, we changed "microcephalus" to "microcephaly" in the revised manuscript (p. 6, line 8).

P7 l30: "which possess an intact centrosome structure": please indicate the corresponding figure

We thank the reviewer for this comment. We have added a reference to Fig. 2B in the revised manuscript to indicate the corresponding figure (p. 7, line 31).

P8 l13-14: The PCM structure appeared not to be affected by the expression of the three CEP152 variants (Fig. 2C, b'- d'). Is the reference number for the figure correct? The figures 2C b'-d' do not show the PCM.

We apologize for the confusion. In this experiment, γ-tubulin was used as a marker of the PCM; however, we agree that the original wording may have been misleading. We have therefore revised the sentence to clarify this point (p. 8, lines 14– 15 in the revised manuscript).

P18 l7: "These abnormalities included abnormal cortical lamination". Since the localization of ctip2- and cux1-positive neurons is similar in controls and mutants, the following sentence would seem more appropriate: The thickness of the deep and superficial layers is reduced in mutants compared to controls.

We appreciate the reviewer’s helpful suggestion. We have revised the sentence accordingly to state that the thickness of the deep and superficial layers is reduced in mutants compared to controls (p. 18, lines 11– 12 in the revised manuscript).

P18 l10 "These results suggest cytoskeletal dysregulation", p18 l12 "Collectively, these findings indicate that the p.Q32P variant impairs early neuronal development through cytoskeletal signaling disruption" and p19 l1 "These findings suggest that the p.Q32P variant primarily disrupts synaptic function through cytoskeleton-based structural impairments". Without any new experiments to support abnormal cytoskeleton in Q32P/Q32P neurons, I recommend not speculating on this hypothesis.

We appreciate the reviewer’s insightful comment. We agree that the original statements were speculative in the absence of direct experimental evidence for cytoskeletal abnormalities. Accordingly, we have removed the phrases “These results suggest cytoskeletal dysregulation,”

"through cytoskeletal signaling disruption," and "through cytoskeleton-based structural impairments" from the revised manuscript.

P27 l11: please change Hypogenesis to hypoplasia

We changed the term "hypogenesis" to "hypoplasia" throughout the text.

EV1 Table has been now completed. Please review the layout of the EV1 table and references.

We have reviewed the layout and references of Table EV1. In addition, for improved readability and convenience for readers, the table has been provided as a PDF file.

30th Mar 2026

Dear Dr. Nagata,

We are pleased to inform you that your manuscript is accepted for publication and is now being sent to our publisher to be included in the next available issue of EMBO Molecular Medicine.

Your manuscript will be processed for publication by EMBO Press. It will be copy edited and you will receive page proofs prior to publication. Please note that you will be contacted by Springer Nature Author Services to complete licensing and payment information.

You may qualify for financial assistance for your publication charges - either via a Springer Nature fully open access agreement or an EMBO initiative. Check your eligibility: https://link.springer.com/journal/44321/how-to-publish-with-us

Should you be planning a Press Release on your article, please get in contact with embo_production@springernature.com as early as possible in order to coordinate publication and release dates.

If you have any questions, please do not hesitate to contact the Editorial Office. Thank you for your contribution to EMBO Molecular Medicine.

Yours sincerely,
Zeljko Durdevic

Zeljko Durdevic
Senior Editor
EMBO Molecular Medicine

>>> Please note that it is EMBO Molecular Medicine policy for the transcript of the editorial process (containing referee reports and your response letter) to be published as an online supplement to each paper. If you do NOT want this, you will need to inform the Editorial Office via email immediately. More information is available here: https://link.springer.com/partners/embo-press/editorial-policies#Peer%20review

EMBO Press Author Checklist

Corresponding Author Name: Koh-ichi Nagata	
Journal Submitted to: EMBO Molecular Medicine	
Manuscript Number: EMM-2025-21938-V2	

USEFUL LINKS FOR COMPLETING THIS FORM
The EMBO Journal - Author Guidelines
EMBO Reports - Author Guidelines
Molecular Systems Biology - Author Guidelines
EMBO Molecular Medicine - Author Guidelines

Reporting Checklist for Life Science Articles (updated January
This checklist is adapted from Materials Design Analysis Reporting (MDAR) Checklist for Authors. MDAR establishes a minimum set of requirements in transparent reporting in the life sciences (see Statement of Task: 10.31222/osf.io/9sm4x). Please follow the journal's guidelines in preparing your
Please note that a copy of this checklist will be published alongside your article.

Abridged guidelines for figures
1. Data
The data shown in figures should satisfy the following conditions:
- the data were obtained and processed according to the field's best practice and are presented to reflect the results of the experiments in an accurate and unbiased manner.
- ideally, figure panels should include only measurements that are directly comparable to each other and obtained with the same assay.
- plots include clearly labeled error bars for independent experiments and sample sizes. Unless justified, error bars should not be shown for technical
- if n<5, the individual data points from each experiment should be plotted. Any statistical test employed should be justified.
- Source Data should be included to report the data underlying figures according to the guidelines set out in the authorship guidelines on Data

2. Captions
Each figure caption should contain the following information, for each panel where they are relevant:
- a specification of the experimental system investigated (eg cell line, species name).
- the assay(s) and method(s) used to carry out the reported observations and measurements.
- an explicit mention of the biological and chemical entity(ies) that are being measured.
- an explicit mention of the biological and chemical entity(ies) that are altered/varied/perturbed in a controlled manner.
- the exact sample size (n) for each experimental group/condition, given as a number, not a range;
- a description of the sample collection allowing the reader to understand whether the samples represent technical or biological replicates (including how many animals, litters, cultures, etc.).
- a statement of how many times the experiment shown was independently replicated in the laboratory.
- definitions of statistical methods and measures:
  - common tests, such as t-test (please specify whether paired vs. unpaired), simple χ^2 tests, Wilcoxon and Mann-Whitney tests, can be unambiguously identified by name only, but more complex techniques should be described in the methods section;
  - are tests one-sided or two-sided?
  - are there adjustments for multiple comparisons?
  - exact statistical test results, e.g., P values = x but not P values < x;
  - definition of 'center values' as median or average;
  - definition of error bars as s.d. or s.e.m.

Please complete ALL of the questions below.
Select "Not Applicable" only when the requested information is not relevant for your study.

Materials

Newly Created Materials	Information included in the manuscript?	In which section is the information available? (Reagents and Tools Table, Materials and Methods, Figures, Data Availability Section)
New materials and reagents need to be available; do any restrictions apply?	Yes	Materials and Methods

Antibodies	Information included in the manuscript?	In which section is the information available? (Reagents and Tools Table, Materials and Methods, Figures, Data Availability Section)
For **antibodies** provide the following information:<br>- Commercial antibodies: RRID (if possible) or supplier name, catalogue number and or/clone number<br>- Non-commercial: RRID or citation	Yes	Materials and Methods

DNA and RNA sequences	Information included in the manuscript?	In which section is the information available? (Reagents and Tools Table, Materials and Methods, Figures, Data Availability Section)
**Short novel DNA or RNA including primers, probes**: provide the sequences.	Yes	Materials and Methods and Table EV2

Cell materials	Information included in the manuscript?	In which section is the information available? (Reagents and Tools Table, Materials and Methods, Figures, Data Availability Section)
**Cell lines:** Provide species information, strain. Provide accession number in repository **OR** supplier name, catalog number, clone number, and/**OR** RRID.	Yes	Materials and Methods
**Primary cultures:** Provide species, strain, sex of origin, genetic modification status.	Yes	Materials and Methods
Report if the cell lines were recently **authenticated** (e.g., by STR profiling) and tested for mycoplasma contamination.	Yes	Materials and Methods

Experimental animals	Information included in the manuscript?	In which section is the information available? (Reagents and Tools Table, Materials and Methods, Figures, Data Availability Section)
**Laboratory animals or Model organisms:** Provide species, strain, sex, age, genetic modification status. Provide accession number in repository **OR** supplier name, catalog number, clone number, **OR** RRID.	Yes	Materials and Methods
**Animal observed in or captured from the field:** Provide species, sex, and age where possible.	Not Applicable	
Please detail **housing and husbandry conditions**.	Yes	Materials and Methods

Plants and microbes	Information included in the manuscript?	In which section is the information available? (Reagents and Tools Table, Materials and Methods, Figures, Data Availability Section)
**Plants:** provide species and strain, ecotype and cultivar where relevant, unique accession number if available, and source (including location for collected wild specimens).	Not Applicable	
**Microbes:** provide species and strain, unique accession number if available, and source.	Not Applicable	

Human research participants	Information included in the manuscript?	In which section is the information available? (Reagents and Tools Table, Materials and Methods, Figures, Data Availability Section)
If collected and within the bounds of privacy constraints report on age, sex and gender or ethnicity for all study participants.	Yes	Results and Table EV1

Core facilities	Information included in the manuscript?	In which section is the information available? (Reagents and Tools Table, Materials and Methods, Figures, Data Availability Section)
If your work benefited from core facilities, was their service mentioned in the acknowledgments section?	Not Applicable	

Design

Study protocol	Information included in the manuscript?	In which section is the information available? (Reagents and Tools Table, Materials and Methods, Figures, Data Availability Section)
If study protocol has been **pre-registered, provide DOI in the manuscript**. For clinical trials, provide the trial registration number **OR** cite DOI.	Not Applicable	
Report the **clinical trial registration number** (at ClinicalTrials.gov or equivalent), where applicable.	Not Applicable	

Laboratory protocol	Information included in the manuscript?	In which section is the information available? (Reagents and Tools Table, Materials and Methods, Figures, Data Availability Section)
Provide DOI OR other citation details if **external detailed step-by-step protocols** are available.	Yes	Materials and Methods

Experimental study design and statistics	Information included in the manuscript?	In which section is the information available? (Reagents and Tools Table, Materials and Methods, Figures, Data Availability Section)
Include a statement about **sample size** estimate even if no statistical methods were used.	Yes	Materials and Methods
Were any steps taken to minimize the effects of subjective bias when allocating animals/samples to treatment (e.g. **randomization procedure**)? If yes, have they been described?	Yes	Materials and Methods
Include a statement about **blinding** even if no blinding was done.	Yes	Materials and Methods
Describe **inclusion/exclusion criteria** if samples or animals were excluded from the analysis. Were the criteria pre-established?<br><br>If sample or data points were omitted from analysis, report if this was due to attrition or intentional exclusion and provide justification.	Yes	Materials and Methods
For every figure, are **statistical tests** justified as appropriate? Do the data meet the assumptions of the tests (e.g., normal distribution)? Describe any methods used to assess it. Is there an estimate of variation within each group of data? Is the variance similar between the groups that are being statistically compared?	Yes	Materials and Methods, Figures, Figure legends

Sample definition and in-laboratory replication	Information included in the manuscript?	In which section is the information available? (Reagents and Tools Table, Materials and Methods, Figures, Data Availability Section)
In the figure legends: state number of times the experiment was **replicated** in laboratory.	Yes	Figure legends
In the figure legends: define whether data describe **technical or biological replicates**.	Yes	Figure legends

Ethics

Ethics	Information included in the manuscript?	In which section is the information available? (Reagents and Tools Table, Materials and Methods, Figures, Data Availability Section)
Studies involving **human participants**: State details of **authority granting ethics approval** (IRB or equivalent committee(s), provide reference number for approval.	Not Applicable	
Studies involving **human participants**: Include a statement confirming that **informed consent** was obtained from all subjects and that the experiments conformed to the principles set out in the WMA Declaration of Helsinki and the Department of Health and Human Services Belmont Report.	Yes	Materials and Methods
Studies involving **human participants: For publication of patient photos**, include a statement confirming that consent to publish was obtained.	Yes	Materials and Methods
Studies involving experimental **animals**: State details of **authority granting ethics approval** (IRB or equivalent committee(s), provide reference number for approval. Include a statement of compliance with ethical regulations.	Yes	Materials and Methods
Studies involving **specimen and field samples:** State if relevant **permits** obtained, provide details of authority approving study; if none were required, explain why.	Not Applicable	

Dual Use Research of Concern (DURC)	Information included in the manuscript?	In which section is the information available? (Reagents and Tools Table, Materials and Methods, Figures, Data Availability Section)
Could your study fall under dual use research restrictions? Please check biosecurity documents and list of **select agents and toxins** (CDC): https://www.selectagents.gov/sat/list.htm	Not Applicable	
If you used a select agent, is the security level of the lab appropriate and reported in the manuscript?	Not Applicable	
If a study is subject to dual use research of concern regulations, is the name of the **authority granting approval and reference number** for the regulatory approval provided in the manuscript?	Not Applicable	

Reporting

The MDAR framework recommends adoption of discipline-specific guidelines, established and endorsed through community initiatives. Journals have their own policy about requiring specific guidelines and recommendations to complement MDAR.

Adherence to community standards	Information included in the manuscript?	In which section is the information available? (Reagents and Tools Table, Materials and Methods, Figures, Data Availability Section)
State if relevant guidelines or checklists (e.g., **ICMJE, MIBBI, ARRIVE, PRISMA**) have been followed or provided.	Yes	Materials and Methods
For **tumor marker prognostic studies,** we recommend that you follow the **REMARK** reporting guidelines (see link list at top right). See author guidelines, under 'Reporting Guidelines'. Please confirm you have followed these guidelines.	Not Applicable	
For **phase II and III randomized controlled trials**, please refer to the **CONSORT** flow diagram (see link list at top right) and submit the CONSORT checklist (see link list at top right) with your submission. See author guidelines, under 'Reporting Guidelines'. Please confirm you have submitted this list.	Not Applicable	

Data Availability

Data availability	Information included in the manuscript?	In which section is the information available? (Reagents and Tools Table, Materials and Methods, Figures, Data Availability Section)
Have **primary datasets** been deposited according to the journal's guidelines (see 'Data Deposition' section) and the respective accession numbers provided in the Data Availability Section?	Not Applicable	
Were **human clinical and genomic datasets** deposited in a public access-controlled repository in accordance to ethical obligations to the patients and to the applicable consent agreement?	Yes	Materials and Methods
Are **computational models** that are central and integral to a study available without restrictions in a machine-readable form? Were the relevant accession numbers or links provided?	Not Applicable	
If publicly available data were reused, provide the respective **data citations in the reference list**.	Not Applicable	
